# Experimental and clinical data analysis for identification of COVID-19 resistant ACE2 mutations

**DOI:** 10.1038/s41598-022-20773-9

**Published:** 2023-02-09

**Authors:** Pawan Kumar Raghav, Aditya Raghav, Anjali Lathwal, Archit Saxena, Zoya Mann, Manisha Sengar, Raja Rajalingam

**Affiliations:** 1 Immunogenetics and Transplantation Laboratory, Department of Surgery, University of California San Francisco, San Francisco, CA USA; 2 BioExIn, Delhi, India; 3 Department of Computational Biology, Indraprastha Institute of Information Technology, New Delhi, India; 4 Amity Institute of Biotechnology, Amity University, Sector-125, Noida, Uttar Pradesh India; 5 Department of Zoology, Deshbandhu College, University of Delhi, Delhi, India

**Keywords:** Cell biology, Computational biology and bioinformatics, Structural biology

## Abstract

The high magnitude zoonotic event has caused by Severe Acute Respitarory Syndrome CoronaVirus-2 (SARS-CoV-2) is Coronavirus Disease-2019 (COVID-19) epidemics. This disease has high rate of spreading than mortality in humans. The human receptor, Angiotensin-Converting Enzyme 2 (ACE2), is the leading target site for viral Spike-protein (S-protein) that function as binding ligands and are responsible for their entry in humans. The patients infected with COVID-19 with comorbidities, particularly cancer patients, have a severe effect or high mortality rate because of the suppressed immune system. Nevertheless, there might be a chance wherein cancer patients cannot be infected with SARS-CoV-2 because of mutations in the ACE2, which may be resistant to the spillover between species. This study aimed to determine the mutations in the sequence of the human ACE2 protein and its dissociation with SARS-CoV-2 that might be rejecting viral transmission. The in silico approaches were performed to identify the impact of SARS-CoV-2 S-protein with ACE2 mutations, validated experimentally, occurred in the patient, and reported in cell lines. The identified changes significantly affect SARS-CoV-2 S-protein interaction with ACE2, demonstrating the reduction in the binding affinity compared to SARS-CoV. The data presented in this study suggest ACE2 mutants have a higher and lower affinity with SARS-Cov-2 S-protein to the wild-type human ACE2 receptor. This study would likely be used to report SARS-CoV-2 resistant ACE2 mutations and can be used to design active peptide development to inactivate the viral spread of SARS-CoV-2 in humans.

## Introduction

The Severe Acute Respiratory Syndrome-CoronaVirus-2 (SARS-CoV-2)-infected new Coronavirus Disease-2019 (COVID-19) outbreak is still a significant pandemic^1^. Primarily, on 31 December 2019, a pneumonia case of Wuhan, China, was informed to China’s World Health Organization (WHO) Office^2^. After a month, the outbreak was declared a Public Health Emergency of International Concern. Later, on 11 February 2020, WHO named the disease, COVID-19, caused by SARS-CoV-2 infection^3^. This disease emerged throughout 216 countries, areas, or territories, registered with 251,788,329 cumulative cases and 5,077,907 deaths as on 14th November, 2021^4^. Also, other coronaviruses (CoV), such as SARS-CoV and NL63, too, have Spike-protein (S-protein) trimer (triSpike) that serves as a ligand for their binding to and entry through its human cells receptor, a terminal carboxypeptidase, Angiotensin-converting enzyme 2 (ACE2)^5^. Notably, the receptor-binding motif (residues 424–494) positioned in the receptor-binding domain (RBD, residues 306–527) of SARS-CoV S-protein is a significant site to interact with ACE2 that facilitates virus entry and inducing protective immunity^6^. The ACE2 function is lost due to CoV pathogenesis in human airway epithelial cells^7^. It has been reported previously that the comorbidity with outbreaks of SARS-CoV and MERS-CoV increases the risk of respiratory distress and mortality^7^. Also, the clinical investigation of COVID-19 infection suggested that the persons can be more prone to the infection associated with comorbidities, which have one underlying disease such as cancer, diabetes, hypertension, cardiovascular, cerebrovascular, and pulmonary^8^. Recent studies revealed that ACE2 is associated with several cancer types susceptible to SARS-CoV-2^9–11^.

Therefore, in cancer patients, identifying ACE2 mutations in cancer association and its binding with SARS-CoV-2 is importantly required to prevent COVID-19 infection and design a drug^12–14^.

**Figure 1:**
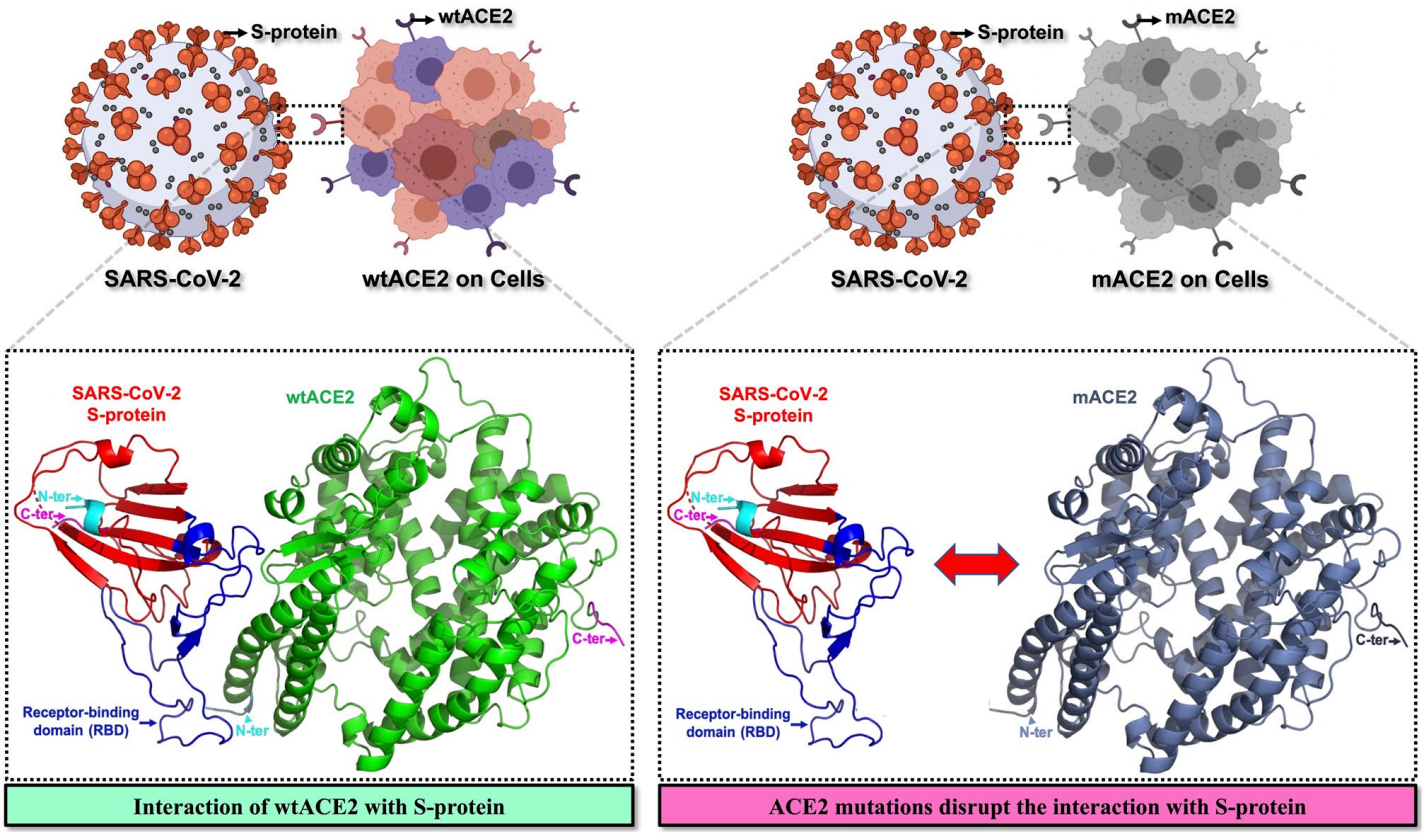
Interaction of wild-type ACE2 (wtACE2) and Spike protein (S-protein) of SARS-CoV-2. Pro-tumorigenic mutations of ACE2 (mACE2) were identified and the interactions of mACE2 with S-protein of SARS-CoV and SARS-CoV-2 were compared. A higher binding affinity between mACE2 and S-protein is suggestive of increased vulnerability towards COVID-19 infection, while a lower binding affinity might indicate decreased likelihood to contact with COVID-19 infection.

Several mutations fall in human ACE2 protein; among them, mutants with increased affinity support SARS-CoV entry, while low-affinity mutant blocks it^15^. It has been reported in a study that Chinese rhesus (rh) macaques are resistant to SARS-CoV infection and abandons its entry through ACE2^16^. Substantially, this alteration in the entry is because of the inability of RBD of SARS-CoV S-protein to interact with rhACE2’s natural mutant, Y217N, that also downregulates its expression^16^. Likewise, the mutational study on the human ACE2 mutant, Y217N, decreases the expression and entry of SARS-CoV^16^. Moreover, SARS-CoV S-protein binding has been demonstrated inhibited or completely lost with hACE2 mutations, QAK24-26KAE, K31D, Y41A, K68D, MYP82-84NFS, K353H, K353A, K353D, D355A, R357A, M383A, P389A, R393A, SPD425-427PSN, and R559S (Table 1)^17^. Oppositely, the interaction of SARS-CoV remains unaffected with ACE2 mutants, E37A, D38A, E110P, PD135-136SM, E160R, R192D, R219D, H239Q, K309D, E312A, T324A, NVQ338-340DDR, D350A, L359K, L359A, KGE465-467QDK, F603T^17^. Also, other mutations which do not directly alter the association of SARS-CoV with mutants R169Q, W271Q, R273Q, H345A, K481Q, H505A, and R514Q are responsible for the loss of enzyme activity or ACE cleavage, while L584A mutant inhibits the ACE cleavage^18–20^. Therefore, these mutations play a modulatory role in preventing SARS-CoV-2 infection (Fig. 1).

Several studies showed sequence mutation of SARS-CoV-2 that changed the binding capacity with ACE2, though no study reported the ACE2 mutations and their binding impact with SARS-CoV-2^15^. In this study, the experimental validated ACE2 mutants’ binding impact with SARS-CoV data was retrieved from the literature to compare the mutant's in-silico binding impact with SARS-CoV-2. The experimental data were used to correlate the in silico prediction of clinical patients and cell line’s ACE2 mutants binding with SARS-CoV-2.

**Figure 2:**
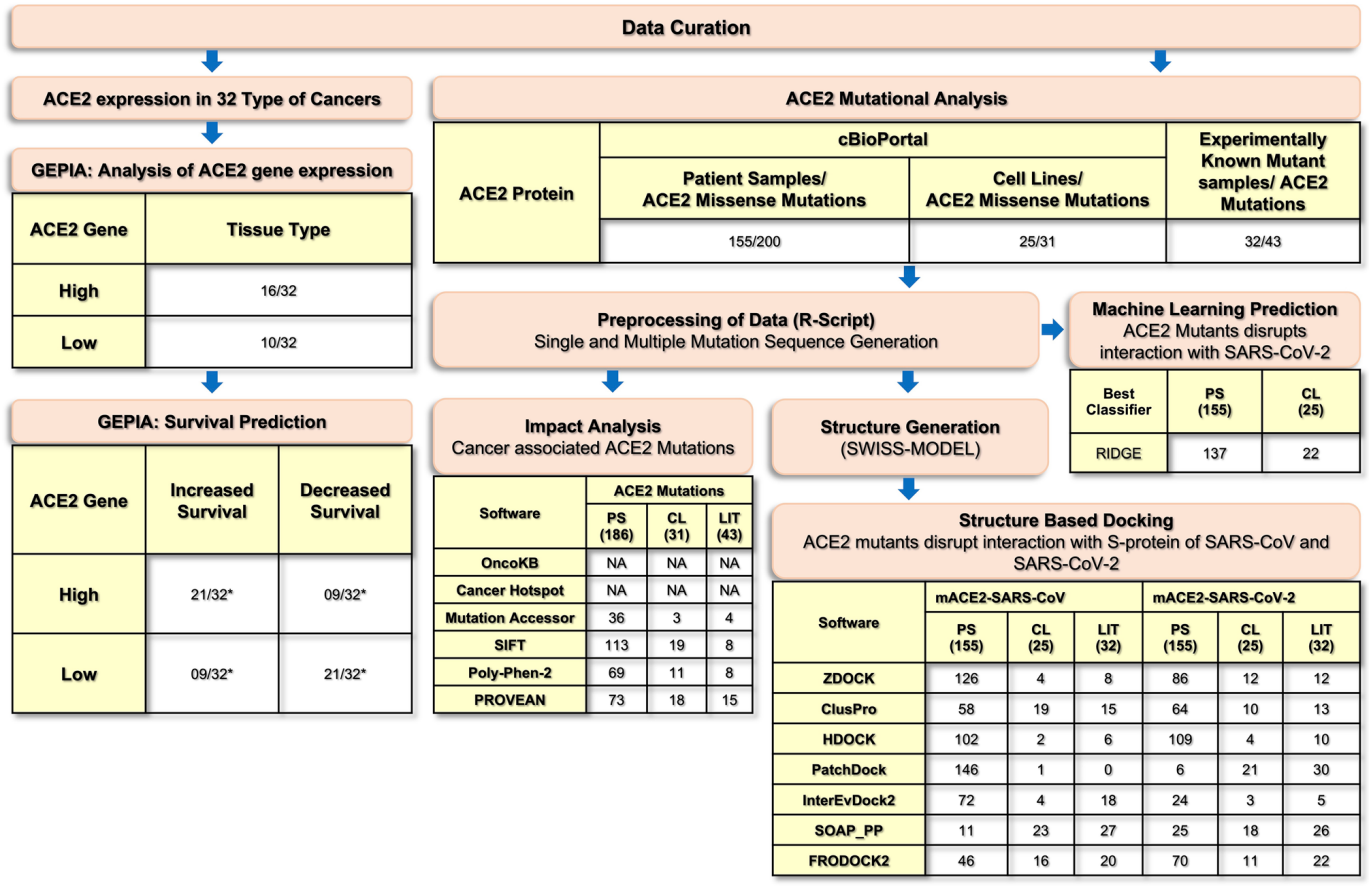
Workflow of the study represents identification of 200 ACE2 missense mutations in 155 patient samples, 31 mutations in 25 cell lines, which were curated from cBioPortal. In addition, 43 ACE2 mutations in 32 experimentally known mutant samples were retrieved from the literature. Further, preprocessing of the data was performed using in‐house R scripts wherein, frequency of sample ID, ACE2 mutations, and their single and multiple mutated sequences were generated. The ACE2 mutation impact within the cancer hotspots was predicted as medium, deleterious, and probably damaging by OncoKB, Cancer Hotspot, Mutation Assessor, SIFT, and PolyPhen‐2 for respective total numbers of patient samples (PS), cell lines (CL), literature (LIT). The 3D structures of mutated and wild‐type sequences of ACE2 were generated using SWISS‐MODEL webserver. Subsequently, structure‐based dockings were performed, and further the number of disrupted mutated ACE2 interactions with S-protein of SARS-CoV and SARS-CoV-2 was compared. Machine learning prediction identified that 137 out of 155 patient samples, and 22 out of 25 cell lines disrupt the interaction with SARS-CoV-2. *No impact of high/low ACE2 gene expression in predicting the overall survival of the patients was observed in 2/32 (BRCA and KICH).

To accomplish this, the information of experimentally verified SARS-CoV binding impact with ACE2 mutations was primarily collected from the literature (Fig. 2). Afterward, the reported patients and cell lines’ ACE2 mutations were retrieved from cBioPortal. Subsequently, the mutation data was processed to arrange the sample IDs according to unique mutations. Then sequences of ACE2 mutants were generated using an R script. These mutated and wild-type sequences were subjected to model 3D structures. Further, these 3D structures were used for docking and screening against SARS-CoV and SARS-CoV-2. Finally, the statistics were applied to identify the ACE2 mutant enrichments by identifying their association with cancer. The docking results identified the impact of mutants' interaction with SARS-CoV and SARS-CoV-2. Besides, the expression and overall survival analysis of ACE2 were also performed in 32 different cancer types. Finally, machine learning was applied to classify the ACE2 mutants, which promotes and dysregulates interaction with SARS-CoV and SARS-CoV-2^21^. Conclusively, results indicated that mutations could be the key to blocking the entry of SARS-CoV-2 through ACE2 and suggested designing vaccines and therapeutic drugs against COVID-19.

## Materials and methods

### ACE2 mutational data curation

The patient sample with ACE2 missense mutations in the different types of cancers was retrieved from cBioPortal until May 2020. Among 200 missense mutations, several single and multiple mutations were identified as unique in 155 patient samples^22^. Likewise, 31 mutations were found in 25 cell lines. Besides, the experimentally known binding impact of 32 ACE2 mutations with SARS-CoV was gathered from the literature review^17^.

### Pre-processing of ACE2 mutants data

The retrieved missense mutations of ACE2 were further pre-processed using in-house R scripts. The unique sample id and mutations, and generation of single and multiple mutated sequences of ACE2 were obtained for experimental known, patients, and cell lines samples.

### Identification of ACE2 mutations impact in cancer

The implications of mutations were analysed by considering the structural and functional effects on ACE2 protein. The former primarily affects attributes, such as the stability and fold of the protein product, and the latter affects functional sites^23–29^. The annotated impact of each ACE2 mutation in cancer was identified using OncoKB, Cancer Hotspot, 3D Hotspot^30–32^. Similarly, Mutation Assessor, Sorting Intolerant From Tolerant (SIFT), and PolyPhen‐2 accessed the functional impact of mutations^33–36^. These tools assigned a score to each ACE2 mutation based on the physical‐chemical properties or evolutionary conservation of the amino acid sequences affected by the modification.

### Three‐dimensional (3D) structure modeling of ACE2 mutants

The 3D structure of wild‐type and mutated, ACE2 protein was generated using SWISS‐MODEL^37^. To generate ACE2 3D structures of wild‐type and mutants, 6M18 (ELECTRON MICROSCOPY) as template was chosen by the SWISS‐MODEL tool to generate ACE2 3D structures of wild‐type and mutants. These 3D models were prepared by adding hydrogen atoms and charges considered further for docking studies. Moreover, the PDB ID, 3D0G, and 6M0J were used as SARS-CoV and SARS-CoV-2 3D structures.

### Docking predicted the binding affinity of ACE2 mutants with SARS-CoV-2

The binding of mutated ACE2 with SARS-CoV and SARS-CoV-2 was determined for experimental known, patient samples, and cell lines using docking. The structure‐based docking approach was used to evaluate the binding affinity between the mutated ACE2 and SARS-CoV and SARS-CoV-2. Structure‐based docking was performed using the 3D structure as the input. The ZDOCK, ClusPro, HDOCK, PatchDock, FireDock, InterEvDock2, SOAP-PP, and FRODOCK2 tools were used to perform structure‐based docking^38–42^. The HDOCK to calculate negative docking score and ligand rmsd (Å); ZDOCK to calculate positive docking score; ClusPro to calculate center‐weighted score; PatchDock to calculate positive score; FireDock to obtain global energy and obtained solution number of the complex used in PatchDock; InterEvDock2 to compute SOAP-PP (negative score), and InterEvScore and FRODOCK2, (positive scores).

### Machine learning

The Protr and Caret packages were used to perform the machine learning to classify the ACE2 mutation of patients and cell lines having potential in association or dissociation with S-protein of SARS-CoV-2^43,44^. Here, the experimentally validated 33 sequences (1 wild type and 32 mutants), in which 16, including wild-type, reported to have an affinity with S-protein, while 17 mutants with decreased or disrupted interaction were included as training and test dataset. We have used these 33 sequences in building our classification model. We have used the Protr package in R to calculate the various features of these peptides. These include Amino Acid Composition (AAC), Dipeptide Composition (DPC), Tripeptide Composition (TPC), Autocorrelation (Normalized Moreau-Broto, Moran and Geary), Conjoint Triad Descriptors (CTDs), and Composition enhanced Transition and Distribution (CeTD). All the features mentioned here have been tested independently in combination with other features to assess their classification capabilities. We have implemented various machine learning-based classification algorithms using the caret package as mentioned above. The algorithms implemented for this study are decision trees (DT), random forest (RF), multi-layer perceptron (MLP), eXtreme gradient boosting (XGBoost), K-nearest neighbors (KNN), support random vector with radial basis (SVR), neural network (NN), Ridge, Lasso, and Elastic Net”. Different parameters were optimized using “expand Grid” functionality of the “caret” package of R. 80:20 sampling has been done to create training and testing sets, respectively. The different classifiers were optimized using the leave one out cross-validation method, a recommended technique used with a small dataset like the one present in our study^45^. All these classifiers were implemented using an in-house R script. The performance of the developed model was evaluated using metrics such as specificity, sensitivity, accuracy, Matthews’s correlation coefficient (MCC), and area under the curve (AUC).

### Identification of ACE2 expression and survival analyses

Overall survival analyses of ACE2 based on gene expression levels were performed using gene expression profiling interactive analysis (GEPIA)^46^ in different 32 cancer types. The survival curves were generated based on median group cutoff as a threshold, which stratified the patients among high and low-risk groups^47,48^. The parameters used for evaluating the hypothesis and results are Hazards Ratio based on Log-rank test (Mantel–Cox test), p-value, and Confidence Interval at 95%. In addition, ACE2 expression was quantified in different cancer groups for their respective stages to identify the correlation with survival prediction analysis.

## Results

### Data analysis

The ACE2 mutation analysis used in this study to identify passenger and driver mutations that drive carcinogenesis and their impact on COVID-19 infectivity. In this study, the analysis of ACE2 missense mutations within different cancer types was mainly considered. The experimental known mutations and their interaction impact with SARS-CoV were retrieved from literature, while patient and cell lines data were collected from cBioPortal. The in vitro validated experimental interaction impact of single and multiple mutations with SARS-CoV were retrieved from literature (Table 1), whereas no experimental evidence was reported for SARS-CoV-2. Among 32 retrieved mutations, 15 ACE2 mutants abolished the interaction with SARS-CoV, while 17 mutants do not affect interaction with SARS-CoV. The 155 patient samples ID were retrieved from cBioPortal, containing 134 single points and 21 multiple ACE2 missense mutations in different cancer types (Table 2). Similarly, 31 ACE2 missense mutations have been identified in 25 cell lines (Table 3).

**Table 1:** Experimentally known ACE2 mutations and its binding impact on SARS-CoV.

S. no.	ACE2 mutation	Interaction with SARS-CoV	Feature
1	K31D	Inhibition	+
2	E37A	No effect	−
3	D38A	No effect	−
4	Y41A	Inhibition	+
5	K68D	Inhibition	+
6	E110P	No effect	−
7	E160R	No effect	−
8	R192D	No effect	−
9	R219D	No effect	−
10	H239Q	No effect	−
11	K309D	No effect	−
12	E312A	No effect	−
13	T324A	No effect	−
14	D350A	No effect	−
15	K353H	Inhibition	+
16	K353A	Inhibition	+
17	K353D	Inhibition	+
18	D355A	Inhibition	+
19	R357A	Inhibition	+
20	L359K	No effect	−
21	L359A	No effect	−
22	M383A	Inhibition	+
23	P389A	Inhibition	+
24	R393A	Inhibition	+
25	R559S	Inhibition	+
26	F603T	No effect	−
27	Q24K, A25A, K26E	Inhibition	+
28	M82N, Y83F, P84S	Inhibition	+
29	P135S, D136M	No effect	−
30	N338D, V339D, Q340R	No effect	−
31	S425P, P426S, D427N	Inhibition	+
32	K465Q, G466D, E467K	No effect	−
*	R169Q	Loss of angiotensin I cleavage	NA
*	W271Q	Loss of angiotensin I cleavage	NA
*	R273Q	Loss of enzyme activity	NA
*	H345A	Loss of enzyme activity	NA
*	K481Q	Loss of angiotensin I cleavage	NA
*	H505A	Loss of enzyme activity	NA
*	R514Q	Loss of angiotensin I cleavage	NA
*	L584A	Inhibits angiotensin I cleavage	NA

Here, * represents other mutations, which do not have direct effect on SARS-CoV binding.

**Table 2:** Patients’ ACE2 mutations retrieved from cBioPortal.

S. no.	Sample ID	Cancer type	ACE2 mutation
1	Pt15	Melanoma	L8F
2	ME009	Cutaneous melanoma	T20I
3	TCGA-ER-A19H-06	Cutaneous melanoma	E22D
4	coadread_dfci_2016_593	Colorectal adenocarcinoma	F28L
5	TCGA-99-7458-01	Lung adenocarcinoma	H34N
6	TCGA-B5-A3FA-01	Uterine endometrioid carcinoma	E35K
7	TCGA-VR-AA7B-01	Esophageal squamous cell carcinoma	E37K
8	TCGA-AP-A0LM-01	Uterine endometrioid carcinoma	L39M
9	TCGA-EE-A2MR-06	Cutaneous melanoma	S44L
10	coadread_dfci_2016_3646	Colorectal adenocarcinoma	V59D
11	TCGA-EO-A3B0-01	Uterine endometrioid carcinoma	F72C
12	TCGA-UF-A719-01	Head and neck squamous cell carcinoma	L73S
13	TCGA-62-A46R-01	Lung adenocarcinoma	A99S
14	YUZINO	Cutaneous melanoma	S109L
15	coadread_dfci_2016_1794, TCGA-FF-A7CW-01	Colorectal adenocarcinoma, diffuse large B-cell lymphoma, NOS	R115Q
16	TCGA-EY-A5W2-01	Uterine endometrioid carcinoma	R115W
17	TCGA-ZF-A9RG-01	Bladder urothelial carcinoma	L116F
18	TCGA-EE-A3AG-06	Cutaneous melanoma	P138S
19	TCGA-39–5035-01	Lung squamous cell carcinoma	G147V
20	TCGA-AX-A1CE-01, TCGA-D8-A1JG-01	Uterine endometrioid carcinoma, breast invasive ductal carcinoma	L162F
21	TCGA-NA-A5I1-01	Uterine carcinosarcoma/uterine malignant mixed Mullerian tumor	E182D
22	Pt26	Melanoma	E189K
23	TCGA-EE-A29N-06	Cutaneous melanoma	H195Y
24	TCGA-VQ-A8E7-01	Tubular stomach adenocarcinoma	Y202H
25	TCGA-AP-A059-01	Uterine endometrioid carcinoma	R204I
26	PR4046, PR4046_T	Melanoma	G205V
27	coadread_dfci_2016_3094	Colorectal adenocarcinoma	D206Y
28	TCGA-44-7670-01	Lung adenocarcinoma	G211W
29	coadread_dfci_2016_1254	Colorectal adenocarcinoma	V212I
30	coadread_dfci_2016_197	Colorectal adenocarcinoma	D213G
31	TCGA-44-2659-01	Lung adenocarcinoma	R219P
32	TCGA-BR-4292-01	Stomach adenocarcinoma	R219H
33	MO_1072	Penile squamous cell carcinoma	G220C
34	HN_62652	Head and neck squamous cell carcinoma	E232K
35	CLL-GCLL-0034-Tumor-SM-41JMX	Chronic lymphocytic leukemia/small lymphocytic lymphoma	A242T
36	TCGA-95-7948-01, TCGA-HC-A76W-01	Lung adenocarcinoma, prostate adenocarcinoma	I256M
37	MO_1111	Lung adenocarcinoma	A264S
38	5-VS009-T2	Skin cancer, non-melanoma	D269N
39	TCGA-A5-A0G1-01	Uterine serous carcinoma/uterine papillary serous carcinoma	D269Y
40	TCGA-D3-A2JB-06	Cutaneous melanoma	G272C
41	TCGA-D1-A103-01	Uterine endometrioid carcinoma	R273K
42	TCGA-AX-A05Z-01	Uterine endometrioid carcinoma	S280Y
43	TCGA-2J-AABP-01	Pancreatic adenocarcinoma	V293I
44	TCGA-BA-A6DA-01	Head and neck squamous cell carcinoma	A296T
45	H104362	Hepatocellular adenoma	Q305L
46	TCGA-D3-A8GI-06	Cutaneous melanoma	A311V
47	TCGA-FR-A729-06	Cutaneous melanoma	S317F
48	TCGA-69-7980-01	Lung adenocarcinoma	L320F
49	TCGA-AA-A01P-01	Colon adenocarcinoma	T324S
50	TCGA-CU-A0YR-01	Bladder urothelial carcinoma	Q325P
51	TCGA-D7-6528-01	Tubular stomach adenocarcinoma	N330H
52	MO_1288	Breast invasive ductal carcinoma	T334R
53	MEL-IPI_Pat74-Tumor-SM-4DK2Z, Pat74, TCGA-AA-3977-01	Cutaneous melanoma, cutaneous melanoma, colon adenocarcinoma	G337E
54	TCGA-VQ-A8P2-01	Mucinous stomach adenocarcinoma	N338D
55	coadread_dfci_2016_341	Colorectal adenocarcinoma	G352W
56	MEL-IPI_Pat11-Tumor-SM-4DK17, Pat11	Melanoma of unknown primary, cutaneous melanoma	D355N
57	LUAD-LIP77	Lung adenocarcinoma	R357S
58	TCGA-06-1801-01	Glioblastoma multiforme	I358F
59	TCGA-AA-3947-01	Mucinous adenocarcinoma of the colon and rectum	V364A
60	WA48	Prostate adenocarcinoma	D367V
61	Pat_22_Post, Pat_22_Pre	Melanoma	D368N
62	TCGA-EE-A20F-06	Cutaneous melanoma	M383I
63	Au8	Desmoplastic melanoma	R393G
64	TCGA-56-7731-01	Lung squamous cell carcinoma	G395V
65	585208	Small cell lung cancer	E398K
66	YULAN	Cutaneous melanoma	G399R
67	CRUK0001-R1, CRUK0001-R2, CRUK0001-R3	Non-small cell lung cancer	A403V
68	H072511	Hepatocellular adenoma	G405W
69	MEL-JWCI-WGS-12	Cutaneous melanoma	E406K
70	TCGA-GN-A26C-01	Cutaneous melanoma	S409L
71	TCGA-BR-7707-01	Stomach adenocarcinoma	A412T
72	TCGA-BS-A0UF-01	Uterine endometrioid carcinoma	K419T
73	CSCC-27-T	Cutaneous squamous cell carcinoma	P426L
74	LSD4744, LSD4744_T	Cutaneous melanoma	P426S
75	19424739	Renal non-clear cell carcinoma	D431G
76	TCGA-24-1564-01	Serous ovarian cancer	L450P
77	TCGA-D7-6527-01	Papillary stomach adenocarcinoma	K458T
78	MO_1433	Lung adenocarcinoma	M462I
79	coadread_dfci_2016_3498	Colorectal adenocarcinoma	I468T
80	TCGA-QK-A8Z8-01	Head and neck squamous cell carcinoma	W473L
81	TCGA-92-7341-01	Lung squamous cell carcinoma	W477R
82	MEL-IPI_Pat62-Tumor-SM-4DK2N, Pat62	Cutaneous melanoma, cutaneous melanoma	V488M
83	TCGA-Z2-A8RT-06	Cutaneous melanoma	E489K
84	TCGA-22-1016-01	Lung squamous cell carcinoma	V491L
85	TCGA-AA-A022-01	Colon adenocarcinoma	D494G
86	TCGA-ZJ-AB0H-01	Cervical squamous cell carcinoma	T496A
87	TCGA-A5-A1OF-01	Uterine mixed endometrial carcinoma	R518M
88	5-NB008-T1	Skin cancer, non-melanoma	G561R
89	5-PT027-T1	Skin cancer, non-melanoma	P565L
90	TCGA-A5-A0G2-01	Uterine serous carcinoma/uterine papillary serous carcinoma	K577N
91	PCNSL_4	Diffuse large B-cell lymphoma, NOS	M579T
92	TCGA-06-6389-01	Glioblastoma multiforme	V581I
93	TCGA-F1-6874-01	Intestinal type stomach adenocarcinoma	P590L
94	TCGA-ND-A4WC-01	Uterine carcinosarcoma/uterine malignant mixed Mullerian tumor	D597E
95	FR9547	Lung adenocarcinoma	N599K
96	TCGA-A5-A2K5-01	Uterine endometrioid carcinoma	K600N
97	DFCI-CLL139-Tumor	Chronic lymphocytic leukemia/small lymphocytic lymphoma	N601I
98	TCGA-AA-3984-01	Colon adenocarcinoma	D609N
99	TCGA-AU-6004-01	Colon adenocarcinoma	Y613H
100	SJERG016_D_WES	Acute lymphoid leukemia	D615Y
101	TCGA-EK-A2RN-01	Cervical squamous cell carcinoma	I618M
102	TCGA-AA-A00N-01	Mucinous adenocarcinoma of the colon and rectum	K625T
103	TCGA-AA-A010-01	Colon adenocarcinoma	L628F
104	TCGA-BR-4201-01	Stomach adenocarcinoma	R644Q
105	MO_1146	Cutaneous melanoma	E667K
106	TCGA-55-8506-01	Lung adenocarcinoma	V670L
107	MSKCC-0411_R	Bladder urothelial carcinoma	V672A
108	CRUK0027-R2	Non-small cell lung cancer	K676E
109	TCGA-AG-3892-01, TCGA-F5-6814-01	Rectal adenocarcinoma, rectal adenocarcinoma	F683L
110	TCGA-L5-A8NQ-01	Esophageal squamous cell carcinoma	S692F
111	TCGA-73-4658-01	Lung adenocarcinoma	D693N
112	TCGA-CR-6484-01	Head and neck squamous cell carcinoma	I694M
113	5-VS045-T1	Skin cancer, non-melanoma	E701K
114	coadread_dfci_2016_102, TCGA-RD-A8NB-01	Colorectal adenocarcinoma, diffuse type stomach adenocarcinoma	R708Q
115	DLBCL-RICOVER_1150	Activated B-cell type	D713N
116	coadread_dfci_2016_3064	Colorectal adenocarcinoma	R716H
117	TCGA-HZ-7922-01	Pancreatic adenocarcinoma	R716C
118	OS-47-SJ	Osteosarcoma	N720S
119	CHC2128T	Hepatocellular carcinoma	P737H
120	CSCC-31-T	Cutaneous squamous cell carcinoma	P737L
121	NCH-CA-3	Colorectal adenocarcinoma	V748F
122	TCGA-AJ-A3BH-01	Uterine endometrioid carcinoma	L760M
123	AMPAC_719	Ampullary carcinoma	I761T
124	TCGA-4A-A93Y-01	Papillary renal cell carcinoma	F762L
125	5-PT001-T1	Skin cancer, non-melanoma	G764R
126	TCGA-19-1390-01	Glioblastoma multiforme	R766K
127	LUAD_E00522	Lung adenocarcinoma	R768L
128	TCGA-E6-A1LX-01	Uterine endometrioid carcinoma	R768W
129	TCGA-BS-A0UJ-01	Uterine endometrioid carcinoma	R775I
130	TCGA-ER-A1A1-06	Cutaneous melanoma	P780S
131	SC_9081-TM, YURED	Prostate adenocarcinoma, cutaneous melanoma	D785N
132	Pt1	Melanoma	G789R
133	TCGA-55-8205-01	Lung adenocarcinoma	T798P
134	TCGA-VS-A958-01	Cervical squamous cell carcinoma	T803I
135	587376	Colorectal adenocarcinoma	S47C, P284S
136	5-VS022-T1	Skin cancer, non-melanoma	E145K, E639K
137	coadread_dfci_2016_116	Colorectal adenocarcinoma	W302G, F400L
138	Pat_41_Post	Melanoma	T593I, P729S
139	TCGA-AP-A1E0-01	Uterine endometrioid carcinoma	H195Y, F683L
140	TCGA-AX-A0J0-01	Uterine endometrioid carcinoma	K131Q, F683L
141	TCGA-B5-A1MR-01	Uterine endometrioid carcinoma	L120I, V658L
142	TCGA-EE-A183-06	Cutaneous melanoma	S280Y, Q598H
143	TCGA-FI-A2D5-01	Uterine endometrioid carcinoma	D427N, A576T
144	TCGA-AP-A1DK-01	Uterine endometrioid carcinoma	W48L, N437H
145	TCGA-B5-A11H-01	Uterine endometrioid carcinoma	P336S, K26N
146	TCGA-EO-A22R-01	Uterine endometrioid carcinoma	N578S, Y497C
147	TCGA-06-5416-01	Glioblastoma multiforme	P178S, E182D, D427N
148	TCGA-AG-A002-01	Rectal adenocarcinoma	R169I, H195Y, N394H
149	TCGA-AP-A056-01	Uterine endometrioid carcinoma	N194K, R306I, E479D
150	TCGA-B5-A11E-01	Uterine endometrioid carcinoma	S128I, R169I, S602Y
151	TCGA-BS-A0UV-01	Uterine endometrioid carcinoma	M82T, F314L, K600N
152	TCGA-D1-A17Q-01	Uterine endometrioid carcinoma	E375D, K577N, R768W
153	TCGA-D3-A2JP-06	Cutaneous melanoma	Q18K, G268C, W610L
154	TCGA-DU-6392-01	Astrocytoma	A25V, A396T, I679N
155	TCGA-EO-A22U-01	Uterine endometrioid carcinoma	R393I, E571G, R768W

**Table 3:** ACE2 mutations in cell lines retrieved from cBioPortal.

S. no.	Sample ID	Cancer type	ACE2 mutation
1	JSC1_HAEMATOPOIETIC_AND_LYMPHOID_TISSUE	Mixed cancer types	S5F
2	ISHIKAWAHERAKLIO02ER_ENDOMETRIUM	Mixed cancer types	A25V
3	OSRC2_KIDNEY	Mixed cancer types	L100V
4	JHOS2_OVARY	Mixed cancer types	V184A
5	HEC59_ENDOMETRIUM	Mixed cancer types	S218N
6	A172_CENTRAL_NERVOUS_SYSTEM	Mixed cancer types	Y252C
7	NCIH513_PLEURA	Mixed cancer types	P253T
8	SUDHL10_HAEMATOPOIETIC_AND_LYMPHOID_TISSUE	Mixed cancer types	T276K
9	MCC26_SKIN	Mixed cancer types	N322I
10	CAL54_KIDNEY	Mixed cancer types	T334A
11	LS123_LARGE_INTESTINE	Mixed cancer types	A413V
12	EN_ENDOMETRIUM	Mixed cancer types	K416N
13	LU165_LUNG	Mixed cancer types	P426L
14	GMEL_SKIN	Mixed cancer types	E457K
15	LS411N_LARGE_INTESTINE	Mixed cancer types	Q472P
16	TMK1_STOMACH	Mixed cancer types	P612L
17	CORL32_LUNG	Mixed cancer types	W635L
18	HCT_15; HCT15_LARGE_INTESTINE	Colorectal adenocarcinoma, mixed cancer types	Y649C
19	PECAPJ15_UPPER_AERODIGESTIVE_TRACT	Mixed cancer types	E668K
20	MESSA_SOFT_TISSUE	Mixed cancer types	A782V
21	CW2_LARGE_INTESTINE	Mixed cancer types	A386T, F314I
22	HCC_2998; HCC2998_LARGE_INTESTINE	Colorectal adenocarcinoma; mixed cancer types	F603C, K619N
23	HEC251_ENDOMETRIUM	Mixed cancer types	F314L, Y510H
24	JHUEM7_ENDOMETRIUM	Mixed cancer types	L664I, D382Y
25	MCC13_SKIN	Mixed cancer types	E145K, E495K, I233S

### ACE2 mutation impact identifies the cancer hotspots

Mutations within tumor suppressors and oncogenes might be benign or deleterious, based on the mutation site and whether it is a missense or non-sense mutation. The impact of a mutation on the functionality of the respective protein can be predicted using different tools. This assists in classifying between functional driver and passenger mutations based on the frequency of occurrence and the relevant consequence of each mutation in tumor development^12,14^. The impact prediction scores of the experimental, cell line, and patient-specific data were calculated by four software, including Mutation accessor, SIFT, PolyPhen-2, and PROVEAN. These prediction scores were analyzed to predict the cancer hotspots within all 3 data sets.

#### Impact of known experimental mutations in cancer association

The experimental data identified 26 single mutants and 6 multi-mutants analyzed by four different tools to predict their activity in affecting tumor development (Table 4). Mutants with impact scores ranging 3.7 and above were classified with high risk by the Mutation Assessor tool. 4 single mutants, E312A, P389A, D355A, and D350A, were predicted with a high impact score, with the highest score observed for D355A mutant. 2 single mutants, E37A and T324A, with a medium impact score and only D38A mutant with a low impact score. H239Q and R559S mutants revealed a neutral impact score. However, predicting the scores for as many as 17 mutants, including K31D, Y41A, K68D, E110P, E160R, R192D, R219D, K309D, K353H, K353A, K353D, R357A, L359K, L359A, M383A, R393A, and F603T, was beyond the scope of this tool. All the six multi-mutants, Q24K/A25A/K26E, M82N/Y83F/P84S, P135S/D136M, N338D/V339D/Q340R, S425P/P426S/D427N, and K465Q/G466D/E467K, displayed a neutral or low impact based on the prediction scores.

**Table 4:** Impact of experimental known mutations in cancer.

ACE2 mutation	Mutation Assessor (impact and score)	SIFT (impact and score)	PROVEAN (prediction; score)	PolyPhen-2 (impact and score)
Wild Type	NA	NA	NA	NA
K31D	NA	Tolerated; 0.57	Neutral; − 0.338	Benign; 0.004
E37A	Medium; 2.13	Tolerated; 0.68	Deleterious; − 3.096	Benign; 0.352
D38A	Low; 1.78	Tolerated; 0.66	Neutral; − 1.532	Benign; 0.060
Y41A	NA	Tolerated; 0.58	Deleterious; − 3.325	Probably damaging; 0.988
K68D	NA	Tolerated; 0.46	Neutral; − 2.048	Benign; 0.135
E110P	NA	Tolerated; 0.19	Neutral; − 0.727	Benign; 0.000
E160R	NA	Tolerated; 0.35	Deleterious; − 3.914	Benign; 0.039
R192D	NA	Deleterious; 0.02	Deleterious; − 3.248	Possibly damaging; 0.814
R219D	NA	Tolerated; 0.52	Neutral; − 1.803	Possibly damaging; 0.773
H239Q	Neutral; − 0.64	Tolerated; 0.86	Neutral; 0.592	Benign; 0.003
K309D	NA	Tolerated; 0.21	Deleterious; − 2.999	Possibly damaging; 0.849
E312A	High; 3.78	Tolerated; 0.09	Deleterious; − 4.805	Probably damaging; 0.998
T324A	Medium; 3.005	Tolerated; 0.10	Neutral; − 1.298	Possibly damaging; 0.668
D350A	High; 3.945	Deleterious; 0.00	Deleterious; − 7.944	Probably damaging; 1.000
K353H	NA	Tolerated; 0.20	Neutral; − 1.874	Benign; 0.160
K353A	NA	Tolerated; 0.36	Deleterious; − 2.796	Possibly damaging; 0.851
K353D	NA	Tolerated; 0.26	Neutral; − 1.249	Probably damaging; 0.996
D355A	High; 3.985	Deleterious; 0.00	Deleterious; − 7.606	Probably damaging; 1.000
R357A	NA	Deleterious; 0.00	Deleterious; − 5.956	Probably damaging; 1.000
L359K	NA	Tolerated; 1.00	Neutral; 6.101	Benign; 0.002
L359A	NA	Deleterious; 0.02	Neutral; 0.712	Benign; 0.026
M383A	NA	Deleterious; 0.02	Deleterious; − 4.797	Probably damaging; 1.000
P389A	High; 3.915	Deleterious; 0.04	Deleterious; − 7.005	Probably damaging; 0.991
R393A	NA	Deleterious; 0.00	Deleterious; − 5.609	Probably damaging; 1.000
R559S	Neutral; − 0.7	Tolerated; 0.66	Neutral; − 1.171	Benign; 0.001
F603T	NA	Tolerated; 0.48	Neutral; 0.831	Benign; 0.004
Q24K, A25A, K26E	Neutral; 0.325, NA, Low; 1.195	Tolerated; 0.98, Tolerated; 1.00, Tolerated; 0.31	Neutral; − 1.315, Neutral; 0, Neutral; − 1.27	Benign; 0.000, NA, Benign; 0.000
M82N, Y83F, P84S	NA, Neutral; − 0.095, Low; 0.835	Tolerated; 0.45, Tolerated; 1.00, Tolerated; 0.73	Neutral; 0.726, Neutral; 0.236, Neutral; − 0.703	Benign; 0.000, Benign; 0.000, Benign; 0.001
P135S, D136M	Low; 1.23, NA	Tolerated; 0.84, Tolerated; 0.14	Neutral; − 1.216, Deleterious; − 3.047	Benign; 0.001, Benign; 0.014
N338D, V339D, Q340R	Neutral; − 2.73, Neutral; − 1.7, Neutral; − 2.475	Tolerated; 1.00, Tolerated; 0.23, Tolerated; 1.00	Neutral; 4.122, Neutral; 2.499, Neutral; 3.548	Benign; 0.000, Benign; 0.000, Benign; 0.000
S425P, P426S, D427N	Neutral; − 0.965, Neutral; 0.235, Low; 1.435	Tolerated; 0.29, Tolerated; 1.00, Tolerated; 0.86	Neutral; − 0.021, Neutral; − 0.568, Neutral; − 0.822	Benign; 0.000, Benign; 0.004, Benign; 0.001
K465Q, G466D, E467K	Neutral; 0.74, NA, Neutral; 0.57	Tolerated; 0.49, Tolerated; 0.11, Tolerated; 0.56	Neutral; − 0.413, Deleterious; − 6.247, Neutral; − 0.48	Benign; 0.001, Benign; 0.354, Benign; 0.005

SIFT scores categorize the mutants into “tolerated” and “deleterious” based on the impact scores. Tolerated impact implies a neutral effect, while deleterious suggests the mutation affects the respective protein functionality. The experimental data predicted impact score ranging between 0–0.04 to possess a deleterious effect. Eight single mutants, R192D, D350A, D355A, R357A, R393A, L359A, M383A, and P389A, had deleterious impact scores, while 18 single mutants, K31D, E312A, T324A, E110P, K353H, K309D, K353D, E160R, K353A, K68D, F603T, R219D, Y41A, D38A, R559S, E37A, H239Q, and L359K, had a tolerated impact. Similarly, six multi-mutants, Q24K/A25A/K26E, M82N/Y83F/P84S, P135S/D136M, N338D/V339D/Q340R, S425P/P426S/D427N, and K465Q/G466D/E467K had a tolerated effect based on the SIFT scores.

PROVEAN prediction scoring, recognized more deleterious mutations, as many as 13, R192D, K353A, K309D, E37A, Y41A, E160R, M383A, E312A, R393A, R357A, P389A, D355A, and D350A. In addition, other 13 mutants, K31D, H239Q, L359A, E110P, F603T, R559S, K353D, T324A, D38A, R219D, K353H, K68D, and L359K, showed neutral impact scores suggestive of no change in the protein functionality of respective mutants. Similarly, four multi-mutants Q24K/A25A/K26E, M82N/Y83F/P84S, N338D/V339D/Q340R, and S425P/P426S/D427N, did not display any change in the protein functionality with a neutral impact score. However, P135S/D136M and K465Q/G466D/E467K multi-mutant had a neutral and deleterious impact score.

The PolyPhen-2 scoring divides the mutants into three classes on comparative analysis with the wild-type residue. Benign is predicted to have the least damage on the protein functionality, possibly damaging impairs the protein functionality with a low confidence prediction, and probably damaging that disrupts the protein functionality with the highest confidence score. The 12 single mutants, K31D, E110P, R559S, L359K, H239Q, F603T, L359A, E160R, D38A, K68D, K353H and E37A were classified as benign based on their impact score range 0–0.35. Five mutants, T324A, R219D, R192D, K309D, and K353A, had impact scores ranging 0.6–0.85 predicted to be possibly damaging. However, the maximum effect on protein functionality predicted with the highest confidence was based on the score range 0.9–1. The Nine single mutants, Y41A, P389A, K353D, E312A, D350A, D355A, R357A, M383A, and R393A, were identified to be probably damaging with the highest predicted impact. Six multi-mutants, Q24K/A25A/K26E, M82N/Y83F/P84S, P135S/D136M, N338D/V339D/Q340R, S425P/P426S/D427N and K465Q/G466D/E467K revealed to have a benign effect on the functionality of respective proteins.

#### Impact of patient mutations in cancer association

Out of the 155 mutants recognized on screening through the patient samples data, Mutation Assessor prediction scores categorized 24 mutants with a high impact factor (Table 5). These mutants, including 22 single mutants F72C, L162F, Y202H, R204I, A311V, S317F, D355N, R357S, I358F, V364A, R393G, G395V, G399R, A403V, G405W, L450P, W477R, G561R, P565L, M579T, P590L, and N599K, and two multi-mutants W302G/F400L and W48L/N437H had an impact score from 3.5 to 4.5 to be listed under high-risk category. A majority of the other mutants, 74 single mutants T20I, E22D, F28L, E37K, L39M, S44L, S109L, R115W, L116F, G147V, G205V, D206Y, R219H, E232K, A242T, I256M, A264S, D269Y, G272C, R273K, S280Y, V293I, L320F, T324S, N330H, T334R, G352W, D367V, M383I, E398K, E406K, S409L, A412T, K419T, P426L, D431G, K458T, M462I, W473L, E489K, D494G, T496A, R518M, V581I, N601I, Y613H, D615Y, I618M, K625T, L628F, E667K, V670L, V672A, K676E, F683L, S692F, I694M, E701K, R708Q, D713N, R716H, R716C, N720S, P737H, P737L, V748F, L760M, I761T, G764R, R768L, R768W, P780S, G789R and T803I, and six multi-mutants T593I/P729S, K131Q/F683L, L120I/V658L, P336S/K26N, N578S/Y497C and A25V/A396T/I679N, were identified with a medium impact score falling between 2 to 3.5. Twenty-five mutants L8F, E35K, V59D, P138S, E182D, E189K, G211W, D213G, R219P, D269N, A296T, G337E, D368N, I468T, V488M, K577N, K600N, D609N, R644Q, D693N, F762L, R766K, R775I, D785N and T798P had a low impact score, while 13 mutants H34N, L73S, A99S, R115Q, H195Y, V212I, G220C, Q305L, Q325P, N338D, P426S, V491L and D597E had a neutral score.

**Table 5:** Impact of patient mutations in cancer.

Sample ID	Cancer type	ACE2 mutations	OncoKB	Cancer hotspot	Mutation Assessor (impact and score)	SIFT (impact and score)	PROVEAN (prediction; score)	PolyPhen-2 (impact and score)
WILD TYPE_ACE2	–	–	–	–	–	–	–	–
Pt15	Melanoma	L8F	NA	No	Low; 0.95	Tolerated; 0.27	Neutral; − 1.206	Benign; 0.003
ME009	Cutaneous melanoma	T20I	NA	No	Medium; 2.735	Tolerated; 0.08	Neutral; − 1.553	Benign; 0.024
TCGA-ER-A19H-06	Cutaneous melanoma	E22D	NA	No	Medium; 3.075	Deleterious; 0.02	Neutral; − 1.267	Possibly damaging; 0.852
coadread_dfci_2016_593	Colorectal adenocarcinoma	F28L	NA	No	Medium; 2.855	Deleterious; 0.02	Deleterious; − 5.118	Probably damaging; 0.999
TCGA-99-7458-01	Lung adenocarcinoma	H34N	NA	No	Neutral; 0.09	Tolerated; 0.35	Neutral; 0.454	Benign; 0
TCGA-B5-A3FA-01	Uterine endometrioid carcinoma	E35K	NA	No	Low; 1.225	Tolerated; 0.72	Neutral; − 1.036	Benign; 0.013
TCGA-VR-AA7B-01	Esophageal squamous cell carcinoma	E37K	NA	No	Medium; 2.745	Deleterious; 0.04	Neutral; − 1.864	Possibly damaging; 0.523
TCGA-AP-A0LM-01	Uterine endometrioid carcinoma	L39M	NA	No	Medium; 2.47	Tolerated; 0.05	Neutral; − 0.618	Possibly damaging; 0.658
TCGA-EE-A2MR-06	Cutaneous melanoma	S44L	NA	No	Medium; 3.1	Deleterious; 0.05	Neutral; − 2.074	Probably damaging; 0.91
coadread_dfci_2016_3646	Colorectal adenocarcinoma	V59D	NA	No	Low; 1.045	Tolerated; 0.12	Neutral; − 1.05	Benign; 0.191
TCGA-EO-A3B0-01	Uterine endometrioid carcinoma	F72C	NA	No	High; 3.69	Deleterious; 0.01	Deleterious; − 5.186	Probably damaging; 0.99
TCGA-UF-A719-01	Head and neck squamous cell carcinoma	L73S	NA	No	Neutral; − 0.205	Tolerated; 0.22	Neutral; 0.916	Benign; 0.011
TCGA-62-A46R-01	Lung adenocarcinoma	A99S	NA	No	Neutral; 0.485	Tolerated; 0.83	Neutral; 0.599	Benign; 0.003
YUZINO	Cutaneous melanoma	S109L	NA	No	Medium; 2.845	Deleterious; 0.03	Deleterious; − 3.396	Possibly damaging; 0.786
coadread_dfci_2016_1794, TCGA-FF-A7CW-01	Colorectal adenocarcinoma; diffuse large B-cell lymphoma, NOS	R115Q	NA	No	Neutral; 0.025	Tolerated; 0.43	Neutral; 0.944	Benign; 0.007
TCGA-EY-A5W2-01	Uterine endometrioid carcinoma	R115W	NA	No	Medium; 2.8	Deleterious; 0	Deleterious; − 3.804	Possibly damaging; 0.848
TCGA-ZF-A9RG-01	Bladder urothelial carcinoma	L116F	NA	No	Medium; 2.42	Deleterious; 0.04	Neutral; − 0.463	Possibly damaging; 0.82
TCGA-EE-A3AG-06	Cutaneous melanoma	P138S	NA	No	Low; 1.745	Tolerated; 0.1	Neutral; − 1.755	Benign; 0.014
TCGA-39-5035-01	Lung squamous cell carcinoma	G147V	NA	No	Medium; 2.63	Deleterious; 0	Deleterious; − 4.349	Probably damaging; 0.966
TCGA-AX-A1CE-01, TCGA-D8-A1JG-01	Uterine endometrioid carcinoma; breast invasive ductal carcinoma	L162F	NA	No	High; 3.78	Deleterious; 0	Deleterious; − 2.992	Probably damaging; 0.995
TCGA-NA-A5I1-01	Uterine carcinosarcoma/uterine malignant mixed Mullerian tumor	E182D	NA	No	Low; 1.83	Tolerated; 0.27	Neutral; − 1.291	Benign; 0.003
Pt26	Melanoma	E189K	NA	No	Low; 1.59	Tolerated; 0.67	Neutral; − 1.05	Benign; 0.316
TCGA-EE-A29N-06	Cutaneous melanoma	H195Y	NA	No	Neutral; 0.69	Deleterious; 0	Neutral; − 1.761	Benign; 0.33
TCGA-VQ-A8E7-01	Tubular stomach adenocarcinoma	Y202H	NA	No	High; 3.61	Deleterious; 0.01	Deleterious; − 3.445	Probably damaging; 0.982
TCGA-AP-A059-01	Uterine endometrioid carcinoma	R204I	NA	No	High; 3.54	Deleterious; 0.02	Deleterious; − 7.03	Probably damaging; 0.996
PR4046, PR4046_T	Melanoma	G205V	NA	No	Medium; 2.28	Deleterious; 0.04	Neutral; − 2.456	Possibly damaging; 0.685
coadread_dfci_2016_3094	Colorectal adenocarcinoma	D206Y	NA	No	Medium; 2.495	Tolerated; 0.34	Neutral; − 1.591	Probably damaging; 0.952
TCGA-44-7670-01	Lung adenocarcinoma	G211W	NA	No	Low; 1.79	Tolerated; 0.19	Neutral; − 0.677	Benign; 0.017
coadread_dfci_2016_1254	Colorectal adenocarcinoma	V212I	NA	No	Neutral; 0.41	Tolerated; 0.4	Neutral; 0.121	Benign; 0
coadread_dfci_2016_197	Colorectal adenocarcinoma	D213G	NA	No	Low; 1.6	Tolerated; 0.1	Neutral; − 2.294	Benign; 0.003
TCGA-44–2659-01	Lung adenocarcinoma	R219P	NA	No	Low; 1.63	Deleterious; 0.01	Neutral; − 1.866	Probably damaging; 0.973
TCGA-BR-4292–01	Stomach adenocarcinoma	R219H	NA	No	Medium; 3.075	Deleterious; 0	Neutral; − 1.52	Probably damaging; 0.926
MO_1072	Penile squamous cell carcinoma	G220C	NA	No	Neutral; 0.345	Deleterious; 0	Neutral; − 1.063	Benign; 0.151
HN_62652	Head and neck squamous cell carcinoma	E232K	NA	No	Medium; 2.89	Deleterious; 0.04	Neutral; − 2.433	Benign; 0.044
CLL-GCLL-0034-Tumor-SM-41JMX	Chronic lymphocytic leukemia/small lymphocytic lymphoma	A242T	NA	No	Medium; 2.245	Tolerated; 0.05	Deleterious; − 3.03	Possibly damaging; 0.663
TCGA-95-7948-01, TCGA-HC-A76W-01	Lung adenocarcinoma; prostate adenocarcinoma	I256M	NA	No	Medium; 2.945	Deleterious; 0.01	Neutral; − 2.174	Possibly damaging; 0.471
MO_1111	Lung adenocarcinoma	A264S	NA	No	Medium; 3.225	Deleterious; 0.01	Deleterious; − 2.748	Probably damaging; 0.993
5-VS009-T2	Skin cancer, non-melanoma	D269N	NA	No	Low; 0.935	Tolerated; 0.79	Neutral; − 0.049	Probably damaging; 0.979
TCGA-A5-A0G1-01	Uterine serous carcinoma/uterine papillary serous carcinoma	D269Y	NA	No	Medium; 3.35	Deleterious; 0	Deleterious; − 5.728	Probably damaging; 1
TCGA-D3-A2JB-06	Cutaneous melanoma	G272C	NA	No	Medium; 3.27	Deleterious; 0	Deleterious; − 3.908	Probably damaging; 1
TCGA-D1-A103-01	Uterine endometrioid carcinoma	R273K	NA	No	Medium; 3.16	Deleterious; 0.01	Neutral; − 1.299	Probably damaging; 0.971
TCGA-AX-A05Z-01	Uterine endometrioid carcinoma	S280Y	NA	No	Medium; 2.015	Deleterious; 003	Deleterious; − 2.981	Possibly damaging; 0.865
TCGA-2J-AABP-01	Pancreatic adenocarcinoma	V293I	NA	No	Medium; 2.385	Tolerated; 0.19	Neutral; − 0.823	Possibly damaging; 0.862
TCGA-BA-A6DA-01	Head and neck squamous cell carcinoma	A296T	NA	No	Low; 1.005	Tolerated; 0.36	Neutral; − 1.315	Benign; 0.012
H104362	Hepatocellular adenoma	Q305L	NA	No	Neutral; − 0.35	Tolerated; 0.33	Neutral; − 0.224	Benign; 0.001
TCGA-D3-A8GI-06	Cutaneous melanoma	A311V	NA	No	High; 3.745	Deleterious; 0	Deleterious; − 3.619	Possibly damaging; 0.858
TCGA-FR-A729-06	Cutaneous melanoma	S317F	NA	No	High; 3.94	Deleterious; 0	Deleterious; − 5.837	Probably damaging; 0.998
TCGA-69-7980-01	Lung adenocarcinoma	L320F	NA	No	Medium; 2.125	Deleterious; 0	Deleterious; − 3.765	Probably damaging; 0.995
TCGA-AA-A01P-01	Colon adenocarcinoma	T324S	NA	No	Medium; 2.655	Tolerated; 0.14	Neutral; − 0.863	Benign; 0.187
TCGA-CU-A0YR-01	Bladder urothelial carcinoma	Q325P	NA	No	Neutral; 0.33	Tolerated; 1	Neutral; − 0.617	Benign; 0.003
TCGA-D7-6528-01	Tubular stomach adenocarcinoma	N330H	NA	No	Medium; 2.495	Tolerated; 0.07	Neutral; − 2.264	Probably damaging; 0.948
MO_1288	Breast invasive ductal carcinoma	T334R	NA	No	Medium; 3.205	Deleterious; 0.02	Neutral; − 0.94	Probably damaging; 0.93
MEL-IPI_Pat74-Tumor-SM-4DK2Z, Pat74, TCGA-AA-3977-01	Cutaneous melanoma, cutaneous melanoma, colon adenocarcinoma	G337E	NA	No	Low; 1.195	Tolerated; 0.43	Neutral; 0.189	Benign; 0.023
TCGA-VQ-A8P2-01	Mucinous stomach adenocarcinoma	N338D	NA	No	Neutral; − 2.73	Tolerated; 1	Neutral; 4.122	Benign; 0
coadread_dfci_2016_341	Colorectal adenocarcinoma	G352W	NA	No	Medium; 3.1	Deleterious; 0.01	Neutral; − 1.269	Probably damaging; 0.998
MEL-IPI_Pat11-Tumor-SM-4DK17, Pat11	Melanoma of unknown primary, cutaneous melanoma	D355N	NA	No	High; 3.985	Deleterious; 0	Deleterious; − 4.863	Probably damaging; 0.999
LUAD-LIP77	Lung adenocarcinoma	R357S	NA	No	High; 3.985	Deleterious; 0	Deleterious; − 5.956	Probably damaging; 0.998
TCGA-06-1801-01	Glioblastoma multiforme	I358F	NA	No	High; 3.64	Deleterious; 0	Deleterious; − 3.97	Probably damaging; 1
TCGA-AA-3947-01	Mucinous adenocarcinoma of the colon and rectum	V364A	NA	No	High; 3.545	Deleterious; 0	Deleterious; − 3.919	Possibly damaging; 0.502
WA48	Prostate adenocarcinoma	D367V	NA	No	Medium; 3.48	Deleterious; 0	Deleterious; − 6.741	Possibly damaging; 0.803
Pat_22_Post, Pat_22_Pre	Melanoma	D368N	NA	No	Low; 1.595	Tolerated; 0.07	Deleterious; − 3.187	Benign; 0.08
TCGA-EE-A20F-06	Cutaneous melanoma	M383I	NA	No	Medium; 2.87	Deleterious; 0.02	Neutral; − 2.431	Probably damaging; 0.987
Au8	Desmoplastic melanoma	R393G	NA	No	High; 3.925	Deleterious; 0	Deleterious; − 6.6	Probably damaging; 0.995
TCGA-56-7731-01	Lung squamous cell carcinoma	G395V	NA	No	High; 3.965	Deleterious; 0	Deleterious; − 8.923	Probably damaging; 1
585208	Small cell lung cancer	E398K	NA	No	Medium; 3.11	Tolerated; 0.1	Neutral; − 1.659	Probably damaging; 0.994
YULAN	Cutaneous melanoma	G399R	NA	No	High; 3.985	Deleterious; 0	Deleterious; − 7.699	Probably damaging; 0.999
CRUK0001-R1, CRUK0001-R2, CRUK0001-R3	Non-small cell lung cancer	A403V	NA	No	High; 4.015	Deleterious; 0	Deleterious; − 3.966	Probably damaging; 0.994
H072511	Hepatocellular adenoma	G405W	NA	No	High; 3.97	Deleterious; 0	Deleterious; − 7.932	Probably damaging; 1
MEL-JWCI-WGS-12	Cutaneous melanoma	E406K	NA	No	Medium; 3.18	Deleterious; 0.01	Deleterious; − 3.439	Probably damaging; 0.98
TCGA-GN-A26C-01	Cutaneous melanoma	S409L	NA	No	Medium; 3.455	Deleterious; 0	Deleterious; − 4.007	Probably damaging; 0.981
TCGA-BR-7707-01	Stomach adenocarcinoma	A412T	NA	No	Medium; 2.925	Deleterious; 0.01	Neutral; − 1.529	Possibly damaging; 0.471
TCGA-BS-A0UF-01	Uterine endometrioid carcinoma	K419T	NA	No	Medium; 3.33	Tolerated; 0.08	Deleterious; − 3.034	Possibly damaging; 0.74
CSCC-27-T	Cutaneous squamous cell carcinoma	P426L	NA	No	Medium; 2.145	Tolerated; 0.14	Deleterious; − 2.503	Benign; 0.319
LSD4744, LSD4744_T	Cutaneous melanoma	P426S	NA	No	Neutral; 0.235	Tolerated; 0.44	Neutral; − 0.568	Benign; 0.009
19424739	Renal non-clear cell carcinoma	D431G	NA	No	Medium; 2.275	Tolerated; 0.08	Deleterious; − 4.427	Benign; 0.317
TCGA-24-1564-01	Serous ovarian cancer	L450P	NA	No	High; 3.86	Deleterious; 0	Deleterious; − 6.343	Probably damaging; 0.998
TCGA-D7-6527-01	Papillary stomach adenocarcinoma	K458T	NA	No	Medium; 3.045	Tolerated; 0.06	Deleterious; − 2.553	Possibly damaging; 0.739
MO_1433	Lung adenocarcinoma	M462I	NA	No	Medium; 2.915	Tolerated; 0.18	Neutral; − 2.243	Possibly damaging; 0.739
coadread_dfci_2016_3498	Colorectal adenocarcinoma	I468T	NA	No	Low; 1.925	Tolerated; 0.25	Neutral; − 1.553	Possibly damaging; 0.695
TCGA-QK-A8Z8-01	Head and neck squamous cell carcinoma	W473L	NA	No	Medium; 2.285	Deleterious; 0.03	Deleterious; − 6.574	Probably damaging; 0.917
TCGA-92-7341-01	Lung squamous cell carcinoma	W477R	NA	No	High; 3.91	Deleterious; 0	Deleterious; − 13.293	Probably damaging; 1
MEL-IPI_Pat62-Tumor-SM-4DK2N, Pat62	Cutaneous melanoma, cutaneous melanoma	V488M	NA	No	Low; 1.46	Tolerated; 0.14	Neutral; − 0.928	Benign; 0.119
TCGA-Z2-A8RT-06	Cutaneous melanoma	E489K	NA	No	Medium; 3.075	Deleterious; 0.02	Neutral; − 1.459	Probably damaging; 0.992
TCGA-22-1016-01	Lung squamous cell carcinoma	V491L	NA	No	Neutral; 0.35	Tolerated; 0.27	Neutral; − 1.693	Benign; 0.024
TCGA-AA-A022-01	Colon adenocarcinoma	D494G	NA	No	Medium; 2.905	Deleterious; 0.01	Deleterious; − 3.416	Possibly damaging; 0.452
TCGA-ZJ-AB0H-01	Cervical squamous cell carcinoma	T496A	NA	No	Medium; 2.025	Tolerated; 0.24	Neutral; − 1.868	Benign; 0.005
TCGA-A5-A1OF-01	Uterine mixed endometrial carcinoma	R518M	NA	No	Medium; 3.18	Deleterious; 0.01	Deleterious; − 2.587	Probably damaging; 1
5-NB008-T1	Skin cancer, non-melanoma	G561R	NA	No	High; 4.02	Deleterious; 0	Deleterious; − 7.977	Probably damaging; 1
5-PT027-T1	Skin cancer, non-melanoma	P565L	NA	No	High; 3.515	Deleterious; 0	Deleterious; − 7.976	Probably damaging; 0.934
TCGA-A5-A0G2-01	Uterine serous carcinoma/uterine papillary serous carcinoma	K577N	NA	No	Low; 1.62	Tolerated; 0.2	Neutral; − 1.389	Benign; 0.137
PCNSL_4	Diffuse large B-cell lymphoma, NOS	M579T	NA	No	High; 3.53	Deleterious; 0	Deleterious; − 5.432	Probably damaging; 0.999
TCGA-06-6389-01	Glioblastoma multiforme	V581I	NA	No	Medium; 2.84	Deleterious; 0	Neutral; − 0.877	Benign; 0.343
TCGA-F1-6874-01	Intestinal type stomach adenocarcinoma	P590L	NA	No	High; 4.005	Deleterious; 0	Deleterious; − 8.236	Probably damaging; 0.958
TCGA-ND-A4WC-01	Uterine carcinosarcoma/uterine malignant mixed Mullerian tumor	D597E	NA	No	Neutral; − 1.745	Tolerated; 1	Neutral; 1.223	Benign; 0
FR9547	Lung adenocarcinoma	N599K	NA	No	High; 3.615	Deleterious; 0.01	Deleterious; − 3.926	Probably damaging; 0.998
TCGA-A5-A2K5-01	Uterine endometrioid carcinoma	K600N	NA	No	Low; 1.615	Tolerated; 0.36	Neutral; 0.683	Possibly damaging; 0.451
DFCI-CLL139-Tumor	Chronic lymphocytic leukemia/small lymphocytic lymphoma	N601I	NA	No	Medium; 3.415	Deleterious; 0	Deleterious; − 4.964	Possibly damaging; 0.813
TCGA-AA-3984-01	Colon adenocarcinoma	D609N	NA	No	Low; 0.91	Tolerated; 0.46	Neutral; − 0.265	Benign; 0.003
TCGA-AU-6004-01	Colon adenocarcinoma	Y613H	NA	No	Medium; 2.685	Deleterious; 0.05	Neutral; − 1.59	Probably damaging; 0.965
SJERG016_D_WES	Acute lymphoid leukemia	D615Y	NA	No	Medium; 2.3	Deleterious; 0	Deleterious; − 2.848	Benign; 0.009
TCGA-EK-A2RN-01	Cervical squamous cell carcinoma	I618M	NA	No	Medium; 2.945	Deleterious; 0	Neutral; − 0.777	Probably damaging; 0.985
TCGA-AA-A00N-01	Mucinous adenocarcinoma of the colon and rectum	K625T	NA	No	Medium; 2.89	Deleterious; 0.02	Neutral; − 1.37	Possibly damaging; 0.632
TCGA-AA-A010-01	Colon adenocarcinoma	L628F	NA	No	Medium; 2.53	Tolerated; 0.05	Neutral; − 0.944	Benign; 0.086
TCGA-BR-4201-01	Stomach adenocarcinoma	R644Q	NA	No	Low; 1.19	Tolerated; 0.42	Neutral; − 0.146	Benign; 0.014
MO_1146	Cutaneous melanoma	E667K	NA	No	Medium; 2.3	Tolerated low confidence; 0.35	Neutral; − 0.312	Benign; 0.045
TCGA-55-8506-01	Lung adenocarcinoma	V670L	NA	No	Medium; 3.085	Deleterious low confidence; 0.01	Neutral; − 0.314	Benign; 0.303
MSKCC-0411_R	Bladder urothelial carcinoma	V672A	NA	No	Medium; 2.59	Tolerated low confidence; 0.07	Neutral; − 0.888	Benign; 0.045
CRUK0027-R2	Non-small cell lung cancer	K676E	NA	No	Medium; 2.77	Deleterious low confidence; 0.03	Neutral; − 0.676	Benign; 0.275
TCGA-AG-3892-01, TCGA-F5-6814-01	Rectal adenocarcinoma, rectal adenocarcinoma	F683L	NA	No	Medium; 2.8	Tolerated low confidence; 0.18	Neutral; − 1.182	Benign; 0.026
TCGA-L5-A8NQ-01	Esophageal squamous cell carcinoma	S692F	NA	No	Medium; 2.855	Deleterious low confidence; 0	Neutral; − 1.523	Possibly damaging; 0.778
TCGA-73-4658-01	Lung adenocarcinoma	D693N	NA	No	Low; 1.705	Tolerated low confidence; 0.15	Neutral; − 0.592	Benign; 0.003
TCGA-CR-6484-01	Head and neck squamous cell carcinoma	I694M	NA	No	Medium; 2.6	Deleterious low confidence; 0.02	Neutral; − 0.608	Benign; 0.02
5-VS045-T1	Skin cancer, non-melanoma	E701K	NA	No	Medium; 2.695	Deleterious low confidence; 0.02	Neutral; − 1.068	Benign; 0.245
coadread_dfci_2016_102, TCGA-RD-A8NB-01	Colorectal adenocarcinoma, diffuse type stomach adenocarcinoma	R708Q	NA	No	Medium; 3.195	Deleterious low confidence; 0	Neutral; − 1.374	Probably damaging; 0.935
DLBCL-RICOVER_1150	Activated B-cell type	D713N	NA	No	Medium; 1.995	Deleterious low confidence; 0.04	Neutral; − 0.721	Possibly damaging; 0.451
coadread_dfci_2016_3064	Colorectal adenocarcinoma	R716H	NA	No	Medium; 2.635	Tolerated low confidence; 0.08	Neutral; − 0.886	Benign; 0.277
TCGA-HZ-7922-01	Pancreatic adenocarcinoma	R716C	NA	No	Medium; 2.635	Deleterious low confidence; 0.01	Neutral; − 1.638	Possibly damaging; 0.513
OS-47-SJ	Osteosarcoma	N720S	NA	No	Medium; 2.245	Tolerated low confidence; 0.11	Neutral; − 0.939	Benign; 0.014
CHC2128T	Hepatocellular carcinoma	P737H	NA	No	Medium; 3.02	Deleterious low confidence; 0	Neutral; − 1.691	Possibly damaging; 0.778
CSCC-31-T	Cutaneous squamous cell carcinoma	P737L	NA	No	Medium; 3.02	Deleterious low confidence; 0	Neutral; − 2.015	Possibly damaging; 0.478
NCH-CA-3	Colorectal adenocarcinoma	V748F	NA	No	Medium; 3.135	Deleterious low confidence; 0.04	Neutral; − 1.816	Probably damaging; 0.937
TCGA-AJ-A3BH-01	Uterine endometrioid carcinoma	L760M	NA	No	Medium; 2.845	Deleterious low confidence; 0	Neutral; − 0.689	Probably damaging; 0.999
AMPAC_719	Ampullary carcinoma	I761T	NA	No	Medium; 2.965	Deleterious low confidence; 0	Neutral; − 1.835	Possibly damaging; 0.662
TCGA-4A-A93Y-01	Papillary renal cell carcinoma	F762L	NA	No	Low; 1.395	Deleterious low confidence; 0.04	Neutral; 0.066	Benign; 0.005
5-PT001-T1	Skin cancer, non-melanoma	G764R	NA	No	Medium; 3.205	Deleterious low confidence; 0	Deleterious; − 3.323	Possibly damaging; 1
TCGA-19-1390-01	Glioblastoma multiforme	R766K	NA	No	Low; 1.3	Tolerated low confidence; 0.21	Neutral; − 0.688	Benign; 0.024
LUAD_E00522	Lung adenocarcinoma	R768L	NA	No	Medium; 3.13	Deleterious low confidence; 0	Neutral; − 2.135	Probably damaging; 0.934
TCGA-E6-A1LX-01	Uterine endometrioid carcinoma	R768W	NA	No	Medium; 3.13	Deleterious low confidence; 0	Deleterious; − 2.822	Probably damaging; 0.995
TCGA-BS-A0UJ-01	Uterine endometrioid carcinoma	R775I	NA	No	Low; 1.79	Tolerated low confidence; 0.13	Neutral; − 0.585	Benign; 0.001
TCGA-ER-A1A1-06	Cutaneous melanoma	P780S	NA	No	Medium; 2.44	Deleterious low confidence; 0	Neutral; − 1.843	Benign; 0.253
SC_9081-TM, YURED	Prostate adenocarcinoma, cutaneous melanoma	D785N	NA	No	Low; 1.81	Tolerated low confidence; 0.17	Neutral; − 0.416	Benign; 0.011
Pt1	Melanoma	G789R	NA	No	Medium; 3.12	Deleterious low confidence; 0.02	Neutral; − 1.328	Probably damaging; 0.937
TCGA-55-8205-01	Lung adenocarcinoma	T798P	NA	No	Low; 1.245	Tolerated low confidence; 0.14	Neutral; − 1.163	Benign; 0.06
TCGA-VS-A958-01	Cervical squamous cell carcinoma	T803I	NA	No	Medium; 3.025	Deleterious low confidence; 0	Neutral; − 1.249	Benign; 0.388
587376	Colorectal adenocarcinoma	S47C, P284S	NA, NA	No, No	High; 3.62, Medium; 3.295	Deleterious; 0, Deleterious; 0	Deleterious; − 4.169, Deleterious; − 7.65	Probably damaging; 0.987, Possibly damaging; 0.742
5-VS022-T1	Skin cancer, non-melanoma	E145K, E639K	NA, NA	No, No	High; 3.63, Medium; 2.815	Deleterious; 0.01, Deleterious; 0	Deleterious; − 3.486, neutral; − 1.422	Possibly damaging; 0.865, Probably damaging; 0.983
coadread_dfci_2016_116	Colorectal adenocarcinoma	W302G, F400L	NA, NA	No, No	High; 3.62, High; 4.015	Deleterious; 0.01, Deleterious; 0	Deleterious; − 10.299, Deleterious; − 5.949	Probably damaging; 0.996, Probably damaging; 0.988
Pat_41_Post	Melanoma	T593I, P729S	NA, NA	No, No	Medium; 2.87, Medium; 2.1	Tolerated; 0.13, Deleterious low confidence; 0.01	Neutral; − 2.322, Neutral; − 2.162	Benign; 0.095, Possibly damaging; 0.548
TCGA-AP-A1E0-01	Uterine endometrioid carcinoma	H195Y, F683L	NA, NA	No, No	Neutral; 0.69, Medium; 2.8	Deleterious; 0, Tolerated low confidence; 0.18	Neutral; − 1.761, Neutral; − 1.182	Benign; 0.33, Benign; 0.026
TCGA-AX-A0J0-01	Uterine endometrioid carcinoma	K131Q, F683L	NA, NA	No, No	Medium; 2.665, Medium; 2.8	Tolerated; 0.26, Tolerated low confidence; 0.18	Neutral; − 1.731, Neutral; − 1.182	Benign; 0.04, Benign; 0.026
TCGA-B5-A1MR-01	Uterine endometrioid carcinoma	L120I, V658L	NA, NA	No, No	Medium; 2.16, Medium; 2.075	Deleterious; 0.01, Tolerated low confidence; 0.21	Neutral; − 1.397, Neutral; − 0.612	Benign; 0.069, Benign; 0.003
TCGA-EE-A183-06	Cutaneous melanoma	S280Y, Q598H	NA, NA	No, No	Medium; 2.015, High; 3.57	Deleterious; 0.03, Deleterious; 0	Deleterious; − 2.981, Neutral; − 2.063	Possibly damaging; 0.865, Possibly damaging; 0.723
TCGA-FI-A2D5-01	Uterine endometrioid carcinoma	D427N, A576T	NA, NA	No, No	Low; 1.435, Neutral; − 0.405	Tolerated; 0.28, Tolerated; 0.59	Neutral; − 0.822, Neutral; 0.153	Benign; 0.006, Benign; 0.003
TCGA-AP-A1DK-01	Uterine endometrioid carcinoma	W48L, N437H	NA, NA	No, No	High; 4, High; 3.905	Deleterious; 0, Deleterious; 0	Deleterious; − 12.017, Deleterious; − 4.971	Probably damaging; 0.999, probably damaging; 1
TCGA-B5-A11H-01	Uterine endometrioid carcinoma	P336S, K26N	NA, NA	No, No	Medium; 3.265, Medium; 2.19	Deleterious; 0, Tolerated; 0.09	Deleterious; − 7.779, Neutral; − 1.899	Possibly damaging; 0.666, Benign; 0.013
TCGA-EO-A22R-01	Uterine endometrioid carcinoma	N578S, Y497C	NA, NA	No, No	Medium; 2.56, Medium; 3.095	Tolerated; 0.35, Tolerated; 0.07	Neutral; − 1.528, Deleterious; − 3.744	Benign; 0.003, Probably damaging; 0.997
TCGA-06-5416-01	Glioblastoma multiforme	P178S, E182D, D427N	NA, NA, NA	No, No, No	Medium; 2.12, Low; 1.83, Low; 1.435	Tolerated; 0.13, Tolerated; 0.27, tolerated; 0.28	Deleterious; − 4.3, Neutral; − 1.291, neutral; − 0.822	Benign; 0.301, Benign; 0.003, benign; 0.006
TCGA-AG-A002-01	Rectal adenocarcinoma	R169I, H195Y, N394H	NA, NA, NA	No, No, No	High; 3.73, Neutral; 0.69, Medium; 2.705	Deleterious; 0, Deleterious; 0, deleterious; 0.01	Deleterious; − 7.091, Neutral; − 1.761, Neutral; − 1.654	Probably damaging; 1, Benign; 0.33, Probably damaging; 0.853
TCGA-AP-A056-01	Uterine endometrioid carcinoma	N194K, R306I, E479D	NA, NA, NA	No, No, No	High; 3.735, Medium; 3.335, Low; 1.475	Deleterious; 0.01, Deleterious; 0, Tolerated; 0.15	Deleterious; − 5.181, Deleterious; − 5.952, Neutral; − 1.331	Probably damaging; 0.961, Possibly damaging; 0.678, Possibly damaging; 0.542
TCGA-B5-A11E-01	Uterine endometrioid carcinoma	S128I, R169I, S602Y	NA, NA, NA	No, No, No	Medium; 3.42, High; 3.73, Medium; 2.11	Deleterious; 0, Deleterious; 0, Deleterious; 0.02	Deleterious; − 5.331, Deleterious; − 7.091, Neutral; − 1.832	Probably damaging; 0.963, Probably damaging; 1, Benign; 0.039
TCGA-BS-A0UV-01	Uterine endometrioid carcinoma	M82T, F314L, K600N	NA, NA, NA	No, No, No	Neutral; − 1.78, Medium; 3.43, Low; 1.615	Tolerated; 0.63, Deleterious; 0.01, Tolerated; 0.36	Neutral; 0.821, Deleterious; − 5.938, Neutral; 0.683	Benign; 0, Probably damaging; 0.987, Possibly damaging; 0.451
TCGA-D1-A17Q-01	Uterine endometrioid carcinoma	E375D, K577N, R768W	NA, NA, NA	No, No, No	High; 3.99, Low; 1.62, Medium; 3.13	Deleterious; 0, Tolerated; 0.2, Deleterious low confidence; 0	Deleterious; − 2.974, Neutral; − 1.389, Deleterious; − 2.822	Probably damaging; 1, Benign; 0.137, Probably damaging; 0.995
TCGA-D3-A2JP-06	Cutaneous melanoma	Q18K, G268C, W610L	NA, NA, NA	No, No, No	Medium; 3.055, High; 3.985, Medium; 3.155	Tolerated; 0.06, Deleterious; 0, Deleterious; 0.01	Neutral; − 0.98, Deleterious; − 7.947, Deleterious; − 6.794	Possibly damaging; 0.742, Probably damaging; 1, Benign; 0.344
TCGA-DU-6392-01	Astrocytoma	A25V, A396T, I679N	NA, NA, NA	No, No, No	Medium; 3.41, Medium; 3.45, Medium; 2.645	Deleterious; 0.01, Deleterious; 0.01, Deleterious low confidence; 0	Deleterious; − 3.317, Deleterious; − 3.639, Neutral; − 2.435	Possibly damaging; 0.545, Probably damaging; 0.999, Possibly damaging; 0.714
TCGA-EO-A22U-01	Uterine endometrioid carcinoma	R393I, E571G, R768W	NA, NA, NA	No, No, No	High; 3.925, High; 3.53, Medium; 3.13	Deleterious; 0, Deleterious; 0, Deleterious low confidence; 0	Deleterious; − 7.592, Deleterious; − 5.12, Deleterious; − 2.822	Probably damaging; 0.994, Possibly damaging; 0.89, Probably damaging; 0.995

The SIFT impact scores predicted 80 single mutants E22D, F28L, E37K, S44L, F72C, S109L, R115W, L116F, G147V, L162F, H195Y, Y202H, R204I, G205V, R219P, R219H, G220C, E232K, I256M, A264S, D269Y, G272C, R273K, S280Y, A311V, S317F, L320F, T334R, G352W, D355N, R357S, I358F, V364A, D367V, M383I, R393G, G395V, G399R, A403V, G405W, E406K, S409L, A412T, L450P, W473L, W477R, E489K, D494G, R518M, G561R, P565L, M579T, V581I, P590L, N599K, N601I, Y613H, D615Y, I618M, K625T, V670L, K676E, S692F, I694M, E701K, R708Q, D713N, R716C, P737H, P737L, V748F, L760M, I761T, F762L, G764R, R768L, R768W, P780S, G789R and T803I and 9 multi-mutants S47C/P284S, E145K/E639K, W302G/F400L, S280Y/Q598H, W48L/N437H, R169I/H195Y/N394H, S128I/R169I/S602Y, A25V/A396T/I679N and R393I/E571G/R768W considered to have a deleterious impact on the respective protein’s functionality, the score ranging from 0 to 0.05. Twenty mutants out of these, however, had a low confidence score, including V670L, K676E, S692F, I694M, E701K, R708Q, D713N, R716C, P737H, P737L, V748F, L760M, I761T, F762L, G764R, R768L, R768W, P780S, G789R and T803I. The other 54 single mutants L8F, T20I, H34N, E35K, L39M, V59D, L73S, A99S, R115Q, P138S, E182D, E189K, D206Y, G211W, V212I, D213G, A242T, D269N, V293I, A296T, Q305L, T324S, Q325P, N330H, G337E, N338D, D368N, E398K, K419T, P426L, P426S, D431G, K458T, M462I, I468T, V488M, V491L, T496A, K577N, D597E, K600N, D609N, L628F, R644Q, E667K, V672A, F683L, D693N, R716H, N720S, R766K, R775I, D785N and T798P, and four multi-mutants K131Q/F683L, D427N/A576T, N578S/Y497C and P178S/E182D/D427N exhibited prediction scores with tolerated impact on protein functionality.

Additionally, PROVEAN scores displayed 47 single mutants F28L, F72C, S109L, R115W, G147V, L162F, Y202H, R204I, A242T, A264S, D269Y, G272C, S280Y, A311V, S317F, L320F, D355N, R357S, I358F, V364A, D367V, D368N, R393G, G395V, G399R, A403V, G405W, E406K, S409L, K419T, P426L, D431G, L450P, K458T, W473L, W477R, D494G, R518M, G561R, P565L, M579T, P590L, N599K, N601I, D615Y. G764R and R768W and 4 multi-mutants S47C/P284S, W302G/F400L, W48L/N437H, and R393I/E571G/R768W, to have a deleterious effect on the proteins. 87 single mutants and five multi-mutants, had a neutral impact score, inclusive of L8F, T20I, E22D, H34N, E35K, E37K, L39M, S44L, V59D, L73S, A99S, R115Q, L116F, P138S, E182D, E189K, H195Y, G205V, D206Y, G211W, V212I, D213G, R219P, R219H, G220C, E232K, I256M, D269N, R273K, V293I, A296T, Q305L, T324S, Q325P, N330H, T334R, G337E, N338D, G352W, M383I, E398K, A412T, P426S, M462I, I468T, V488M, E489K, V491L, T496A, K577N, V581I, D597E, K600N, D609N, Y613H, I618M, K625T, L628F, R644Q, E667K, V670L, V672A, K676E, F683L, S692F, D693N, I694M, E701K, R708Q, D713N, R716H, R716C, N720S, P737H, P737L, V748F, L760M, I761T, F762L, R766K, R768L, R775I, P780S, D785N, G789R, T798P, T803I, T593I/P729S, H195Y/F683L, K131Q/F683L, L120I/V658L and D427N/A576T. 12 multi-mutants E145K/E639K, S280Y/Q598H, P336S/K26N, N578S/Y497C, P178S/E182D/D427N, R169I/H195Y/N394H, N194K/R306I/E479D, S128I/R169I/S602Y, M82T/F314L/K600N, E375D/K577N/R768W, Q18K/G268C/W610L and A25V/A396T/I679N, however, could not be categorized into an overall deleterious or neutral section since their single mutants revealed both types of functional effects predicted on the respective proteins.

Furthermore, PolyPhen-2 prediction scores categorized 50 single mutants F28L, S44L, F72C, G147V, L162F, Y202H, R204I, D206Y, R219P, R219H, A264S, D269N, D269Y, G272C, R273K, S317F, L320F, N330H, T334R, G352W, D355N, R357S, I358F, M383I, R393G, G395V, E398K, G399R, A403V, G405W, E406K, S409L, L450P, W473L, W477R, E489K, R518M, G561R, P565L, M579T, P590L, N599K, Y613H, I618M, R708Q, V748F, L760M, R768L, R768W and G789R, and two multi-mutants W302G/F400L and W48L/N437H, with probably damaging impact on the proteins’ functionality, hence, marking these mutants under the highest risk. The impact scores predicted for these mutants fall between 0.9 to 1, indicating their possible deleterious effects with high confidence. Additionally, 30 single mutants E22D, E37K, L39M, S109L, R115W, L116F, G205V, A242T, I256M, S280Y, V293I, A311V, V364A, D367V, A412T, K419T, K458T, M462I, I468T, D494G, K600N, N601I, K625T, S692F, D713N, R716C, P737H, P737L, I761T, and G764R and one multi-mutant S280Y/Q598H had possibly damaging effects, ranking them at a lower risk compared to probably damaging mutants, based on the score bracket of 0.4 to 0.9. The rest of the 54 single mutants, L8F, T20I, H34N, E35K, V59D, L73S, A99S, R115Q, P138S, E182D, E189K, H195Y, G211W, V212I, D213G, G220C, E232K, A296T, Q305L, T324S, Q325P, G337E, N338D, D368N, P426L, P426S, D431G, V488M, V491L, T496A, K577N, V581I, D597E, D609N, D615Y, L628F, R644Q, 667K, V670L, V672A, K676E, F683L, D693N, I694M, E701K, R716H, N720S, F762L, R766K, R775I, P780S, D785N, T798P and T803I, and five multi-mutants H195Y/F683L, K131Q/F683L, L120I/V658L, D427N/A576T, N578S/Y497C and P178S/E182D/D427N exhibited scores between 0 to 0.4, indicating benign outcomes over protein functionalities. On the contrary, 13 multi-mutants T593I/P729S, N578S/Y497C, M82T/F314L/K600N, P336S/K26N, S47C/P284S, E145K/E639K, R169I/H195Y/N394H, N194K/R306I/E479D, S128I/R169I/S602Y, E375D/K577N/R768W, Q18K/G268C/W610L, A25V/A396T/I679N and R393I/E571G/R768W had different impact scores of their respective single mutants in each set, hence making them difficult to categorize in one particular section, similar to the observation of PROVEAN scores.

#### Impact of cell line mutations in cancer association

Based on the Mutation Assessor prediction scores, three single mutants, P612L, V184A, and Y252C, had high impact, scoring between 3.6 and 4 (Table 6). Only two single mutants, S218N and A782V, were observed with low impact scores, while 15 mutants, E668K, P426L, A413V, L100V, N322I, K416N, P253T, Y649C, T276K, Q472P, E457K, W635L, T334A, A25V and F314L/Y510H had a medium impact score and S5F mutant had a neutral effect. Among the five multi-mutants, E145K/E495K/I233S and A386T/F314I displayed a combination of high and medium impact on the functionality of the proteins, while the other two mutants F603C/K619N and L664I/D382Y did not reveal any significant impact on protein function.

**Table 6:** Impact of cell line mutations in cancer.

Sample ID	Cancer type	Mutation	OncoKB	Cancer hotspot	Mutation Assessor (impact and score)	SIFT (impact and score)	PROVEAN (prediction; score)	PolyPhen-2 (impact and score)
WILD TYPE	–	–	–	–	–	–	–	–
JSC1_HAEMATOPOIETIC_AND_LYMPHOID_TISSUE	Mixed cancer types	S5F	NA	No	Neutral; 0.125	Tolerated; 0.86	Neutral; − 0.501	Benign; 0
ISHIKAWAHERAKLIO02ER_ENDOMETRIUM	Mixed cancer types	A25V	NA	No	Medium; 3.41	Deleterious; 0.01	Deleterious; − 3.317	Possibly damaging; 0.545
OSRC2_KIDNEY	Mixed cancer types	L100V	NA	No	Medium; 2.33	Tolerated; 0.14	Neutral; − 1.149	Possibly damaging; 0.501
JHOS2_OVARY	Mixed cancer types	V184A	NA	No	High; 3.605	Deleterious; 0.04	Deleterious; − 3.705	Probably damaging; 0.981
HEC59_ENDOMETRIUM	Mixed cancer types	S218N	NA	No	Low; 0.905	Tolerated; 0.2	Neutral; − 0.488	Benign; 0.001
A172_CENTRAL_NERVOUS_SYSTEM	Mixed cancer types	Y252C	NA	No	High; 3.975	Deleterious; 0	Deleterious; − 7.992	Probably damaging; 0.998
NCIH513_PLEURA	Mixed cancer types	P253T	NA	No	Medium; 2.875	Deleterious; 0.01	Neutral; − 1.573	Benign; 0.293
SUDHL10_HAEMATOPOIETIC_AND_LYMPHOID_TISSUE	Mixed cancer types	T276K	NA	No	Medium; 3.13	Deleterious; 0.04	Deleterious; − 2.554	Possibly damaging; 0.9
MCC26_SKIN	Mixed cancer types	N322I	NA	No	Medium; 2.34	Deleterious; 0.02	Neutral; − 2.479	Benign; 0.389
CAL54_KIDNEY	Mixed cancer types	T334A	NA	No	Medium; 3.205	Tolerated; 0.26	Neutral; − 1.163	Benign; 0.176
LS123_LARGE_INTESTINE	Mixed cancer types	A413V	NA	No	Medium; 2.28	Deleterious; 0.03	Deleterious; − 3.413	Possibly damaging; 0.471
EN_ENDOMETRIUM	Mixed cancer types	K416N	NA	No	Medium; 2.365	Deleterious; 0.05	Deleterious; − 3.604	Benign; 0.03
LU165_LUNG	Mixed cancer types	P426L	NA	No	Medium; 2.145	Tolerated; 0.14	Deleterious; − 2.503	Benign; 0.319
GMEL_SKIN	Mixed cancer types	E457K	NA	No	Medium; 3.195	Deleterious; 0	Deleterious; − 3.404	Probably damaging; 0.999
LS411N_LARGE_INTESTINE	Mixed cancer types	Q472P	NA	No	Medium; 3.16	Tolerated; 0.05	Deleterious; − 3.07	Possibly damaging; 0.609
TMK1_STOMACH	Mixed cancer types	P612L	NA	No	High; 3.6	Deleterious; 0.01	Deleterious; − 7.057	Probably damaging; 0.918
CORL32_LUNG	Mixed cancer types	W635L	NA	No	Medium; 3.195	Deleterious; 0	Deleterious; − 4.371	Probably damaging; 0.999
HCT_15, HCT15_LARGE_INTESTINE	Colorectal adenocarcinoma, mixed cancer types	Y649C	NA	No	Medium; 2.955	Deleterious; 0	Deleterious; − 3.453	Probably damaging; 0.966
PECAPJ15_UPPER_AERODIGESTIVE_TRACT	Mixed cancer types	E668K	NA	No	Medium; 1.995	Tolerated low confidence; 0.11	Neutral; − 0.547	Benign; 0.005
MESSA_SOFT_TISSUE	Mixed cancer types	A782V	NA	No	Low; 1.52	Tolerated low confidence; 0.27	Neutral; − 0.059	Benign; 0.001
CW2_LARGE_INTESTINE	Mixed cancer types	A386T, F314I	NA, NA	No, No	Medium; 3.16, High; 3.975	Deleterious; 0.04, Deleterious; 0.01	Neutral; − 1.237, Deleterious; − 5.938	Possibly damaging; 0.477, Probably damaging; 0.996
HCC_2998; HCC2998_LARGE_INTESTINE	Colorectal adenocarcinoma; mixed cancer types	F603C, K619N	NA, NA	No, No	Low; 1.905, Medium; 2.965	Tolerated; 0.16, Deleterious; 0.03	Neutral; − 1.272, Neutral; − 1.564	Benign; 0.058, Possibly damaging; 0.821
HEC251_ENDOMETRIUM	Mixed cancer types	F314L, Y510H	NA, NA	No, No	Medium; 3.43, Medium; 2.75	Deleterious; 0.01, Deleterious; 0.01	Deleterious; − 5.938, Deleterious; − 3.071	Probably damaging; 0.987, Probably damaging; 0.979
JHUEM7_ENDOMETRIUM	Mixed cancer types	L664I, D382Y	NA, NA	No, No	Medium; 1.95, Neutral; 0.595	Tolerated low confidence; 0.18, Tolerated; 0.4	Neutral; − 0.166, Neutral; 1.835	Benign; 0.06, Benign; 0.201
MCC13_SKIN	Mixed cancer types	E145K, E495K, I233S	NA, NA, NA	No, No, No	High; 3.63, High; 3.535, Medium; 3.405	Deleterious; 0.01, Deleterious; 0.01, Deleterious; 0	Deleterious; − 3.486, Deleterious; − 3.118, Deleterious; − 4.402	Possibly damaging; 0.865, Probably damaging; 0.992, Probably damaging; 0.986

SIFT scores predicted 15 mutants, Y252C, E457K, W635L, Y649C, K416N, A25V, P253T, P612L, N322I, A413V, V184A, T276K, A386T/F314I, F314L/Y510H, and E145K/E495K/I233S to possess a deleterious impact on the function of the respective proteins. The impact scores for deleterious impact ranged from 0 to 0.05. The other nine mutants, E668K, A782V, Q472P, L100V, P426L, S218N, T334A, S5F and L664I/D382Y had a tolerated impact, wherein E668K and A782V displaying low confidence scores. However, F603C/K619N multi-mutant had both tolerated and deleterious impact.

Likewise, 14 mutants, P426L, T276K, Q472P, A25V, E457K, A413V, Y649C, K416N, V184A, W635L, P612L, Y252C, F314L/Y510H and E145K/E495K/I233S were recognized with a deleterious impact based on the PROVEAN scoring. Ten other mutants, A782V, S218N, S5F, E668K, L100V, T334A, P253T, N322I, F603C/K619N, and L664I/D382Y had a neutral impact on protein functioning. A386T/F314I suggested a deleterious impact for F314I and neutral for A386T.

The PolyPhen-2 prediction scores identified mutants scoring between 0.9 to 1 had a probably damaging effect with the higher confidence level, including mutants P612L, V184A, Y252C, E457K, W635L, Y649C, and F314L/Y510H. Mutants scored between 0.4 to 0.9 had a possibly damaging impact on protein functionality, which were A413V, L100V, A25V, Q472P, T276K single mutants. On the other hand, mutants with a score of less than 0.4 were considered benign by this tool, namely S5F, S218N, A782V, E668K, K416N, T334A, P253T, P426L, N322I, and L664I/D382Y. A386T/F314I and E145K/E495K/I233S fall next in a hierarchy with both possible and probable damaging scores, while F603C/K619N had only K619N with possible damage-causing impact.

### Mutations affect the binding affinity

#### Experimental known ACE2 mutations disrupt the binding affinity with SARS-CoV and SARS-CoV-2

Variations in the functional sites of ACE2 can affect the interaction with SARS-CoV and SARS-CoV-2. The binding impact of experimental validated ACE2 mutants with wild-type SARS-CoV binding is illustrated in Table 7. The ACE2 single mutants, K31D, Y41A, K68D, K353H, K353A, K353D, D355A, R357A, M383A, P389A, R393A, R559S, inhibits the interaction with wild-type SARS-CoV. Similarly, the ACE2 multiple mutants Q24K/A25A/K26E, M82N/Y83F/P84S, and S425P/P426S/D427N inhibit the interaction with wild-type SARS-CoV. In contrast, no interaction inhibition was reported between SARS-CoV and ACE2 single mutants E37A, D38A, E110P, E160R, R192D, R219D, H239Q, K309D, E312A, T324A, D350A, L359K, L359A, and F603T. Likewise, ACE2 multiple mutants P135S/D136M, N338D/V339D/Q340R, and K465Q/G466D/E467K also demonstrated the binding remains unaffected with SARS-CoV.

**Table 7:** Comparison of experimental known mutants’ impact with docking between ACE2 mutants and SARS-CoV, and SARS-CoV-2 respectively.

Mutants	Experimentally validated binding effect of mutants with SARS-CoV	ZDOCK (score)		ClusPro (score)		HDOCK (score; ligand rmsd (Å))		PatchDock (score)		InterEvDock2 (score)		SOAP-PP (score)		FRODOCK2 (score)	
		SARS-CoV	SARS-CoV-2	SARS-CoV	SARS-CoV-2	SARS-CoV	SARS-CoV-2	SARS-CoV	SARS-CoV-2	SARS-CoV	SARS-CoV-2	SARS-CoV	SARS-CoV-2	SARS-CoV	SARS-CoV-2
Wild	Binds	1914.103	1610.91	− 961.7	− 911.6	− 263.89; 1.21	− 279.45; 0.53	15,788	17,820	39.33	38.23	− 33,829.06	− 34,179.68	2230.49	2256.97
K31D	Inhibition	1913.964	1610.801	− 961.6	− 912.4	− 256.18; 60.98	− 309.45; 82.28	16,048	17,048	41.19	38.23	− 33,772.5	− 34,168.56	2230.56	2248.57
E37A	No inhibition	1925.772	1622.068	− 962	− 913	− 254.77; 0.68	− 281.03; 99.16	16,402	16,982	36.78	38.62	− 33,813.66	− 34,040.86	2235.21	2212.18
D38A	No inhibition	1940.72	1622.226	− 962	− 913	− 266.37; 99.72	− 295.14; 0.49	17,604	17,014	41.19	38.51	− 33,599.38	− 34,076.01	2231.25	2204.7
Y41A	Inhibition	1914.075	1610.882	− 960.4	− 911.5	− 251.72; 91.50	− 289.24; 102.74	18,568	18,524	36.78	38.62	− 33,844.8	− 34,060.11	2239.67	2188.15
K68D	Inhibition	1915.319	1612.938	− 960.4	− 911.5	− 277.13; 91.15	− 277.53; 0.45	18,008	17,452	41.19	38.23	− 33,576.37	− 34,171.04	2229.26	2261.9
E110P	No inhibition	1934.682	1615.521	− 963.1	− 912.6	− 271.88; 0.48	− 281.26; 0.46	17,428	16,454	41.19	38.23	− 33,796.22	− 34,179.79	2229.83	2216.52
E160R	No inhibition	1946.638	1623.785	− 964.6	− 912.8	− 260.92; 1.25	− 272.52; 99.93	16,874	16,410	37.65	38.23	− 33,550.85	− 34,076.85	2222.34	2241.78
R192D	No inhibition	1917.07	1609.263	− 960.6	− 912	− 291.31; 80.13	− 300.36; 0.43	17,598	17,104	41.19	37.69	− 33,593.66	− 34,193.02	2224.44	2260.41
R219D	No inhibition	1768.002	1650.156	− 963.1	− 902.9	− 301.55; 97.40	− 288.20; 0.55	17,178	16,640	36.78	37.89	− 33,938.72	− 34,146.68	2199.31	2184.6
H239Q	No inhibition	1926.197	1608.987	− 960.7	− 911.6	− 265.19; 96.68	− 300.75; 0.52	16,488	17,526	41.19	38.23	− 33,788.76	− 34,114.52	2229.56	2278.52
K309D	No inhibition	1913.786	1785.692	− 962	− 912	− 280.96; 0.57	− 291.27; 0.62	18,010	16,212	41.19	38.51	− 33,578.4	− 34,176.94	2227.95	2266.2
E312A	No inhibition	1927.113	1609.776	− 962	− 913	− 262.45; 0.47	− 284.61; 0.86	17,524	16,928	37.65	38.51	− 33,862.26	− 34,116.02	2234.86	2249.2
T324A	No inhibition	1914.041	1610.859	− 960.4	− 911.6	− 265.78; 33.53	− 283.64; 0.71	16,042	16,480	37.76	38.23	− 33,805.22	− 34,161.33	2229.7	2245.57
D350A	No inhibition	1915.426	1612.916	− 961.6	− 911.5	− 267.17; 111.39	− 275.14; 0.61	17,370	17,534	41.19	38.23	− 33,633.03	− 34,096.8	2227.8	2270.56
K353H	Inhibition	1940.69	1622.135	− 962	− 842.5	− 275.14; 96.20	− 284.06; 0.51	16,518	17,280	41.19	38.23	− 33,547.17	− 34,133.94	2230.62	2279.49
K353A	Inhibition	1944.888	1625.737	− 964.6	− 842.5	− 265.85; 96.40	− 282.67; 0.56	17,608	17,194	37.65	38.62	− 33,786.33	− 34,012.46	2193.4	2203.86
K353D	Inhibition	1915.269	1612.912	− 961.6	− 911.5	− 277.94; 79.96	− 298.02; 84.35	18,002	17,458	41.19	38.23	− 33,640.15	− 34,190.89	2229.01	2256.33
D355A	Inhibition	1932.896	1627.444	− 985.2	− 739.1	− 297.88; 100.08	− 305.79; 0.46	18,792	17,898	35.35	38.23	− 33,836.86	− 34,003.38	2228.72	2238.16
R357A	Inhibition	1927.404	1621.922	− 962	− 912.9	− 270.22; 69.95	− 307.03; 0.53	16,088	17,528	36.78	38.62	− 33,804.4	− 34,096.53	2238.17	2212.2
L359K	No inhibition	1932.877	1630.857	− 971.8	− 738.1	− 278.87; 0.47	− 272.44; 97.93	18,514	17,262	35.35	38.23	− 33,743.57	− 34,013.64	2223.76	2246.94
L359A	No inhibition	1926.131	1610.405	− 962.4	− 913.3	− 272.34; 0.50	− 276.91; 1.25	18,548	16,598	37.88	38.23	− 33,563.16	− 34,170.66	2234.3	2266.94
M383A	Inhibition	1914.113	1610.845	− 961.3	− 912.5	− 286.53; 0.58	− 299.44; 0.56	17,568	16,480	41.19	38.51	− 33,790.81	− 34,180.27	2234.91	2247.5
P389A	Inhibition	1916.738	1612.969	− 960.4	− 911.5	− 273.65; 0.70	− 286.32; 0.51	16,074	17,084	41.19	38.23	− 33,608.79	− 34,175.62	2226.31	2261.16
R393A	Inhibition	1916.493	1610.685	− 960.8	− 912.1	− 285.20; 81.44	− 317.40; 0.85	17,426	17,466	37.65	38.41	− 33,846.21	− 34,045.31	2237.16	2207.91
R559S	Inhibition	1913.935	1609.88	− 960.7	− 911.6	− 267.34; 0.96	− 305.16; 1.09	16,676	17,070	37.55	38.23	− 33,607.16	− 34,006.56	2177.5	2232.86
F603T	No inhibition	1914.033	1610.848	− 961.6	− 912.5	− 293.75; 89.30	− 275.07; 0.82	17,060	16,112	37.65	37.69	− 33,587.79	− 34,082.65	2228.48	2231.39
Q24K, A25A, K26E	Inhibition	1934.708	1768.052	− 972.2	− 836.3	− 253.81; 0.58	− 282.20; 78.93	17,048	17,718	38.69	38.23	− 33,594.24	− 34,085.15	2219.25	2242.31
M82N, Y83F, P84S	Inhibition	1917.424	1614.304	− 994.9	− 850.3	− 279.66; 0.56	− 275.54; 93.27	16,888	16,258	41.19	37.69	− 33,740.67	− 34,144.24	2229.51	2239.24
P135S, D136M	No inhibition	1926.164	1608.98	− 960.7	− 911.6	− 268.81; 1.07	− 265.21; 99.13	17,572	17,512	41.19	38.23	− 33,508.57	− 34,101.35	2230.71	2278.39
N338D, V339D, Q340R	No inhibition	1913.528	1618.9	− 960.7	− 912.5	− 268.07; 0.71	− 276.64; 0.58	18,142	16,304	38.39	35.9	− 33,591.98	− 34,121.34	2173.98	2251.51
S425P, P426S, D427N	Inhibition	1938.76	1763.944	− 991.6	− 881.8	− 290.44; 100.45	− 295.77; 80.78	18,552	17,512	38.39	38.23	− 33,581.53	− 34,180.03	2231.9	2264.97
K465Q, G466D, E467K	No inhibition	1952.777	1613.629	− 963.6	− 912.1	− 275.40; 38.72	− 275.26; 0.66	19,456	17,566	38.69	38.51	− 33,803.47	− 34,192.6	2228.99	2256.24

Structure-based docking by **ZDOCK** scores identified the ACE2 seven single mutants, K31D, Y41A, R219D, K309D, T324A, R559S, F603T, and a sequence with multiple mutants N338D/V339D/Q340R which have decreased binding affinity with SARS-CoV compared to wild-type (Table 7). Likewise, the ZDOCK score for SARS-CoV-2 identified eleven ACE2 single mutants, K31D, Y41A, R192D, H239Q, E312A, T324A, L359A, M383A, R393A, R559S, F603T, and a multiple mutant P135S/D136M, which have decreased binding affinity with SARS-CoV-2 compared to wild-type. However, the single mutant, R219D found to have the lowest binding affinity with SARS-CoV, while the multiple mutants, P135S/D136M, have the lowest binding affinity with SARS-CoV-2. Oppositely, increased binding affinity compared to wild-type was predicted for SARS-CoV with nineteen ACE2 single mutants, E37A, D38A, K68D, E110P, E160R, R192D, H239Q, E312A, D350A, K353H, K353A, K353D, D355A, R357A, L359K, L359A, M383A, P389A, and R393A. Similarly, the fifteen single mutants, E37A, D38A, K68D, E110P, E160R, R219D, K309D, D350A, K353H, K353A, K353D, D355A, R357A, L359K, and P389A, demonstrated the increased binding affinity compared to wild-type with SARS-CoV-2. Also, the increased binding affinity with SARS-CoV was identified for five multiple ACE2 mutants, Q24K/A25A/K26E, M82N/Y83F/P84S, P135S/D136M, S425P/P426S/D427N, and K465Q/G466D/E467K. Although, in the case of SARS-CoV-2, the increased binding affinity was identified for five multiple mutants, Q24K/A25A/K26E, M82N/Y83F/P84S, N338D/V339D/Q340R, S425P/P426S/D427N, and K465Q/G466D/E467K. Amidst all ACE2 mutants, the multiple mutant K465Q/G466D/E467K demonstrated the highest binding affinity with SARS-CoV, whereas the single mutant K309D demonstrated the highest binding affinity with SARS-CoV-2.

**ClusPro** docking score identified decreased binding affinity with SARS-CoV compared to wild-type complex for thirteen ACE2 single mutants (K31D, Y41A, K68D, R192D, H239Q, T324A, D350A, K353D, M383A, P389A, R393A, R559S, F603T), and two multiple mutants, P135S/D136M and N338D/V339D/Q340R. In contrast, ten single mutants (Y41A, K68D, R219D, D350A, K353H, K353A, K353D, D355A, L359K, P389A), and three multiple mutants, Q24K/A25A/K26E, M82N/Y83F/P84S, S425P/P426S/D427N recognized to have decreased binding affinity with SARS-CoV-2 compared to wild-type complex. The lowest binding affinity with SARS-CoV was identified for four ACE2 single mutants, Y41A, K68D, T324A, and P389A, while a single mutant L359K showed the lowest binding affinity with SARS-CoV-2. Contrariwise, the increased binding affinity with SARS-CoV was noted for thirteen ACE2 single mutants, E37A, D38A, E110P, E160R, R219D, K309D, E312A, K353H, K353A, D355A, R357A, L359K, L359A, and four multiple mutants Q24K/A25A/K26E, M82N/Y83F/P84S, S425P/P426S/D427N, and K465Q/G466D/E467K. Relatedly, the thirteen ACE2 single mutants, K31D, E37A, D38A, E110P, E160R, R192D, K309D, E312A, R357A, L359A M383A, R393A, and F603T, and two multiple mutants, N338D/V339D/Q340R, K465Q/G466D/E467K exhibited increased binding affinity with SARS-CoV-2. Although, a multiple mutant M82N/Y83F/P84S and a single mutant L359A have the highest binding affinity with SARS-CoV, and SARS-CoV-2, respectively. However, neutral (no change in binding affinity, their score is similar to wild type. Hence we are considering that they don’t change the binding affinity with SARS-CoV-2 and remains as No-Inhibition) was noted with wild-type SARS-CoV-2 for three ACE2 single mutants, H239Q, T324A, R559S and one multiple mutant, P135S/D136M.

The **HDOCK** docking score predicted decreased binding affinity with SARS-CoV for ACE2 five single mutants, K31D, E37A, Y41A, E160R, and E312A, and a multiple mutant Q24K/A25A/K26E. Likewise, ACE2 has six single mutants, K68D, E160R, D350A, L359K, L359A, F603T, and four multiples mutant M82N/Y83F/P84S, P135S/D136M, N338D/V339D/Q340R, K465Q/G466D/E467K showed decreased binding affinity with SARS-CoV-2. Nevertheless, ACE2 mutant, Y41A, has the lowest binding affinity with SARS-CoV compared to wild-type, whereas a multiple mutant P135S/D136M has the lowest binding affinity with SARS-CoV-2. Oppositely, SARS-CoV identified increased binding affinity for ACE2 twenty-one single mutants, D38A, K68D, E110P, R192D, R219D, H239Q, K309D, T324A, D350A, K353H, K353A, K353D, D355A, R357A, L359K, L359A, M383A, P389A, R393A, R559S, and F603T. Likewise, SARS-CoV-2 has increased binding affinity for twenty single mutants, K31D, E37A, D38A, Y41A, E110P, R192D, R219D, H239Q, K309D, E312A, T324A, K353H, K353A, K353D, D355A, R357A, M383A, P389A, R393A, and R559S. In addition, five multiple mutants, M82N/Y83F/P84S, P135S/D136M, N338D/V339D/Q340R, S425P/P426S/D427N, and K465Q/G466D/E467K, while two multiple mutants, Q24K/A25A/K26E and S425P/P426S/D427N showed increased binding affinity with SARS-CoV and SARS-CoV-2, respectively. Nonetheless, among all ACE2 mutants, a single mutant R219D, however, R393A displayed the highest binding affinity with SARS-CoV and SARS-CoV-2, respectively.

Furthermore, compared to ACE2 wild-type, **PatchDock** recognized increased binding affinity of SARS-CoV with all twenty-six ACE2 single mutants, K31D, E37A, D38A, Y41A, K68D, E110P, E160R, R192D, R219D, H239Q, K309D, E312A, T324A, D350A, K353H, K353A, K353D, D355A, R357A, L359K, L359A, M383A, P389A, R393A, R559S, and F603T. Similarly, the same pattern was also followed for six multiple mutants, Q24K/A25A/K26E, M82N/Y83F/P84S, P135S/D136M, N338D/V339D/Q340R, S425P/P426S/D427N, and K465Q/G466D/E467K, while mutant K465Q/G466D/E467K have a highest binding affinity with SARS-CoV. Inversely, decreased binding affinity of SARS-CoV-2 was identified for twenty-four single mutants, K31D, E37A, D38A, K68D, E110P, E160R, R192D, R219D, H239Q, K309D, E312A, T324A, D350A, K353H, K353A, K353D, R357A, L359K, L359A, M383A, P389A, R393A, R559S, and F603T. Also, decreased binding affinity of SARS-CoV-2 has been identified with six multiple mutants, Q24K/A25A/K26E, M82N/Y83F/P84S, P135S/D136M, N338D/V339D/Q340R, S425P/P426S/D427N, and K465Q/G466D/E467K. Although ACE2 single mutant, F603T, attained the lowest binding affinity with SARS-CoV-2. In contrast, only two single mutants, Y41A, and D355A have increased binding affinity with SARS-CoV-2, while Y41A was identified as the highest binding affinity mutant with SARS-CoV-2.

**InterEvDock2** docking score displayed decreased binding affinity with SARS-CoV for fourteen single ACE2 mutants, E37A, Y41A, E160R, R219D, E312A, T324A, K353A, D355A, R357A, L359K, L359A, R393A, R559S, and F603T. Likewise, decreased binding affinity of SARS-CoV was also obtained for four multiple mutants, Q24K/A25A/K26E, N338D/V339D/Q340R, S425P/P426S/D427N, and K465Q/G466D/E467K. However, the lowest binding affinity was noted for the L359K mutant. Relatively, three single ACE2 mutants, R192D, R219D, and F603T, illustrated decreased binding affinity with SARS-CoV-2. Also, two multiple mutants, M82N/Y83F/P84S and N338D/V339D/Q340R exhibited decreased binding affinity with SARS-CoV-2 wherein mutant N338D/V339D/Q340R attained the lowest binding affinity. Conversely, the highest binding affinity with a similar docking score of SARS-CoV was perceived for twelve ACE2 single mutants, K31D, D38A, K68D, E110P, R192D, H239Q, K309D, D350A, K353H, K353D, M383A, and P389A, and two multiple mutants, M82N/Y83F/P84S and P135S/D136M. Also, nine single mutants, E37A, D38A, Y41A, K309D, E312A, K353A, R357A, M383A, and R393A, and a multiple mutant, K465Q/G466D/E467K, perceived the increased binding affinity with SARS-CoV-2, while four single mutants, E37A, Y41A, K353A, R357A attained highest binding affinity. However, neutral was noted with wild-type SARS-CoV-2 for fourteen ACE2 single mutants, K31D, K68D, E110P, E160R, H239Q, T324A, D350A, K353H, K353D, D355A, L359K, L359A, P389A, and R559S, and three multiple mutants, Q24K/A25A/K26E, P135S/D136M, and S425P/P426S/D427N.

**SOAP-PP** classified the decreased binding affinity with SARS-CoV compared to wild-type complex for twenty-one single mutants, K31D, E37A, D38A, K68D, E110P, E160R, R192D, H239Q, K309D, T324A, D350A, K353H, K353A, K353D, R357A, L359K, L359A, M383A, P389A, R559S, and F603T. Correspondingly, decreased binding affinity was also noted for all six multiple mutants, Q24K/A25A/K26E, M82N/Y83F/P84S, P135S/D136M, N338D/V339D/Q340R, S425P/P426S/D427N, and K465Q/G466D/E467K wherein, P135S/D136M mutant having lowest binding affinity. In the same way, twenty-two single mutants, K31D, E37A, D38A, Y41A, K68D, E160R, R219D, H239Q, K309D, E312A, T324A, D350A, K353H, K353A, K353D, R357A, L359K, L359A, P389A, R393A, R559S, and F603T achieved the decreased binding affinity with SARS-CoV-2. Although, all four multiple mutants, Q24K/A25A/K26E, M82N/Y83F/P84S, P135S/D136M, and N338D/V339D/Q340R, acquired decreased binding affinity wherein, a single mutant D355A mutant had the lowest binding affinity. Oppositely, five single mutants, Y41A, R219D, E312A, D355A, and R393A, have increased binding affinity with SARS-CoV, whereas mutant R219D demonstrated the highest binding affinity. Congruently, increased binding affinity was identified for four single mutants, E110P, R192D, K353D, and M383A, and two multiple mutants, S425P/P426S/D427N, K465Q/G466D/E467K have wherein, R192D demonstrated the highest binding affinity with SARS-CoV-2.

**FRODOCK2** identified the decreased binding affinity with SARS-CoV compared to wild-type complex for sixteen single mutants, K68D, E110P, E160R, R192D, R219D, H239Q, K309D, T324A, D350A, K353A, K353D, D355A, L359K, P389A, R559S, and F603T. Alike, lower binding affinity was envisaged for three multiple mutants, Q24K/A25A/K26E, M82N/Y83F/P84S, N338D/V339D/Q340R, and K465Q/G466D/E467K, though N338D/V339D/Q340R obtained to have the lowest binding affinity. Nevertheless, eighteen single mutants, K31D, E37A, D38A, Y41A, E110P, E160R, R219D, E312A, T324A, K353A, K353D, D355A, R357A, L359K, M383A, R393A, R559S, and F603T was identified with decreased binding affinity for SARS-CoV-2. Correspondingly, four multiple mutants, Q24K/A25A/K26E, M82N/Y83F/P84S, N338D/V339D/Q340R, and K465Q/G466D/E467K, envisaged decreased binding affinity, though a single mutant R219D attained the lowest binding affinity. Oppositely, the increased binding affinity of SARS-CoV was also identified for ten single ACE2 mutants, K31D, E37A, D38A, Y41A, E312A, K353H, R357A, L359A, M383A, and R393A, wherein Y41A attained the highest binding affinity. Similarly, eight single mutants, K68D, R192D, H239Q, K309D, D350A, K353H, L359A, and P389A, have increased binding affinity, in which, highest binding affinity has been identified for mutant, K353H with SARS-CoV-2. Notably, two multiple mutants, P135S/D136M and S425P/P426S/D427N, have enhanced binding affinity with both SARS-CoV and SARS-CoV-2.

#### Patient samples ACE2 mutations affect the binding affinity with SARS-CoV and SARS-CoV-2

The docking of 155 ACE2 mutants of patient samples was performed with SARS-CoV and SARS-CoV-2 (Table 8). A comparative analysis of docked complexes revealed primarily the hotspot mutants having inhibition and no-inhibition interacting effect with SARS-CoV and SARS-CoV-2 predicted using several tools. Secondly, the binding effect of common mutants with SARS-CoV and SARS-CoV-2 have been identified using docking tools (ZDOCK, ClusPro, HDOCK, PatchDock, InterEvDock2, SOAP-PP, FRODOCK2). In addition, the comparative analysis was also performed for predicted docking results between all the tools used with ClusPro since it was identified as the reliable tool in terms of specificity, sensitivity, and accuracy as observed in our Experimental findings. Finally, a comparative analysis was performed between mutant complexes of patients and experimental samples to identify common mutants.

**Table 8:** Docking between patients’ ACE2 mutants with SARS-CoV, and SARS-CoV-2 respectively.

Mutants	ZDOCK (score)		ClusPro (score)		HDOCK (score; ligand rmsd (Å))		PatchDock (score)		InterEvDock2 (score)		SOAP-PP (score)		FRODOCK2 (score)	
	SARS-CoV	SARS-CoV-2	SARS-CoV	SARS-CoV-2	SARS-CoV	SARS-CoV-2	SARS-CoV	SARS-CoV-2	SARS-CoV	SARS-CoV-2	SARS-CoV	SARS-CoV-2	SARS-CoV	SARS-CoV-2
WILD TYPE	1914.104	1610.913	− 961.7	− 911.6	− 263.89; 1.21	− 279.45; 0.53	15,788	17,820	39.33	38.23	− 33,829.06	− 34,179.68	2230.49	2256.97
L8F	1914.1	1610.914	− 961.7	− 911.6	− 263.89; 1.21	− 279.45; 0.53	15,788	17,820	39.33	38.23	− 33,829.06	− 34,179.68	2230.49	2256.97
T20I	1914.102	1610.912	− 961.7	− 961.7	− 263.89; 1.21	− 279.45; 0.54	15,788	17,820	39.33	38.23	− 33,829.06	− 34,179.68	2230.49	2256.97
E22D	1927.216	1609.48	− 962.4	− 962.4	− 250.89; 42.07	− 275.90; 0.53	18,278	17,028	38.39	38.23	− 33,619.84	− 34,180.5	2226.89	2243.8
F28L	1916.274	1621.616	− 964.3	− 908.9	− 297.86; 77.72	− 269.54; 83.32	19,028	17,158	41.19	38.62	− 33,752.33	− 34,060.3	2204.94	2248.9
H34N	1926.15	1608.957	− 960.7	− 911.6	− 254.90; 102.42	− 281.19; 0.47	16,488	17,528	37.65	38.51	− 33,808.69	− 34,195.51	2230.77	2245.42
E35K	1915.408	1610.893	− 961.6	− 912.4	− 277.68; 90.85	− 285.83; 0.58	16,884	17,038	37.65	38.23	− 33,855.92	− 34,144.7	2229.98	2275.92
E37K	1926.458	1608.745	− 961.6	− 911.6	− 288.75; 61.13	− 288.00; 0.40	19,408	17,346	38.39	38.51	− 33,582.18	− 34,184.56	2230.34	2272.75
L39M	1916.564	1612.019	− 960.4	− 911.5	− 253.56; 91.26	− 276.43; 0.34	17,660	17,052	41.19	38.51	− 33,612.42	− 34,165.92	2228.97	2263.36
S44L	1927.701	1626.409	− 966.4	− 909.1	− 293.99; 80.69	− 271.25; 0.82	17,074	17,082	41.19	38.23	− 33,661.03	− 34,128.87	2218.53	2214.64
V59D	1914.087	1618.8	− 962.4	− 911.5	− 260.17; 0.77	− 269.10; 1.09	17,710	17,028	37.65	38.23	− 33,599.99	− 34,130.02	2226.74	2246.47
F72C	1914.045	1610.891	− 960.5	− 911.5	− 263.30; 93.73	− 289.28; 0.47	19,058	18,524	41.19	38.62	− 33,810.35	− 34,032.71	2204.62	2192.08
L73S	1914.114	1610.837	− 961.6	− 912.4	− 261.65; 96.71	− 293.29; 0.49	16,042	16,480	41.19	38.23	− 33,781.04	− 34,170.08	2229.82	2235.07
A99S	1915.113	1613.969	− 961.4	− 911.5	− 260.76; 0.70	− 279.72; 0.34	15,826	17,678	41.19	38.23	− 33,594.35	− 34,107.66	2229.47	2277.67
S109L	1925.698	1622.094	− 962	− 913	− 281.02; 78.66	− 264.79; 1.87	15,968	16,660	38.39	35.9	− 33,628.66	− 34,173.11	2233.57	2272.29
R115Q	1950.445	1614.027	− 962.3	− 912.5	− 285.35; 90.63	− 266.00; 94.20	16,688	16,788	41.19	38.23	− 33,585.42	− 34,153.91	2229.73	2215.04
R115W	1917.204	1608.963	− 961.6	− 911.6	− 273.64; 80.93	− 269.43; 0.54	16,800	16,836	40.1	37.69	− 33,603.85	− 34,126.31	2246.57	2208.74
L116F	1939.675	1627.228	− 958.8	− 909.5	− 278.27; 0.33	− 284.60; 83.53	16,598	16,532	40.1	37.6	− 33,574.18	− 34,127.27	2225.15	2225.72
P138S	1914.01	1610.802	− 960.5	− 911.5	− 267.69; 0.48	− 279.01; 97.58	15,934	17,820	41.19	38.23	− 33,809.07	− 34,151.2	2229.86	2265.56
G147V	1941.14	1618.33	− 962.1	− 810.7	− 279.12; 81.63	− 291.88; 0.79	16,306	17,664	37.85	38.23	− 33,637.46	− 34,130.84	2224.4	2215.66
L162F	1942.819	1624.987	− 963.1	− 914.5	− 267.26; 1.15	− 287.10; 0.42	17,266	17,420	40.1	38.23	− 33,580.11	− 34,142.78	2239.08	2274.55
E182D	1915.38	1610.401	− 962.4	− 911.5	− 275.30; 90.09	− 292.56; 0.50	16,256	17,452	41.19	38.23	− 33,536.79	− 34,159.85	2229.22	2248.59
E189K	1927.026	1622.087	− 961.3	− 912.5	− 275.56; 95.75	− 288.87; 0.56	19,412	16,084	38.39	38.23	− 33,639.07	− 34,208.64	2229.55	2272.16
H195Y	1915.985	1611.373	− 959.4	− 912	− 288.43; 80.40	− 287.87; 1.02	16,346	17,496	35.61	38.23	− 33,689.66	− 34,089.83	2233.06	2282.74
Y202H	1946.586	1621.069	− 962.9	− 913.8	− 256.95; 0.68	− 301.04; 78.68	16,440	16,234	38.69	38.23	− 33,612.02	− 34,018.69	2222.92	2265.6
R204I	1773.942	1647.72	− 961.6	− 904.4	− 273.61; 96.87	− 289.16; 0.49	17,420	16,398	36.78	37.89	− 33,909.46	− 34,121.67	2216.08	2167.37
G205V	1773.803	1634.116	− 961.6	− 904.4	− 256.45; 89.25	− 276.24; 0.54	16,566	16,578	36.78	36.44	− 33,749.92	− 34,157.62	2233.73	2172.21
D206Y	1925.751	1621.942	− 962	− 913	− 271.31; 96.57	− 274.89; 0.85	18,580	17,012	41.19	37.69	− 33,574.86	− 34,076.37	2233.9	2274.56
G211W	1926.261	1608.225	− 963.5	− 913	− 277.00; 0.50	− 279.19; 0.53	15,860	16,654	42.6	37.69	− 33,518.59	− 34,118.58	2261.19	2235.95
V212I	1924.635	1614.811	− 961.7	− 917.7	− 257.84; 91.79	− 278.56; 0.61	19,240	17,064	41.19	38.23	− 33,553.2	− 34,184.51	2232.54	2270.47
D213G	1915.108	1740.458	− 972.8	− 896.5	− 275.49; 0.33	− 287.31; 0.45	18,110	17,374	37.65	38.51	− 33,770.38	− 34,013.84	2192.09	2241.16
R219P	1773.905	1649.284	− 963.3	− 905.3	− 278.99; 77.39	− 283.31; 104.32	16,922	16,626	36.78	37.89	− 33,898.12	− 34,102.17	2206.08	2182.32
R219H	1773.917	1649.255	− 963.7	− 904.9	− 262.38; 96.56	− 291.80; 83.42	16,762	16,672	36.78	37.89	− 33,921.48	− 34,143.39	2234.72	2163.69
G220C	1933.236	1629.269	− 983.8	− 738.9	− 298.26; 89.53	− 293.87; 0.53	18,802	17,350	35.28	38.23	− 33,459.72	− 34,190.43	2221.01	2241.49
E232K	1950.664	1611.83	− 963.4	− 912.2	− 255.82; 0.58	− 288.15; 0.49	19,366	17,044	37.65	38.23	− 33,549.81	− 34,166.91	2229.1	2265.19
A242T	1927.239	1619.071	− 961.9	− 915.2	− 255.97; 28.89	− 280.40; 0.92	16,890	17,554	41.19	38.23	− 33,599.69	− 34,166.81	2222.04	2235.67
I256M	1914.335	1609.782	− 960.7	− 911.6	− 277.51; 99.70	− 289.31; 0.59	18,036	16,634	39.33	38.23	− 33,554.65	− 34,185.66	2230.14	2266.75
A264S	1914.06	1610.433	− 962.4	− 912.1	− 271.29; 0.32	− 295.92; 0.51	17,030	16,190	38.69	38.23	− 33,608.11	− 34,114.1	2230.49	2244.66
D269N	1914.086	1610.88	− 961.6	− 781.4	− 289.13; 1.21	− 277.51; 42.56	18,662	16,702	41.19	38.51	− 33,638.56	− 34,125.3	2230.16	2266.61
D269Y	1927.973	1609.266	− 960.2	− 810.6	− 256.44; 69.09	− 279.97; 2.25	19,344	16,294	38.39	38.23	− 33,601.76	− 34,150.77	2223.02	2263.44
G272C	1922.116	1607.33	− 962.8	− 908.6	− 266.69; 76.13	− 298.34; 0.79	16,242	17,444	41.19	38.51	− 33,575.83	− 34,192.65	2230.57	2240.2
R273K	1914.375	1609.731	− 960.7	− 911.6	− 264.18; 80.36	− 295.52; 0.39	16,384	17,454	38.39	38.23	− 33,597.66	− 34,092.93	2229.93	2269.3
S280Y	1915.339	1829.164	− 961.6	− 912.4	− 281.25; 81.24	− 286.14; 0.80	16,434	16,262	41.19	38.23	− 33,816.57	− 34,014.4	2202.54	2250.29
V293I	1926.933	1610.808	− 960.5	− 911.5	− 274.91; 0.54	− 275.50; 0.44	17,644	16,108	41.19	38.23	− 33,581.89	− 34,227.48	2228.7	2256.92
A296T	1954.014	1612.893	− 960.4	− 911.5	− 269.23; 0.44	− 289.53; 0.69	18,022	17,452	41.19	38.23	− 33,598.63	− 34,108.63	2225.77	2250.91
Q305L	1917.253	1608.41	− 959.4	− 912	− 272.37; 0.52	− 295.29; 0.86	18,396	16,936	38.69	38.51	− 33,547.78	− 34,100.92	2230	2262.45
A311V	1926.076	1622.623	− 961.4	− 913.3	− 285.05; 1.00	− 282.64; 1.14	16,664	16,356	41.19	38.23	− 33,538.68	− 34,124.56	2223.15	2258.09
S317F	1914.035	1610.447	− 962.4	− 911.5	− 278.61; 80.89	− 297.74; 0.56	18,278	17,044	41.19	37.69	− 33,600.05	− 34,073.91	2197.77	2270.87
L320F	1914.622	1623.661	− 959	− 911.7	− 281.26; 0.54	− 283.33; 55.85	17,364	17,184	41.19	38.23	− 33,619.71	− 34,114.07	2231.61	2283.12
T324S	1916.702	1612.086	− 961.5	− 911.6	− 273.67; 0.96	− 282.86; 0.98	18,014	17,474	41.19	38.23	− 33,633.93	− 34,175.56	2230.26	2262.84
Q325P	1770.274	1812.557	− 977	− 858.9	− 260.59; 94.56	− 281.23; 1.59	17,806	16,754	38.69	38.51	− 33,637.5	− 34,239.34	2216.71	2249.25
N330H	1927.949	1609.007	− 961.7	− 911.6	− 279.75; 76.72	− 276.87; 77.77	16,212	17,452	38.39	38.23	− 33,551.93	− 34,100.94	2228.05	2242.44
T334R	1951.492	1612.112	− 962.2	− 912.1	− 271.57; 0.71	− 295.83; 0.45	17,572	16,620	39.33	37.69	− 33,608.55	− 34,040.4	2188.19	2296.99
G337E	1915.166	1610.85	− 960.4	− 805.3	− 272.63; 60.73	− 299.24; 0.43	18,398	16,582	40.1	37.69	− 33,598.46	− 34,116.96	2194.71	2269.73
N338D	1950.328	1610.33	− 960.7	− 782.2	− 255.18; 79.31	− 290.18; 0.45	18,056	16,692	41.19	38.23	− 33,551.92	− 34,174.68	2229.35	2264.15
G352W	1939.127	1625.598	− 962.5	− 906.6	− 287.72; 0.49	− 268.01; 0.34	16,814	17,758	40.1	37.69	− 33,633.78	− 34,109.08	2209.14	2201.38
D355N	1913.994	1610.459	− 961.2	− 911.6	− 274.54; 111.32	− 292.03; 0.60	17,292	16,204	39.33	38.23	− 33,605.25	− 34,117.41	2230.06	2256.12
R357S	1933.044	1631.369	− 966.3	− 738.9	− 262.99; 65.99	− 276.09; 92.04	18,690	16,316	35.35	38.23	− 33,615.45	− 34,055.94	2225	2203.31
I358F	1941.468	1623.286	− 961.3	− 913.7	− 257.21; 60.47	− 304.11; 0.55	16,422	17,500	41.19	38.23	− 33,627.15	− 34,123.08	2222.08	2241.92
V364A	1927.001	1610.927	− 960.4	− 911.6	− 270.35; 104.05	− 282.68; 0.48	19,102	17,466	37.65	38.23	− 33,627.74	− 34,144.54	2234.04	2261.79
D367V	1949.248	1613.781	− 963	− 912.1	− 282.04; 0.75	− 293.93; 106.68	18,702	17,038	41.19	38.23	− 33,619.31	− 34,105.59	2228.91	2475.44
D368N	1926.263	1607.891	− 960.7	− 911.6	− 259.65; 91.05	− 292.50; 0.85	18,004	17,116	41.19	38.23	− 33,590.91	− 34,043.62	2231.11	2268.04
M383I	1912.584	1610.852	− 960.5	− 911.5	− 268.22; 69.56	− 283.82; 0.92	16,802	17,056	39.33	38.51	− 33,621.05	− 34,168.91	2229.57	2255.53
R393G	1925.044	1608.024	− 960.7	− 912	− 271.51; 69.93	− 278.65; 0.49	17,794	17,508	36.78	38.41	− 33,817.08	− 34,000.56	2175.26	2194.2
G395V	1930.383	1621.012	− 961.3	− 914	− 264.40; 80.63	− 278.92; 0.58	16,942	16,200	41.19	38.23	− 33,623.08	− 34,084.88	2223.65	2232.13
E398K	1925.828	1622.158	− 961.3	− 912.9	− 274.10; 1.14	− 297.21; 0.70	18,200	17,314	38.39	37.69	− 33,599.43	− 34,195.94	2230.96	2275.01
G399R	1951.405	1615.018	− 963.4	− 906.9	− 295.37; 33.28	− 300.66; 0.37	16,800	16,484	41.19	38.23	− 33,601.94	− 34,095.46	2235.74	2249.36
A403V	1926.454	1609.908	− 960.7	− 911.6	− 277.78; 78.96	− 284.83; 0.84	16,490	17,512	41.19	38.23	− 33,545.67	− 34,100.98	2231.41	2270.64
G405W	1942.088	1623.878	− 962.5	− 784.4	− 278.30; 91.20	− 282.88; 0.64	17,674	17,470	40.1	38.23	− 33,691.82	− 34,103.79	2199.59	2272.87
E406K	1914.326	1610.798	− 960.7	− 912	− 260.86; 69.11	− 279.75; 0.70	17,288	16,360	37.65	38.23	− 33,608.59	− 34,097.59	2229.9	2254.54
S409L	1940.494	1614.489	− 961.4	− 913.5	− 262.32; 96.74	− 284.79; 0.35	17,344	18,422	41.19	38.23	− 33,586.02	− 34,071.14	2221.89	2233.53
A412T	1941.115	1619.277	− 962.1	− 912.5	− 284.56; 0.91	− 301.72; 0.49	17,158	16,686	40.1	38.23	− 33,560.88	− 34,113.47	2222.29	2251.82
K419T	1927.656	1620.214	− 962.7	− 914.5	− 255.78; 90.98	− 299.91; 0.42	16,834	17,506	37.65	37.69	− 33,627.28	− 34,181.03	2233.04	2252.88
P426L	1916.61	1617.805	− 960.4	− 911.5	− 268.99; 1.03	− 260.65; 0.48	16,662	17,060	41.19	38.23	− 33,569.35	− 34,150.06	2228.79	2256.23
P426S	1914.352	1615.614	− 960.7	− 911.6	− 271.83; 0.37	− 285.03; 0.42	16,214	17,452	38.39	38.23	− 33,622.99	− 34,164.78	2228.72	2265.92
D431G	1915.092	1613.899	− 960.8	− 912.1	− 274.52; 1.44	− 279.72; 82.96	19,184	17,996	37.65	37.69	− 33,590.25	− 34,015.4	2227.64	2228.01
L450P	1938.531	1624.02	− 962.5	− 913.8	− 270.54; 0.37	− 308.14; 0.53	17,380	17,088	37.65	38.23	− 33,595.54	− 34,158.63	2229.11	2235.62
K458T	1928.983	1621.88	− 962	− 913	− 291.71; 0.84	− 284.41; 99.58	17,792	17,276	38.39	38.23	− 33,635.41	− 34,138.54	2230.06	2272.59
M462I	1939.271	1611.647	− 962.3	− 913.1	− 261.06; 98.30	− 307.57; 0.42	17,604	17,484	41.19	38.23	− 33,533.03	− 34,089.71	2229.07	2237.6
I468T	1926.607	1610.972	− 960.7	− 911.6	− 280.66; 99.07	− 264.28; 108.16	18,312	17,090	37.65	38.23	− 33,589.76	− 34,163.8	2228.99	2257.37
W473L	1915.544	1611.055	− 960.2	− 912.4	− 272.80; 76.48	− 291.99; 0.49	17,436	17,196	41.19	38.23	− 33,601.27	− 34,086.87	2230.22	2252.19
W477R	1924.275	1610.976	− 961.5	− 912.8	− 293.77; 77.55	− 291.17; 0.50	17,102	16,426	38.39	38.23	− 33,506.45	− 34,093.36	2230.39	2280.89
V488M	1942.587	1627.169	− 962.6	− 901.9	− 260.50; 102.87	− 280.83; 0.66	16,414	16,868	41.9	37.69	− 33,870.11	− 33,999.78	2278.04	2328.32
E489K	1926.202	1609.061	− 961.3	− 911.6	− 254.89; 1.11	− 287.15; 0.53	16,778	17,526	37.65	38.23	− 33,754.47	− 34,183.51	2230.39	2259.15
V491L	1944.951	1618.924	− 963.1	− 914.5	− 260.94; 91.68	− 294.36; 0.78	16,784	17,602	41.19	38.51	− 33,616.68	− 34,196.41	2225.24	2262.08
D494G	1913.555	1610.56	− 960.5	− 911.6	− 269.77; 96.80	− 278.71; 0.59	16,666	16,212	41.19	38.23	− 33,604.28	− 34,090.45	2231.4	2220.61
T496A	1916.127	1609.038	− 959.9	− 912.2	− 258.41; 23.42	− 280.25; 1.69	16,906	17,012	41.19	38.51	− 33,619.27	− 34,205.13	2230.82	2227.97
R518M	1927.229	1622.119	− 962	− 913	− 270.00; 99.30	− 282.94; 1.45	16,642	17,068	38.39	38.23	− 33,573.65	− 34,178.56	2231.69	2270.01
G561R	1947.256	1633.095	− 966.3	− 764.3	− 280.73; 34.10	− 285.48; 0.99	18,196	17,276	43.31	38.23	− 33,616.12	− 34,017.44	2173.42	2334.38
P565L	1929.859	1628.318	− 956.5	− 907.4	− 262.35; 111.38	− 279.17; 1.28	17,774	17,048	41.19	38.23	− 33,612.79	− 34,135.45	2225.74	2248.07
K577N	1915.27	1610.472	− 960.9	− 912.6	− 268.50; 33.08	− 293.97; 86.32	18,610	17,558	38.39	38.23	− 33,618.58	− 34,195.34	2226.07	2251.18
M579T	1914.104	1610.938	− 961.6	− 911.6	− 250.88; 109.07	− 286.48; 0.89	17,012	17,036	38.39	38.23	− 33,814.89	− 34,176.86	2229.68	2253.6
V581I	1915.08	1612.485	− 960.8	− 911.5	− 262.13; 0.40	− 287.76; 0.37	17,980	17,604	41.19	38.23	− 33,554.1	− 34,170.12	2228.07	2263.57
P590L	1926.151	1608.986	− 961.3	− 911.6	− 281.51; 61.79	− 282.22; 118.83	18,024	17,272	36.78	38.23	− 33,448.05	− 34,146.9	2231.8	2249.4
D597E	1937.826	1625.98	− 962.9	− 912.1	− 262.89; 0.31	− 291.30; 0.51	17,664	19,276	35.35	38.23	− 33,491.98	− 34,197.65	2228.79	2252.88
N599K	1945.128	1606.033	− 963.3	− 768	− 277.96; 107.66	− 294.67; 0.47	16,984	16,944	41.19	38.23	− 33,564.83	− 33,999.92	2244.39	2279.22
K600N	1916.027	1622.078	− 962	− 913	− 266.42; 99.90	− 283.10; 0.56	17,788	17,682	38.39	38.23	− 33,632.9	− 34,091.69	2233.8	2272.42
N601I	1917.861	1651.027	− 961.4	− 911.6	− 287.80; 0.50	− 285.52; 92.22	15,950	17,094	39.33	38.23	− 33,655.88	− 34,147.71	2229.76	2265.7
D609N	1946.95	1625.81	− 961.7	− 776.8	− 263.31; 33.43	− 283.87; 0.59	16,822	18,902	38.69	38.23	− 33,802.22	− 33,994.14	2226.29	2243.61
Y613H	1926.26	1607.954	− 960.6	− 911.6	− 273.86; 80.08	− 275.91; 0.48	18,004	16,640	41.19	38.23	− 33,521.97	− 34,089.63	2228.99	2277.66
D615Y	1952.679	1680.146	− 963.3	− 912	− 261.00; 108.65	− 288.67; 0.47	17,052	17,316	41.19	38.51	− 33,671.64	− 34,073.22	2227.27	2241.96
I618M	1951.03	1615.602	− 963.5	− 911.5	− 266.24; 77.69	− 294.23; 0.52	18,292	18,336	38.69	38.23	− 33,557.27	− 34,008.38	2227.31	2249.2
K625T	1915.32	1608.565	− 959.9	− 911.5	− 256.01; 0.63	− 272.01; 0.46	16,934	17,092	41.19	38.23	− 33,566.58	− 34,065.77	2220.71	2256.83
L628F	1946.306	1635.851	− 962.7	− 902.6	− 293.94; 96.78	− 310.31; 0.52	17,066	16,660	39.4	37.69	− 33,767.44	− 33,966.23	2266.73	2367.03
R644Q	1913.974	1610.45	− 962.4	− 911.6	− 280.25; 39.00	− 295.98; 1.13	16,226	16,206	41.19	38.23	− 33,602.98	− 34,083.1	2229.47	2219.9
E667K	1926.045	1609.707	− 961.1	− 912	− 280.78; 77.89	− 287.42; 0.57	16,796	16,000	41.19	38.23	− 33,587.26	− 34,121.98	2229.68	2215.08
V670L	1927.53	1623.765	− 962.7	− 913.9	− 275.30; 32.84	− 268.31; 0.58	17,610	17,628	40.1	38.23	− 33,612.11	− 34,117.06	2228.48	2222.43
V672A	1916.601	1612.967	− 960.4	− 911.5	− 267.01; 93.21	− 294.38; 77.30	18,014	17,456	41.19	38.23	− 33,591.05	− 34,107.03	2229.2	2266.85
K676E	1926.567	1609.629	− 958.9	− 912.1	− 285.04; 33.43	− 279.49; 99.16	17,598	16,846	38.69	38.23	− 33,540.36	− 34,120.11	2230.67	2267.43
F683L	1951.23	1610.805	− 963.4	− 912.2	− 290.27; 97.28	− 291.78; 0.37	16,764	17,026	37.65	38.23	− 33,584.63	− 34,082.05	2231.26	2265.13
S692F	1927.377	1613.51	− 961.1	− 912.6	− 281.71; 99.02	− 286.30; 1.01	16,140	17,360	37.65	38.51	− 33,769.28	− 34,017.29	2190.9	2254.06
D693N	1924.582	1622.066	− 962	− 913	− 281.33; 0.93	− 288.33; 0.65	19,142	16,548	37.65	38.23	− 33,598.45	− 34,191.06	2231.7	2260.95
I694M	1913.998	1610.799	− 960.5	− 911.5	− 271.61; 62.91	− 285.05; 0.58	19,374	17,820	37.65	38.23	− 33,848.6	− 34,174.33	2229.84	2252.36
E701K	1915.085	1610.094	− 958	− 911.7	− 266.04; 95.82	− 292.65; 0.53	16,026	17,042	37.65	38.23	-33,526.21	− 34,148.52	2230.54	2269.55
R708Q	1927.112	1607.689	− 961.5	− 911.6	− 262.70; 96.95	− 292.17; 1.03	18,004	16,610	37.65	38.23	− 33,556.9	− 34,111.4	2228.83	2261.58
D713N	1948.261	1612.959	− 962.9	− 912.2	− 303.56; 0.63	− 315.48; 0.62	18,448	17,566	41.19	38.23	− 33,589.62	− 34,168.71	2233.76	2264.96
R716H	1926.877	1607.87	− 960.7	− 911.6	− 278.70; 96.31	− 287.72; 0.54	17,994	17,328	41.19	38.23	− 33,563.38	− 34,179.14	2223.67	2262.36
R716C	1916.087	1610.878	− 960.4	− 911.5	− 270.94; 0.49	− 297.73; 77.63	16,486	17,048	42.6	37.69	− 33,582.6	− 34,020	2196.46	2281.35
N720S	1926.549	1609.136	− 961.1	− 911.5	− 261.67; 80.16	− 276.59; 0.29	16,490	17,526	37.65	38.23	− 33,528.28	− 34,054.43	2228.04	2258.44
P737H	1921.213	1610.478	− 1039.1	− 778.4	− 277.53; 1.01	− 283.88; 0.43	19,426	17,052	37.65	37.69	− 33,879.03	− 34,202.22	2322.36	2198.45
P737L	1833.521	1650.426	− 975.7	− 846.9	− 265.59; 94.88	− 289.02; 0.38	19,860	16,856	37.65	38.23	− 33,783.23	− 34,166.94	2276.48	2307.35
V748F	2136.521	1816.646	− 948.9	− 759.7	− 258.99; 60.93	− 270.87; 0.73	16,408	17,694	35.44	38.28	− 33,848.36	− 34,125.47	2302.32	2210.1
L760M	1912.739	1614.121	− 961	− 956.9	− 287.91; 33.04	− 293.28; 0.70	16,726	17,098	38.85	38.23	− 33,589.94	− 34,213.22	2231.15	2270.84
I761T	1937.419	1607.53	− 960.1	− 909.2	− 263.10; 0.47	− 299.72; 0.37	19,430	17,506	41.19	35.62	− 33,649.98	− 34,157.4	2200.81	2244.86
F762L	1939.425	1605.604	− 961.5	− 900.6	− 272.75; 77.89	− 293.88; 0.39	16,044	17,478	40.1	38.4	− 33,539.7	− 34,161.24	2206.24	2287.54
G764R	1938.713	1625.793	− 962.9	− 778.4	− 273.22; 102.61	− 296.70; 0.65	18,560	17,004	37.55	38.63	− 33,431.36	− 34,204.01	2257.19	2194.8
R766K	1926.261	1605.34	− 983.2	− 743.6	− 273.13; 39.15	− 299.04; 0.54	16,040	16,350	40.1	37.69	− 33,586.07	− 34,096.34	2218.43	2290.91
R768L	1966.992	1615.061	− 961	− 932.1	− 260.52; 32.09	− 286.60; 0.56	18,996	17,198	40.1	38.34	− 33,564.45	− 34,014.5	2174.58	2261.15
R768W	1962.501	1604.801	− 963	− 952	− 266.59; 97.60	− 289.36; 0..52	19,094	17,620	38.44	38.65	− 33,871.42	− 34,073.86	2238.65	2231.14
R775I	1914.101	1610.913	− 961.7	− 911.6	− 263.89; 1.21	− 279.45; 0.53	15,788	17,820	39.33	38.23	− 33,829.06	− 34,179.68	2230.49	2256.97
P780S	1914.101	1610.911	− 961.7	− 911.6	− 263.89; 1.21	− 279.45; 0.53	15,788	17,820	39.33	38.23	− 33,839.06	− 34,179.68	2230.49	2256.97
D785N	1914.099	1610.911	− 961.7	− 911.6	− 263.89; 1.21	− 279.45; 0.53	15,788	17,820	39.33	38.23	− 33,829.06	− 34,179.68	2230.49	2256.97
G789R	1914.103	1610.912	− 961.7	− 911.6	− 263.89; 1.21	− 279.45; 0.53	15,788	17,820	39.33	38.23	− 33,829.06	− 34,179.68	2230.49	2256.97
T798P	1914.104	1610.913	− 961.7	− 911.6	− 263.89; 1.21	− 279.45; 0.53	15,788	17,820	39.33	38.23	− 33,829.06	− 34,179.68	2230.49	2256.97
T803I	1914.103	1610.911	− 961.7	− 911.6	− 263.89; 1.21	− 279.45; 0.54	15,788	17,820	39.33	38.23	− 33,829.06	− 34,179.68	2230.49	2256.97
S47C, P284S	1915.08	1612.525	− 960.8	− 911.5	− 271.12; 0.48	− 299.26; 0.52	18,026	15,932	37.65	38.23	− 33,572.27	− 34,111.28	2229.81	2270.43
E145K, E639K	1914.048	1610.467	− 962.4	− 911.5	− 255.66; 77.64	− 297.20; 0.60	18,020	17,104	37.65	38.23	− 33,608.41	− 34,125.89	2229.17	2260.51
W302G, F400L	1953.466	1614.071	− 963	− 912.1	− 264.70; 80.68	− 276.84; 0.64	17,610	17,498	37.55	38.51	− 33,764.94	− 34,085.03	2238.17	2254.5
T593I, P729S	1914.139	1610.824	− 961.6	− 912.4	− 252.30; 97.07	− 276.30; 0.61	17,564	16,480	41.19	38.23	− 33,833.05	− 34,198.39	2230.24	2253.95
H195Y, F683L	1915.986	1611.376	− 959.4	− 912	− 288.43; 80.40	− 287.87; 1.02	16,346	17,496	35.61	38.23	− 33,689.66	− 34,089.83	2233.06	2282.74
K131Q, F683L	1926.192	1607.986	− 960.6	− 911.6	− 271.10; 59.53	− 258.83; 0.58	16,920	17,002	37.65	38.23	− 33,594.54	− 34,122.19	2231.31	2234.06
L120I, V658L	1948.539	1611.715	− 961.4	− 912.1	− 270.33; 69.18	− 271.47; 0.58	18,634	16,988	39.33	38.51	− 33,539.64	− 34,168.34	2229.56	2255.49
S280Y, Q598H	1915.337	1829.162	− 961.6	− 912.4	− 281.25; 81.24	− 286.14; 0.80	16,434	16,262	41.19	38.23	− 33,816.57	− 34,014.4	2202.54	2250.29
D427N, A576T	1939.018	1762.417	− 991.7	− 889	− 278.57; 33.81	− 278.53; 81.75	18,594	16,734	38.39	38.23	− 33,566.21	− 34,155.13	2231.09	2275.95
W48L, N437H	1915.488	1609.783	− 960.5	− 911.6	− 277.97; 76.40	− 296.34; 0.59	18,644	16,966	37.65	38.51	− 33,781.09	− 34,033.35	2235.91	2217.02
P336S, K26N	1926.076	1622.009	− 960.7	− 912.1	− 271.62; 0.74	− 302.06; 0.57	17,116	17,038	41.19	38.23	− 33,574.55	− 34,185.19	2231.95	2276.9
N578S, Y497C	1926.296	1608.942	− 961.3	− 911.6	− 279.88; 39.17	− 311.50; 91.44	18,028	17,068	37.65	38.23	− 33,612.68	− 34,196.69	2227.28	2251.72
P178S, E182D, D427N	1916.756	1612.884	− 960.4	− 911.5	− 273.19; 96.28	− 279.02; 0.48	17,998	17,476	41.19	38.23	− 33,614.94	− 34,148.23	2229.39	2221.69
R169I, H195Y, N394H	1927.957	1609.288	− 959.5	− 912.3	− 258.06; 0.40	− 306.88; 0.61	18,296	16,970	41.19	38.23	− 33,605.83	− 34,088.23	2230.57	2243.68
N194K, R306I, E479D	1951.544	1613.413	− 963.8	− 912.2	− 262.26; 88.15	− 298.21; 0.53	16,912	17,596	41.19	38.23	− 33,596.17	− 34,160.76	2227	2267.12
S128I, R169I, S602Y	1915.23	1610.646	− 960.4	− 911.6	− 260.69; 91.30	− 261.59; 84.10	16,030	17,072	37.65	38.23	− 33,616.46	− 34,115.39	2227.3	2258.81
M82T, F314L, K600N	1916.484	1610.808	− 836.5	− 840.6	− 278.14; 112.77	− 298.11; 81.57	16,258	17,308	41.19	38.23	− 33,664.9	− 34,228.11	2228.7	2243.8
E375D, K577N, R768W	1926.215	1608.922	− 960.6	− 911.6	− 285.27; 77.81	− 281.60; 0.50	17,990	17,116	39.33	38.23	− 33,569.43	− 34,130.63	2229.86	2268.41
Q18K, G268C, W610L	1914.101	1610.911	− 961.7	− 911.6	− 263.89; 1.21	− 279.45; 0.53	15,788	17,820	39.33	38.23	− 33,829.06	− 34,179.68	2230.49	2256.97
A25V, A396T, I679N	1948.531	1614.638	− 961	− 912.2	− 266.51; 0.57	− 271.92; 0.62	17,798	16,224	40.1	38.23	− 33,556.52	− 34,121.5	2231.71	2284.86
R393I, E571G, R768W	1916.232	1607.701	− 963.1	− 912.3	− 269.93; 31.88	− 307.73; 0.54	17,656	15,940	41.19	38.23	− 33,785.09	− 33,995.76	2230.73	2252.96

Structure-based docking score of **ZDOCK** identified the ACE2 twenty-five single mutants, L8F, T20I, V59D, F72C, P138S, R204I, G205V, R219P, R219H, A264S, D269N, S317F, Q325P, D355N, M383I, D494G, R644Q, I694M, P737L, L760M, R775I, P780S, D785N, G789R, and T803I and two multiple mutants E145K/E639K, and Q18K/G268C/W610L compared to wild-type have lower binding affinity for SARS-CoV. However, mutant Q325P possessed the lowest binding affinity for SARS-CoV.

Likewise, lower binding affinity compared to wild-type has been identified for SARS-CoV-2 by fifty-eight single mutants, T20I, E22D, H34N, E35K, E37K, F72C, L73S, R115W, P138S, E182D, G211W, I256M, A264S, D269N, D269Y, G272C, R273K, V293I, Q305L, S317F, N330H, G337E, N338D, D355N, D368N, M383I, R393G, A403V, E406K, E489K, D494G, T496A, K577N, P590L, N599K, Y613H, K625T, R644Q, E667K, K676E, F683L, I694M, E701K, R708Q, R716H, R716C, N720S, P737H, I761T, F762L, R766K, R768W, R775I, P780S, D785N, G789R, T798P, T803I, and eleven multiple mutants, E145K/E639K, T593I/P729S, K131Q/F683L, W48L/N437H, N578S/Y497C, R169I/H195Y/N394H, S128I/R169I/S602Y, M82T/F314L/K600N, E375D/K577N/R768W, Q18K/G268C/W610L, and R393I/E571G/R768W. Nevertheless, mutant R768W has the lowest binding affinity for SARS-CoV-2.

Oppositely, higher binding affinity compared to wild-type was predicted for SARS-CoV by one-hundred seven single mutants, E22D, F28L, H34N, E35K, E37K, L39M, S44L, L73S, A99S, S109L, R115Q, R115W, L116F, G147V, L162F, E182D, E189K, H195Y, Y202H, D206Y, G211W, V212I, D213G, G220C, E232K, A242T, I256M, D269Y, G272C, R273K, S280Y, V293I, A296T, Q305L, A311V, L320F, T324S, N330H, T334R, G337E, N338D, G352W, R357S, I358F, V364A, D367V, D368N, R393G, G395V, E398K, G399R, A403V, G405W, E406K, S409L, A412T, K419T, P426L, P426S, D431G, L450P, K458T, M462I, I468T, W473L, W477R, V488M, E489K, V491L, T496A, R518M, G561R, P565L, K577N, V581I, P590L, D597E, N599K, K600N, N601I, D609N, Y613H, D615Y, I618M, K625T, L628F, E667K, V670L, V672A, K676E, F683L, S692F, D693N, E701K, R708Q, D713N, R716H, R716C, N720S, P737H, V748F, I761T, F762L, G764R, R766K, R768L and R768W, and nineteen multiple mutants, S47C/P284S, W302G/F400L, T593I/P729S, H195Y/F683L, K131Q/F683L, L120I/V658L, S280Y/Q598H, D427N/A576T, W48L/N437H, P336S/K26N, N578S/Y497C, P178S/E182D/D427N, R169I/H195Y/N394H, N194K/R306I/E479D, S128I/R169I/S602Y, M82T/F314L/K600N, E375D/K577N/R768W, A25V/A396T/I679N and R393I/E571G/R768W. Amidst all ACE2 mutants, the single mutant V748F demonstrated the highest binding affinity with SARS-CoV.

Also, the higher binding affinity was identified compared to wild-type for SARS-CoV-2 by seventy-six single mutants, L8F, F28L, L39M, S44L, V59D, A99S, S109L, R115Q, L116F, G147V, L162F, E189K, H195Y, Y202H, R204I, G205V, D206Y, V212I, D213G, R219P, R219H, G220C, E232K, A242T, S280Y, A296T, A311V, L320F, T324S, Q325P, T334R, G352W, R357S, I358F, V364A, D367V, G395V, E398K, G399R, G405W, S409L, A412T, K419T, P426L, P426S, D431G, L450P, K458T, M462I, I468T, W473L, W477R, V488M, V491L, R518M, G561R, P565L, M579T, V581I, D597E, K600N, N601I, D609N, D615Y, I618M, L628F, V670L, V672A, S692F, D693N, D713N, P737L, V748F, L760M, G764R and R768L, and ten multiple mutants, S47C/P284S, W302G/F400L, H195Y/F683L, L120I/V658L, S280Y/Q598H, D427N/A576T, P336S/K26N, P178S/E182D/D427N, N194K/R306I/E479D and A25V/A396T/I679N. Nonetheless, the single mutant S280Y demonstrated the highest binding affinity with SARS-CoV-2.

However, neutral (no change in binding affinity, their score is similar to wild type. Hence, we are considering that they will not change the binding affinity with SARS-CoV or SARS-CoV-2 and remains as No-Inhibition) was noted with wild-type SARS-CoV for two ACE2 single mutants, M579T and T798P. In contrast, no-neutral affinity was identified for SARS-CoV-2 in ZDOCK score.

**ClusPro** based on docking score compared to wild-type complex identified lower binding affinity for SARS-CoV by seventy ACE2 single mutants, H34N, E35K, E37K, L39M, F72C, L73S, A99S, R115W, L116F, P138S, E189K, H195Y, R204I, G205V, I256M, D269N, D269Y, R273K, S280Y, V293I, A296T, Q305L, A311V, L320F, T324S, G337E, N338D, D355N, I358F, V364A, D368N, M383I, R393G, G395V, E398K, A403V, E406K, S409L, P426L, P426S, D431G, I468T, W473L, W477R, E489K, D494G, T496A, P565L, K577N, M579T, V581I, P590L, N601I, Y613H, K625T, E667K, V672A, K676E, S692F, I694M, E701K, R708Q, R716H, R716C, N720S, V748F, L760M, I761T, F762L, R768L, and fifteen multiple mutants, S47C/P284S, T593I/P729S, H195Y/F683L, K131Q/F683L, L120I/V658L, S280Y/Q598H, W48L/N437H, P336S/K26N, N578S/Y497C, P178S/E182D/D427N, R169I/H195Y/N394H, S128I/R169I/S602Y, M82T/F314L/K600N, E375D/K577N/R768W and A25V/A396T/I679N.

Similarly, lower binding affinity compared to wild-type was recognized for SARS-CoV-2 by fifty-one single mutants, F28L, L39M, S44L, V59D, F72C, A99S, L116F, P138S, G147V, E182D, R204I, G205V, D213G, R219P, R219H, G220C, D269N, D269Y, G272C, V293I, A296T, S317F, Q325P, G337E, N338D, G352W, R357S, M383I, G399R, G405W, P426L, V488M, G561R, P565L, V581I, N599K, D609N, I618M, K625T, L628F, V672A, I694M, R716C, N720S, P737H, P737L, V748F, I761T, F762L, G764R and R766K, and five multiple mutants, S47C/P284S, E145K/E639K, D427N/A576T, P178S/E182D/D427N, and M82T/F314L/K600N. A multiple mutant, M82T/F314L/K600N for SARS-CoV, identified the lowest binding affinity, while a single mutant R357Sfor SARS-CoV-2.

Contrariwise, higher binding affinity with SARS-CoV was noted by fifty-three ACE2 single mutants, E22D, F28L, S44L, V59D, S109L, R115Q, G147V, L162F, E182D, Y202H, D206Y, G211W, D213G, R219P, R219H, G220C, E232K, A242T, A264S, G272C, S317F, Q325P, T334R, G352W, R357S, D367V, G399R, G405W, A412T, K419T, L450P, K458T, M462I, V488M, V491L, R518M, G561R, D597E, N599K, K600N, D615Y, I618M, L628F, R644Q, V670L, F683L, D693N, D713N, P737H, P737L, G764R, R766K, R768W, and five multiple mutants, E145K/E639K, W302G/F400L, D427N/A576T, N194K/R306I/E479D and R393I/E571G/R768W.

Also, the higher binding affinity for SARS-CoV-2 was identified by fifty-four ACE2 single mutants, T20I, E22D, E35K, L73S, S109L, R115Q, L162F, E189K, H195Y, Y202H, D206Y, G211W, V212I, E232K, A242T, A264S, S280Y, Q305L, A311V, L320F, T334R, I358F, D367V, R393G, G395V, E398K, E406K, S409L, A412T, K419T, D431G, L450P, K458T, M462I, W473L, W477R, V491L, T496A, R518M, K577N, D597E, K600N, D615Y, E667K, V670L, K676E, F683L, S692F, D693N, E701K, D713N, L760M, R768L and R768W, and ten multiple mutants, W302G/F400L, T593I/P729S, H195Y/F683L, L120I/V658L, S280Y/Q598H, P336S/K26N, R169I/H195Y/N394H, N194K/R306I/E479D, A25V/A396T/I679N and R393I/E571G/R768W.

A single mutant P737H and E22Dpossessed the highest binding affinity with SARS-CoV, and SARS-CoV-2, respectively. However, neutral binding affinity was noted for SARS-CoV by eleven ACE2 single mutants, L8F, T20I, V212I, N330H, D609N, R775I, P780S, D785N, G789R, T798P, and T803I, and one multiple mutant Q18K/G268C/W610L. Also, the neutral binding affinity for SARS-CoV-2 was identified by twenty-nine single mutants, L8F, H34N, E37K, R115W, I256M, R273K, T324S, N330H, D355N, V364A, D368N, A403V, P426S, I468T, E489K, D494G, M579T, P590L, N601I, Y613H, R644Q, R708Q, R716H, R775I, P780S, D785N, G789R, T798P and T803I, and six multiple mutants K131Q/F683L, W48L/N437H, N578S/Y497C, S128/R169I/S602Y, E375D/K577N/R768W, and Q18K/G268C/W610L.

The **HDOCK** docking score predicted the lower binding affinity for SARS-CoV by ACE2 thirty-nine single mutants, E22D, H34N, L39M, V59D, F72C, L73S, A99S, Y202H, G205V, V212I, R219H, E232K, A242T, D269Y, Q325P, N338D, R357S, I358F, D368N, E406K, S409L, K419T, M462I, V488M, E489K, V491L, T496A, P565L, M579T, V581I, D597E, D609N, D615Y, K625T, R708Q, N720S, V748F, I761T and R768L, and five multiple mutants, E145K/E639K, T593I/P729S, R169I/H195Y/N394H, N194K/R306I/E479D, and S128I/R169I/S602Y.

Likewise, lower binding affinity was identified for SARS-CoV-2by ACE2 twenty-nine single mutants, E22D, F28L, L39M, S44L, V59D, S109L, R115Q, R115W, P138S, G205V, D206Y, G211W, V212I, D269N, V293I, N330H, G352W, R357S, R393G, G395V, P426L, I468T, D494G, P565L, Y613H, K625T, V670L, N720S and V748F, and eight multiple mutants W302G/F400L, T593I/P729S, K131Q/F683L, L120I/V658L, D427N/A576T, P178S/E182D/D427N, S128I/R169I/S602Y and A25V/A396T/I679N.

Nevertheless, ACE2 mutant, M579T, and K131Q/F683L compared to wild-type have the lowest binding affinity with SARS-CoV, and SARS-CoV-2, respectively.

In contrast, higher binding affinity for SARS-CoV was identified by eighty-seven single mutants, F28L, E35K, E37K, S44L, S109L, R115Q, R115W, L116F, P138S, G147V, L162F, E18 2D, E189K, H195Y, R204I, D206Y, G211W, D213G, R219P, G220C, I256M, A264S, D269N, G272C, R273K, S280Y, V293I, A296T, Q305L, A311V, S317F, L320F, T324S, N330H, T334R, G337E, G352W, D355N, V364A, D367V, M383I, R393G, G395V, E398K, G399R, A403V, G405W, A412T, P426L, P426S, D431G, L450P, K458T, I468T, W473L, W477R, D494G, R518M, G561R, K577N, P590L, N599K, K600N, N601I, Y613H, I618M, L628F, R644Q, E667K, V670L, V672A, K676E, F683L, S692F, D693N, I694M, E701K, D713N, R716H, R716C, P737H, P737L, L760M, F762L, G764R, R766K and R768W, and fifteen multiple mutants, S47C/P284S, W302G/F400L, H195Y/F683L, K131Q/F683L, L120I/V658L, S280Y/Q598H, D427N/A576T, W48L/N437H, P336S/K26N, N578S/Y497C, P178S/E182D/D427N, M82T/F314L/K600N, E375D/K577N/R768W, A25V/A396T/I679N and R393I/E571G/R768W.

Likewise, SARS-CoV-2 has increased binding affinity for ninety-seven single mutants, H34N, E35K, E37K, F72C, L73S, A99S, L116F, G147V, L162F, E182D, E189K, H195Y, Y202H, R204I, D213G, R219P, R219H, G220C, E232K, A242T, I256M, A264S, D269Y, G272C, R273K, S280Y, A296T, Q305L, A311V, S317F, L320F, T324S, Q325P, T334R, G337E, N338D, D355N, I358F, V364A, D367V, D368N, M383I, E398K, G399R, A403V, G405W, E406K, S409L, A412T, K419T, P426S, D431G, L450P, K458T, M462I, W473L, W477R, V488M, E489K, V491L, T496A, R518M, G561R, K577N, M579T, V581I, P590L, D597E, N599K, K600N, N601I, D609N, D615Y, I618M, L628F, R644Q, E667K, V672A, K676E, F683L, S692F, D693N, I694M, E701K, R708Q, D713N, R716H, R716C, P737H, P737L, L760M, I761T, F762L, G764R, R766K, R768L and R768W, and twelve multiple mutants, S47C/P284S, E145K/E639K, H195Y/F683L, S280Y/Q598H, W48L/N437H, P336S/K26N, N578S/Y497C, R169I/H195Y/N394H, N194K/R306I/E479D, M82T/F314L/K600N, E375D/K577N/R768W and R393I/E571G/R768W.

Nonetheless, among all ACE2 mutants, a single mutant D713N displayed the highest binding affinity for both SARS-CoV and SARS-CoV-2. Moreover, neutral binding affinity was noted for both SARS-CoV and SARS-CoV-2 compared to wild-type by eight single mutants, L8F, T20I, R775I, P780S, D785N, G789R, T798P, T803I, and one multiple mutant Q18K/G268C/W610L.

Furthermore, no lower binding affinity mutant was identified using **PatchDock,** but it recognized higher binding affinity compared to wild-type ACE2 for SARS-CoV with one hundred twenty six mutants, E22D, F28L, H34N, E35K, E37K, L39M, S44L, V59D, F72C, L73S, A99S, S109L, R115Q, R115W, L116F, P138S, G147V, L162F, E182D, E189K, H195Y, Y202H, R204I, G205V, D206Y, G211W, V212I, D213G, R219P, R219H, G220C, E232K, A242T, I256M, A264S, D269N, D269Y, G272C, R273K, S280Y, V293I, A296T, Q305L, A311V, S317F, L320F, T324S, Q325P, N330H, T334R, G337E, N338D, G352W, D355N, R357S, I358F, V364A, D367V, D368N, M383I, R393G, G395V, E398K, G399R, A403V, G405W, E406K, S409L, A412T, K419T, P426L, P426S, D431G, L450P, K458T, M462I, I468T, W473L, W477R, V488M, E489K, V491L, D494G, T496A, R518M, G561R, P565L, K577N, M579T, V581I, P590L, D597E, N599K, K600N, N601I, D609N, Y613H, D615Y, I618M, K625T, L628F, R644Q, E667K, V670L, V672A, K676E, F683L, S692F, D693N, I694M, E701K, R708Q, D713N, R716H, R716C, N720S, P737H, P737L, V748F, L760M, I761T, F762L, G764R, R766K, R768L and R768W, and **twenty** multiple mutants, S47C/P284S, E145K/E639K, W302G/F400L, T593I/P729S, H195Y/F683L, K131Q/F683L, L120I/V658L, S280Y/Q598H, D427N/A576T, W48L/N437H, P336S/K26N, N578S/Y497C, P178S/E182D/D427N, R169I/H195Y/N394H, N194K/R306I/E479D, S128I/R169I/S602Y, M82T/F314L/K600N, E375D/K577N/R768W, A25V/A396T/I679N and R393I/E571G/R768W. However, a single mutant, P737L, has the highest binding affinity with SARS-CoV.

Inversely, lower binding affinity for SARS-CoV-2 was identified with one hundred eighteen single mutants, E22D, F28L, H34N, E35K, E37K, L39M, S44L, V59D, L73S, A99S, S109L, R115Q, R115W, L116F, G147V, L162F, E182D, E189K, H195Y, Y202H, R204I, G205V, D206Y, G211W, V212I, D213G, R219P, R219H, G220C, E232K, A242T, I256M, A264S, D269N, D269Y, G272C, R273K, S280Y, V293I, A296T, Q305L, A311V, S317F, L320F, T324S, Q325P, N330H, T334R, G337E, N338D, G352W, D355N, R357S, I358F, V364A, D367V, D368N, M383I, R393G, G395V, E398K, G399R, A403V, G405W, E406K, A412T, K419T, P426L, P426S, L450P, K458T, M462I, I468T, W473L, W477R, V488M, E489K, V491L, D494G, T496A, R518M, G561R, P565L, K577N, M579T, V581I, P590L, N599K, K600N, N601I, Y613H, D615Y, K625T, L628F, R644Q, E667K, V670L, V672A, K676E, F683L, S692F, D693N, E701K, R708Q, D713N, R716H, R716C, N720S, P737H, P737L, V748F, L760M, I761T, F762L, G764R, R766K, R768L, R768W, and twenty multiple mutants, S47C/P284S, E145K/E639K, W302G/F400L, T593I/P729S, H195Y/F683L, K131Q/F683L, L120I/V658L, S280Y/Q598H, D427N/A576T, W48L/N437H, P336S/K26N, N578S/Y497C, P178S/E182D/D427N, R169I/H195Y/N394H, N194K/R306I/E479D, S128I/R169I/S602Y, M82T/F314L/K600N, E375D/K577N/R768W, A25V/A396T/I679N and R393I/E571G/R768W.

Nevertheless, a multiple mutant, S47C/P284S, attained the lowest binding affinity with SARS-CoV-2. In contrast, six single mutants, D597E, D609N, F72C, S409L, I618M, and D431G, have a higher binding affinity with SARS-CoV-2, whileD597E identified the highest binding affinity.

Also, the neutral binding affinity compared to wild-type was noted for SARS-CoV by eight ACE2 single mutants, L8F, T20I, R775I, P780S, D785N, G789R, T798P, T803I, and one multiple mutant Q18K/G268C/W610L. However, ten single mutants, L8F, T20I, P138S, I694M, R775I, P780S, D785N, G789R, T798P, T803I, and one multiple mutant Q18K/G268C/W610L were identified to have a neutral binding affinity with SARS-CoV-2.

**InterEvDock2** docking score identified lower binding affinity for SARS-CoV by fifty-eight single ACE2 mutants, E22D, H34N, E35K, E37K, V59D, S109L, G147V, E189K, H195Y, Y202H, R204I, G205V, D213G, R219P, R219H, G220C, E232K, A264S, D269Y, R273K, Q305L, Q325P, N330H, R357S, V364A, R393G, E398K, E406K, K419T, P426S, D431G, L450P, K458T, I468T, W477R, E489K, R518M, K577N, M579T, P590L, D597E, K600N, D609N, I618M, K676E, F683L, S692F, D693N, I694M, E701K, R708Q, N720S, P737H, P737L, V748F, L760M, G764R and R768W, and nine multiple mutants, S47C/P284S, E145K/E639K, W302G/F400L, H195Y/F683L, K131Q/F683L, D427N/A576T, W48L/N437H, N578S/Y497C, and S128I/R169I/S602Y.

Similarly, lower binding affinity was identified for SARS-CoV-2 with twenty-two single mutants, S109L, R115W, L116F, R204I, G205V, D206Y, G211W, R219P, R219H, S317F, T334R, G337E, G352W, E398K, K419T, D431G, V488M, L628F, R716C, P737H, I761T, R766K.

However, mutant G220C showed the lowest binding affinity for SARS-CoV and I761T for SARS-CoV-2.

Conversely, the higher binding affinity was perceived with a similar docking score for SARS-CoV by sixty-three ACE2 single mutants, F28L, L39M, S44L, F72C, L73S, A99S, R115Q, R115W, L116F, P138S, L162F, E182D, D206Y, G211W, V212I, A242T, D269N, G272C, S280Y, V293I, A296T, A311V, S317F, L320F, T324S, G337E, N338D, G352W, I358F, D367V, D368N, G395V, G399R, A403V, G405W, S409L, A412T, P426L, M462I, W473L, V488M, V491L, D494G, T496A, G561R, P565L, V581I, N599K, Y613H, D615Y, K625T, L628F, R644Q, E667K, V670L, V672A, D713N, R716H, R716C, I761T, F762L, R766K and R768L, and nine multiple mutants, T593I/P729S, S280Y/Q598H, P336S/K26N, P178S/E182D/D427N, R169I/H195Y/N394H, N194K/R306I/E479D, M82T/F314L/K600N, A25V/A396T/I679N and R393I/E571G/R768W.

Also, higher binding affinity was perceived for SARS-CoV-2 with twenty-one single mutants, F28L, H34N, E37K, L39M, F72C, D213G, D269N, G272C, Q305L, Q325P, M383I, R393G, V491L, T496A, D615Y, S692F, V748F, F762L, G764R, R768L and R768W, and three multiple mutants, W302G/F400L, L120I/V658L, and W48L/N437H.

Although, mutant G561R and R768W attained the highest binding affinity for SARS-CoV and SARS-CoV-2, respectively.

Moreover, compared to wild-type neutral binding affinity was noted for SARS-CoV by thirteen ACE2 single mutants, L8F, T20I, I256M, T334R, D355N, M383I, N601I, R775I, P780S, D785N, G789R, T798P, T803I, and three multiple mutants L120I/V658L, E375D/K577N/R768W, and Q18K/G268C/W610L.

Also, the neutral binding affinity for SARS-CoV-2 was predicted by ninety-one single mutants, L8F, T20I, E22D, E35K, S44L, V59D, L73S, A99S, R115Q, P138S, G147V, L162F, E182D, E189K, H195Y, Y202H, V212I, G220C, E232K, A242T, I256M, A264S, D269Y, R273K, S280Y, V293I, A296T, A311V, L320F, T324S, N330H, N338D, D355N, R357S, I358F, V364A, D367V, D368N, G395V, G399R, A403V, G405W, E406K, S409L, A412T, P426L, P426S, L450P, K458T, M462I, I468T, W473L, W477R, E489K, D494G, R518M, G561R, P565L, K577N, M579T, V581I, P590L, D597E, N599K, K600N, N601I, D609N, Y613H, I618M, K625T, R644Q, E667K, V670L, V672A, K676E, F683L, D693N, I694M, E701K, R708Q, D713N, R716H, N720S, P737L, L760M, R775I, P780S, D785N, G789R, T798P and T803I, and eighteen multiple mutants S47C/P284S, E145K/E639K, T593I/P729S, H195Y/F683L, K131Q/F683L, S280Y/Q598H, D427N/A576T, P336S/K26N, N578S/Y497C, P178S/E182D/D427N, R169I/H195Y/N394H, N194K/R306I/E479D, S128I/R169I/S602Y, M82T/F314L/K600N, E375D/K577N/R768W, Q18K/G268C/W610L, A25V/A396T/I679N and R393I/E571G/R768W.

**SOAP-PP** envisaged the lower binding affinity compared to wild-type for SARS-CoV by one-hundred seventeen single mutants, E22D, F28L, H34N, E37K, L39M, S44L, V59D, F72C, L73S, A99S, S109L, R115Q, R115W, L116F, P138S, G147V, L162F, E182D, E189K, H195Y, Y202H, G205V, D206Y, G211W, V212I, D213G, G220C, E232K, A242T, I256M, A264S, D269N, D269Y, G272C, R273K, S280Y, V293I, A296T, Q305L, A311V, S317F, L320F, T324S, Q325P, N330H, T334R, G337E, N338D, G352W, D355N, R357S, I358F, V364A, D367V, D368N, M383I, R393G, G395V, E398K, G399R, A403V, G405W, E406K, S409L, A412T, K419T, P426L, P426S, D431G, L450P, K458T, M462I, I468T, W473L, W477R, E489K, V491L, D494G, T496A, R518M, G561R, P565L, K577N, M579T, V581I, P590L, D597E, N599K, K600N, N601I, D609N, Y613H, D615Y, I618M, K625T, L628F, R644Q, E667K, V670L, V672A, K676E, F683L, S692F, D693N, E701K, R708Q, D713N, R716H, R716C, N720S, P737L, L760M, I761T, F762L, G764R, R766K and R768L, and nineteen multiple mutants, S47C/P284S, E145K/E639K, W302G/F400L, H195Y/F683L, K131Q/F683L, L120I/V658L, S280Y/Q598H, D427N/A576T, W48L/N437H, P336S/K26N, N578S/Y497C, P178S/E182D/D427N, R169I/H195Y/N394H, N194K/R306I/E479D, S128I/R169I/S602Y, M82T/F314L/K600N, E375D/K577N/R768W, A25V/A396T/I679N and R393I/E571G/R768W.

As well, lower binding affinity was achieved for SARS-CoV-2 by one-hundred five single mutants, F28L, E35K, L39M, S44L, V59D, F72C, L73S, A99S, S109L, R115Q, R115W, L116F, P138S, G147V, L162F, E182D, H195Y, Y202H, R204I, G205V, D206Y, G211W, D213G, R219P, R219H, E232K, A242T, A264S, D269N, D269Y, R273K, S280Y, A296T, Q305L, A311V, S317F, L320F, T324S, N330H, T334R, G337E, N338D, G352W, D355N, R357S, I358F, V364A, D367V, D368N, M383I, R393G, G395V, G399R, A403V, G405W, E406K, S409L, A412T, P426L, P426S, D431G, L450P, K458T, M462I, I468T, W473L, W477R, V488M, D494G, R518M, G561R, P565L, M579T, V581I, P590L, N599K, K600N, N601I, D609N, Y613H, D615Y, I618M, K625T, L628F, R644Q, E667K, V670L, V672A, K676E, F683L, S692F, I694M, E701K, R708Q, D713N, R716H, R716C, N720S, P737L, V748F, I761T, F762L, R766K, R768L and R768W, and sixteen multiple mutants, S47C/P284S, E145K/E639K, W302G/F400L, H195Y/F683L, K131Q/F683L, L120I/V658L, S280Y/Q598H, D427N/A576T, W48L/N437H, P178S/E182D/D427N, R169I/H195Y/N394H, N194K/R306I/E479D, S128I/R169I/S602Y, E375D/K577N/R768W, A25V/A396T/I679N and R393I/E571G/R768W.

Moreover, a single mutant G764R has the lowest binding affinity for SARS-CoV, whereas L628F for SARS-CoV-2. Oppositely, higher binding affinity with SARS-CoV was identified by ten single mutants, E35K, R204I, R219H, R219P, V488M, I694M, P737H, V748F, R768W and P780S, and one multiple mutant T593I/P729S.

Congruently, higher binding affinity was identified for SARS-CoV-2 by twenty-one single mutants, E22D, H34N, E37K, E189K, V212I, G220C, I256M, G272C, V293I, Q325P, E398K, K419T, E489K, V491L, T496A, K577N, D597E, D693N, P737H, L760M and G764R, and four multiple mutants, T593I/P729S, P336S/K26N, N578S/Y497C, and M82T/F314L/K600N. The highest binding affinity was identified for SARS-CoV and SARS-CoV-2 by R219H and Q325P, respectively.

Moreover, neutral binding affinity compared to wild-type was noted for SARS-CoV by seven ACE2 single mutants, L8F, T20I, R775I, D785N, G789R, T798P, T803I, and one multiple mutant, Q18K/G268C/W610L. Conversely, eight single mutants L8F, T20I, R775I, P780S, D785N, G789R, T798P, T803I, and one multiple mutant Q18K/G268C/W610L showed neutral binding affinity for SARS-CoV-2.

**FRODOCK2** identified the lower binding affinity compared to wild-type for SARS-CoV by eighty-eight single mutants, E22D, F28L, E35K, E37K, L39M, S44L, V59D, F72C, L73S, A99S, R115Q, L116F, P138S, G147V, E182D, E189K, Y202H, R204I, D213G, R219P, G220C, E232K, A242T, I256M, D269N, D269Y, R273K, S280Y, V293I, A296T, Q305L, A311V, S317F, T324S, Q325P, N330H, T334R, G337E, N338D, G352W, D355N, R357S, I358F, D367V, M383I, R393G, G395V, G405W, E406K, S409L, A412T, P426L, P426S, D431G, L450P, K458T, M462I, I468T, W473L, W477R, E489K, V491L, G561R, P565L, K577N, M579T, V581I, D597E, N601I, D609N, Y613H, D615Y, I618M, K625T, R644Q, E667K, V670L, V672A, S692F, I694M, R708Q, R716H, R716C, N720S, I761T, F762L, R766K and R768L, and eleven multiple mutants, S47C/P284S, E145K/E639K, T593I/P729S, L120I/V658L, S280Y/Q598H, N578S/Y497C, P178S/E182D/D427N, N194K/R306I/E479D, S128I/R169I/S602Y, M82T/F314L/K600N, and E375D/K577N/R768W.

Nevertheless, decreased binding affinity was identified for SARS-CoV-2 by sixty-five single mutants, E22D, F28L, H34N, S44L, V59D, F72C, L73S, R115Q, R115W, L116F, G147V, E182D, R204I, G205V, G211W, D213G, R219H, R219P, G220C, A242T, A264S, G272C, S280Y, V293I, A296T, Q325P, N330H, G352W, D355N, R357S, I358F, M383I, R393G, G395V, G399R, E406K, S409L, A412T, K419T, P426L, D431G, L450P, M462I, W473L, D494G, T496A, P565L, K577N, M579T, P590L, D597E, D609N, D615Y, I618M, K625T, R644Q, E667K, V670L, S692F, I694M, P737H, V748F, I761T, G764R and R768W, and eleven multiple mutants, W302G/F400L, T593I/P729S, K131Q/F683L, L120I/V658L, S280Y/Q598H, W48L/N437H, N578S/Y497C, P178S/E182D/D427N, R169I/H195Y/N394H, M82T/F314L/K600N and R393I/E571G/R768W.

However, single mutant G561R and R219H attained the lowest binding affinity for SARS-CoV and SARS-CoV-2, respectively.

Oppositely, higher binding affinity was identified for SARS-CoV with thirty-seven single ACE2 mutants, H34N, S109L, R115W, L162F, H195Y, G205V, D206Y, G211W, V212I, R219H, G272C, L320F, V364A, D368N, E398K, G399R, A403V, K419T, V488M, D494G, T496A, R518M, P590L, N599K, K600N, L628F, K676E, F683L, D693N, E701K, D713N, P737H, P737L, V748F, L760M, G764R and R768W, and nine multiple mutants, W302G/F400L, H195Y/F683L, K131Q/F683L, D427N/A576T, W48L/N437H, P336S/K26N, R169I/H195Y/N394H, A25V/A396T/I679N and R393I/E571G/R768W.

Also, a higher binding affinity was obtained for SARS-CoV-2 by sixty-one single mutants, E35K, E37K, L39M, A99S, S109L, P138S, L162F, E189K, H195Y, Y202H, D206Y, V212I, E232K, I256M, D269N, D269Y, R273K, Q305L, A311V, S317F, L320F, T324S, T334R, G337E, N338D, V364A, D367V, D368N, E398K, A403V, G405W, P426S, K458T, I468T, W477R, V488M, E489K, V491L, R518M, G561R, V581I, N599K, K600N, N601I, Y613H, L628F, V672A, K676E, F683L, D693N, E701K, R708Q, D713N, R716H, R716C, N720S, P737L, L760M, F762L, R766K and R768L, and nine multiple mutants, S47C/P284S, E145K/E639K, H195Y/F683L, D427N/A576T, P336S/K26N, N194K/R306I/E479D, S128I/R169I/S602Y, E375D/K577N/R768W and A25V/A396T/I679N.

Nonetheless, single mutant P737H and D367 Vattained the highest binding affinity for SARS-CoV and SARS-CoV-2, respectively.

Moreover, neutral binding affinity compared to wild-type was noted for SARS-CoV by nine ACE2 single mutants, L8F, T20I, A264S, R775I, P780S, D785N, G789R, T798P, T803I, and one multiple mutant Q18K/G268C/W610L. Contrariwise, eight single mutants, L8F, T20I, R775I, P780S, D785N, G789R, T798P, T803I, and one multiple mutant Q18K/G268C/W610L possessed neutral binding affinity with SARS-CoV-2.

#### Cell line ACE2 mutations affect the binding affinity with SARS-CoV and SARS-CoV-2

25 ACE2 mutants, namely S5F, A25V, L100V, V184A, S218N, Y252C, P253T, T276K, N322I, T334A, A413V, K416N, P426L, E457K, Q472P, P612L, W635L, Y649C, E668K and A782V single mutants, and A386T/F314I, F603C/K619N, F314L/Y510H, L664I/D382Y and E145K/E495K/I233S multiple mutants, were recognized across the cell lines and their binding affinity with SARS-CoV and SARS-CoV-2 was compared by employing various structure-based docking tools, such as ZDOCK, ClusPro, HDOCK, PatchDock, InterEvDock2, SOAP-PP and FRODOCK2 (Table 9). This comparative analysis was performed to identify the common mutants and hotspots that can be correlated with the findings of the patient data.

**Table 9:** Docking between cell lines’ ACE2 mutants with SARS-CoV, and SARS-CoV-2 respectively.

Mutants	ZDOCK (score)		ClusPro (score)		HDOCK (score; ligand rmsd (Å))		PatchDock (score)		InterEvDock2 (score)		SOAP-PP (score)		FRODOCK2 (score)	
	SARS-CoV	SARS-CoV-2	SARS-CoV	SARS-CoV-2	SARS-CoV	SARS-CoV-2	SARS-CoV	SARS-CoV-2	SARS-CoV	SARS-CoV-2	SARS-CoV	SARS-CoV-2	SARS-CoV	SARS-CoV-2
WILD TYPE	1914.101	1610.912	− 961.7	− 911.6	− 263.89; 1.21	− 279.45; 0.53	15,788	17,820	39.33	38.23	− 33,829.06	− 34,179.68	2230.49	2256.97
S5F	1914.105	1610.915	− 961.7	− 911.6	− 263.89; 1.21	− 279.45; 0.53	15,788	17,820	39.33	38.23	− 33,829.06	− 34,179.68	2230.49	2256.97
A25V	1948.538	1614.639	− 961	− 912.2	− 266.51; 0.57	− 271.92; 0.62	17,798	16,224	40.1	38.23	− 33,556.52	− 34,121.5	2231.71	2284.86
L100V	1915.179	1610.814	− 960.5	− 911.5	− 273.01; 64.87	− 309.90; 0.44	17,460	16,484	41.19	38.23	− 33,601.01	− 34,165.93	2229.14	2248.93
V184A	1915.355	1610.811	− 960.4	− 911.6	− 282.43; 76.54	− 310.18; 0.72	18,630	16,288	41.19	38.23	− 33,812.15	− 34,193.58	2228.73	2252.45
S218N	1913.991	1610.933	− 960.5	− 911.5	− 269.87; 79.42	− 311.69; 0.40	18,962	18,658	41.19	38.23	− 33,811.63	− 34,157.25	2231.67	2259.33
Y252C	1916.617	1610.815	− 960.5	− 911.5	− 267.41; 101.87	− 294.81; 99.36	17,442	17,508	40.1	38.23	− 33,536.72	− 34,039.13	2234.27	2283.54
P253T	1915.14	1611.784	− 961.4	− 912.1	− 288.09; 80.34	− 285.12; 0.59	18,296	17,058	41.19	38.51	− 33,649.74	− 34,097.14	2230.23	2266.46
T276K	1915.093	1612.653	− 961.6	− 911.5	− 275.21; 103.65	− 296.87; 0.54	18,650	17,252	41.19	38.23	− 33,590.61	− 34,044.33	2228.97	2257.21
N322I	1916.252	1610.375	− 959.4	− 911.3	− 291.13; 0.56	− 280.04; 0.45	18,970	16,532	38.69	38.23	− 33,515.28	− 34,131.95	2229.96	2267.33
T334A	1914.28	1608.709	− 960.4	− 911.6	− 277.53; 80.87	− 302.67; 0.51	15,784	16,810	41.19	38.23	− 33,586.11	− 34,153.92	2234.79	2246.87
A413V	1916.654	1612.122	− 960.4	− 911.5	− 263.87; 78.38	− 289.63; 0.86	17,436	16,834	41.19	38.23	− 33,580.21	− 34,104.45	2228.75	2265.76
K416N	1916.378	1613.509	− 960.8	− 912.1	− 266.97; 68.34	289.20; 83.62	16,498	17,556	37.76	38.23	− 33,562.37	− 34,164.42	2227.29	2267.41
P426L	1916.609	1617.803	− 960.4	− 911.5	− 268.99; 1.03	− 260.65; 0.48	16,662	17,060	41.19	38.23	− 33,569.35	− 34,150.06	2228.79	2256.23
E457K	1926.044	1622.106	− 962	− 913	− 276.75; 87.84	− 276.01; 0.71	17,924	16,110	41.19	38.23	− 33,577.57	− 34,183.14	2231.74	2281.22
Q472P	1927.365	1621.275	− 961.2	− 913.2	− 282.73; 76.16	− 286.13; 70.39	19,336	17,142	41.19	38.23	− 33,590.52	− 34,111.18	2224.35	2230.82
P612L	1939.588	1620.767	− 961.2	− 914.1	− 280.28; 81.88	− 281.54; 0.56	19,084	16,508	41.19	38.23	− 33,569.42	− 34,195.99	2239.2	2240.68
W635L	1939.542	1624.261	− 960.9	− 911.2	− 277.96; 0.52	− 283.60; 0.58	15,862	16,954	41.19	37.69	− 33,576.92	− 34,038.2	2196.7	2261.93
Y649C	1925.991	1608.861	− 960.7	− 911.6	− 274.58; 104.34	− 285.54; 0.60	17,994	17,116	42.6	37.69	− 33,486.96	− 33,997.68	2232	2203.72
E668K	1914.003	1610.443	− 962.4	− 912.1	− 269.71; 0.50	− 270.00; 80.96	16,694	16,208	38.69	38.23	− 33,609.29	− 34,196.35	2229.56	2216.36
A782V	1914.104	1610.91	− 961.7	− 911.6	− 263.89; 1.21	− 279.45; 0.53	15,788	17,820	39.33	38.23	− 33,829.06	− 34,179.68	2230.49	2256.97
A386T, F314I	1915.177	1610.875	− 960.4	− 911.5	− 286.82; 78.66	− 281.98; 0.57	16,190	16,260	41.19	38.23	− 33,587.64	− 34,067.24	2229.14	2252.37
F603C, K619N	1913.976	1610.919	− 960.4	− 911.5	− 284.55; 32.61	− 288.71; 0.72	18,640	18,524	40.1	35.9	− 33,445.31	− 34,075.45	2223.64	2227.82
F314L, Y510H	1926.083	1609.74	− 961.7	− 912	− 308.01; 61.14	− 295.32; 0.37	16,684	17,294	41.19	38.23	− 33,628	− 34,174.61	2230.13	2258.27
L664I, D382Y	1915.238	1609.81	− 960.7	− 911.6	− 286.78; 77.92	− 279.75; 0.47	17,950	16,624	41.19	38.51	− 33,636.99	− 34,193.47	2229.77	2216.54
E145K, E495K, I233S	1914.049	1610.463	− 962.4	− 911.5	− 255.66; 77.64	− 297.20; 0.60	18,020	17,104	37.65	38.23	− 33,608.41	− 34,125.89	2229.17	2260.51

**ZDOCK** docking score identified three single ACE2 mutants, S218N, E668K, F603C, and K619N, and two multiple mutants, F603C/K619N and E145K/E495K/I233S, that have a decreased binding affinity for SARS-CoV, with the lowest score obtained for multiple mutant F603C/K619N. Likewise, ten mutants were spotted, including L100V, V184A, Y252C, N322I, Y649C, E668K, A782V, F314L, Y510H, L664I, D382Y and T334A, and two multiple mutants, E145K/E495K/I233S and A386T/F314I with a lower binding affinity for SARS-CoV-2. The least score was observed in the T334A mutant.

Conversely, higher binding affinity for SARS-CoV-2 was identified for 19 single mutants, S5F, L100V, V184A, Y252C, P253T, T276K, N322I, T334A, A413V, K416N, P426L, E457K, Q472P, P612L, W635L, Y649C, A782V, L664I, D382Y and A25V, and two multiple mutants A386T/F314I and F314L/Y510H with the highest affinity recognized for A25V. Similarly, 12 single mutants, S5F, A25V, S218N, P253T, T276K, A413V, K416N, P426L, E457K, Q472P, P612L and W635L, and multiple mutants, F603C/K619N obtained scores greater than wild type ACE2, suggesting an increased affinity for SARS-CoV-2 binding, the maximum score seen in W635L.

**ClusPro** scores predicted decreased affinity for SARS-CoV in 16 single mutants, N322I, A25V, L100V, V184A, S218N, Y252C, P253T, T276K, T334A, A413V, K416N, P426L, Q472P, P612L, W635L and Y649C, and three multiple mutants, A386T/F314I, F603C/K619N, and L664I/D382Y. Additionally, eight single mutants, W635L, L100V, S218N, Y252C, T276K, N322I, A413V and P426L, and three multiple mutants, A386T/F314I, F603C/K619N and E145K/E495K/I233S, were also identified with a decreased binding affinity towards SARS-CoV-2. Single mutants N322I and W635L had the lowest binding scores towards SARS-CoV and SARS-CoV-2, respectively.

However, an enhanced affinity for SARS-CoV was observed in only two single mutants, E457K and E668K, and the multiple mutants, E145K/E495K/I233S. Similarly, seven single mutants, A25V, P253T, K416N, E457K, Q472P, P612L and E668K, and multiple mutants, F314L/Y510H, were recognized to possess a greater affinity to bind with SARS-CoV-2. The highest score to bind with SARS-CoV was attained by E668K and E145K/E495K/I233S, and that with SARS-CoV-2 seen in P612L.

On the other hand, three mutants, S5F, A782V, and F314L/Y510H, and six mutants, S5F, V184A, T334A, Y649C, A782V and L664I/D382Y, disclosed a neutral binding affinity towards SARS-CoV and SARS-CoV-2, respectively.

Based on the **HDOCK** scoring, the scores for mutants S5F and A782V were the same as that for wild-type ACE2, pointing towards a neutral binding affinity change for both SARS-CoV and SARS-CoV-2.

Nevertheless, a decreased affinity for SARS-CoV was revealed in the case of E145K/E495K/I233S and A413V, the lowest being for the multiple mutant E145K/E495K/I233S. Concerning SARS-CoV-2 binding, a similar trend was observed with only four single mutants (A25V, E457K, E668K, and P426L) attaining a lower affinity than the control score, but the most negligible affinity seen in P426L.

A higher affinity towards SARS-CoV was noted in 17 single mutants, A25V, L100V, V184A, S218N, Y252C, P253T, T276K, N322I, T334A, K416N, P426L, E457K, Q472P, P612L, W635L, Y649C and E668K, and four multiple mutants, A386T/F314I, F603C/K619N, L664I/D382Y and F314L/Y510H, the highest observed in F314L/Y510H multiple mutants. Likewise, 14 single mutants, L100V, V184A, S218N, Y252C, P253T, T276K, N322I, T334A, A413V, K416N, Q472P, P612L, W635L and Y649C, and five multiple mutants, A386T/F314I, F603C/K619N, F314L/Y510H, L664I/D382Y, and E145K/E495K/I233S were perceived with an increased binding affinity towards SARS-CoV-2, the highest score noted for S218N.

**PatchDock** scores bracketed S5F and A782V with a neutral binding affinity towards both SARS-CoV and SARS-CoV-2.

Among the 25 mutants, T334A had a lower binding affinity value towards SARS-CoV than the control. The other 22 mutants, A25V, L100V, V184A, S218N, Y252C, P253T, T276K, N322I, A413V, K416N, P426L, E457K, P612L, W635L, Y649C, E668K and Q472P, including multiple mutants, A386T/F314I, F603C/K619N, F314L/Y510H, L664I/D382Y, and E145K/E495K/I233S, verified to have increased affinity for SARS-CoV binding, highest observed for Q472P.

Whereas, comparing the ACE2 mutants binding with SARS-CoV-2, 17 single mutants, E457K, A25V, L100V, V184A, Y252C, P253T, T276K, N322I, T334A, A413V, K416N, P426L, Q472P, P612L, W635L, Y649C, and E668K, along with four multiple mutants, A386T/F314I, F314L/Y510H, L664I/D382Y and E145K/E495K/I233S, displayed a decreased score as compared to the control. 2 mutants yet, S218N and F603C/K619N, were identified with an increased affinity. The most negligible and highest affinity with SARS-CoV-2 was identified in E457K and S218N, respectively.

**InterEvDock2** scores illustrated three mutants N322I, K416N and E668K, and a multiple mutant E145K/E495K/I233S with a decreased binding affinity towards SARS-CoV. E145K/E495K/I233S mutant exhibited the most negligible affinity among all the others identified. F603C/K619N, W635L, and Y649C mutants were noted with a decreased binding affinity towards SARS-CoV-2, F603C/K619N exhibiting the minimum score.

In context to the mutants with an enhanced binding affinity identified by InterEvDock2 docking, 15 single mutants A25V, L100V, V184A, S218N, Y252C, P253T, T276K, T334A, A413V, P426L, E457K, Q472P, P612L, W635L and Y649C, and four multiple mutants A386T/F314I, F603C/K619N, F314L/Y510H and L664I/D382Y were recognized for SARS-CoV binding. Amongst these, Y649C possessed the highest binding affinity values.

Whereas only P253T and L664I/D382Y were recognized with a value greater than a control for SARS-CoV-2 binding, the scores being numerically the same.

Interestingly, S5F and A782V single mutants exhibited a neutral binding affinity with SARS-CoV, while as many as 17 single mutants A25V, L100V, V184A, S218N, Y252C, T276K, N322I, T334A, A413V, K416N, P426L, E457K, Q472P, P612L, E668K and A782V, and three multiple mutants, A386T/F314I, F314L/Y510H and E145K/E495K/I233S attained the binding affinity score same as that of the wild-type ACE2.

**SOAP-PP** docking scores verified S5F and A782V with no change in binding towards both SARS-CoV and SARS-CoV-2.

Out of the other 23 mutants, 18 single mutants, A25V, L100V, V184A, S218N, Y252C, P253T, T276K, N322I, T334A, A413V, K416N, P426L, E457K, Q472P, P612L, W635L, Y649C and E668K, and five multiple mutants, A386T/F314I, F314L/Y510H, L664I/D382Y, E145K/E495K/I233S and F603C/K619N, achieved a lower binding affinity score with SARS-CoV, the lowest score predicted for multiple mutant F603C/K619N and no mutant with a score more significant than the control.

Regarding the SARS-CoV-2 binding, 14 single mutants Y649C, A25V, L100V, S218N, Y252C, P253T, T276K, N322I, T334A, A413V, K416N, P426L, Q472P and W635L, and four multiple mutants A386T/F314I, F603C/K619N, F314L/Y510H and E145K/E495K/I233S, certified a reduced binding affinity, the lowest value of F603C/K619N mutant. However, four single mutants V184A, E457K, P612L and E668K, and multiple mutants, L664I/D382Y, enhanced binding affinity, the highest discerned in E668K.

Like the SOAP-PP scoring, FRODOCK2 scores also selected S5F and A782V to have a neutral binding affinity towards SARS-CoV and SARS-CoV-2.

Nonetheless, **FRODOCK2** also perceived 11 single mutants W635L, L100V, V184A, P253T, T276K, N322I, A413V, K416N, P426L, Q472P and E668K, and five multiple mutants, A386T/F314I, F603C/K619N, F314L/Y510H, L664I/D382Y and E145K/E495K/I233S with a lower binding affinity towards SARS-CoV. For SARS-CoV-2 binding, eight single mutants L100V, V184A, T334A, P426L, Q472P, Y649C, P612L, and E668K, and three multiple mutants, A386T/F314I, F603C/K619N, and L664I/D382Y, were encountered with reduced binding affinity. The lowest values in both SARS-CoV and SARS-CoV-2 cases were observed for W635L and Y649C, respectively.

A greater binding towards SARS-CoV was observed for seven mutants, namely A25V, S218N, Y252C, T334A, E457K, P612L, and Y649C. Likewise, ten single mutants, A25V, S218N, 252C, P253T, T276K, N322I, A413V, K416N, E457K and W635L, and two multiple mutants, F314L/Y510H and E145K/E495K/I233S displayed a higher binding affinity than wild type ACE2 for SARS-CoV-2 binding. The maximum scores for SARS-CoV and SARS-CoV-2 binding were observed in the case of P612L and A25V.

### Machine learning prediction classifies the interaction disrupting mutations

**Figure 3:**
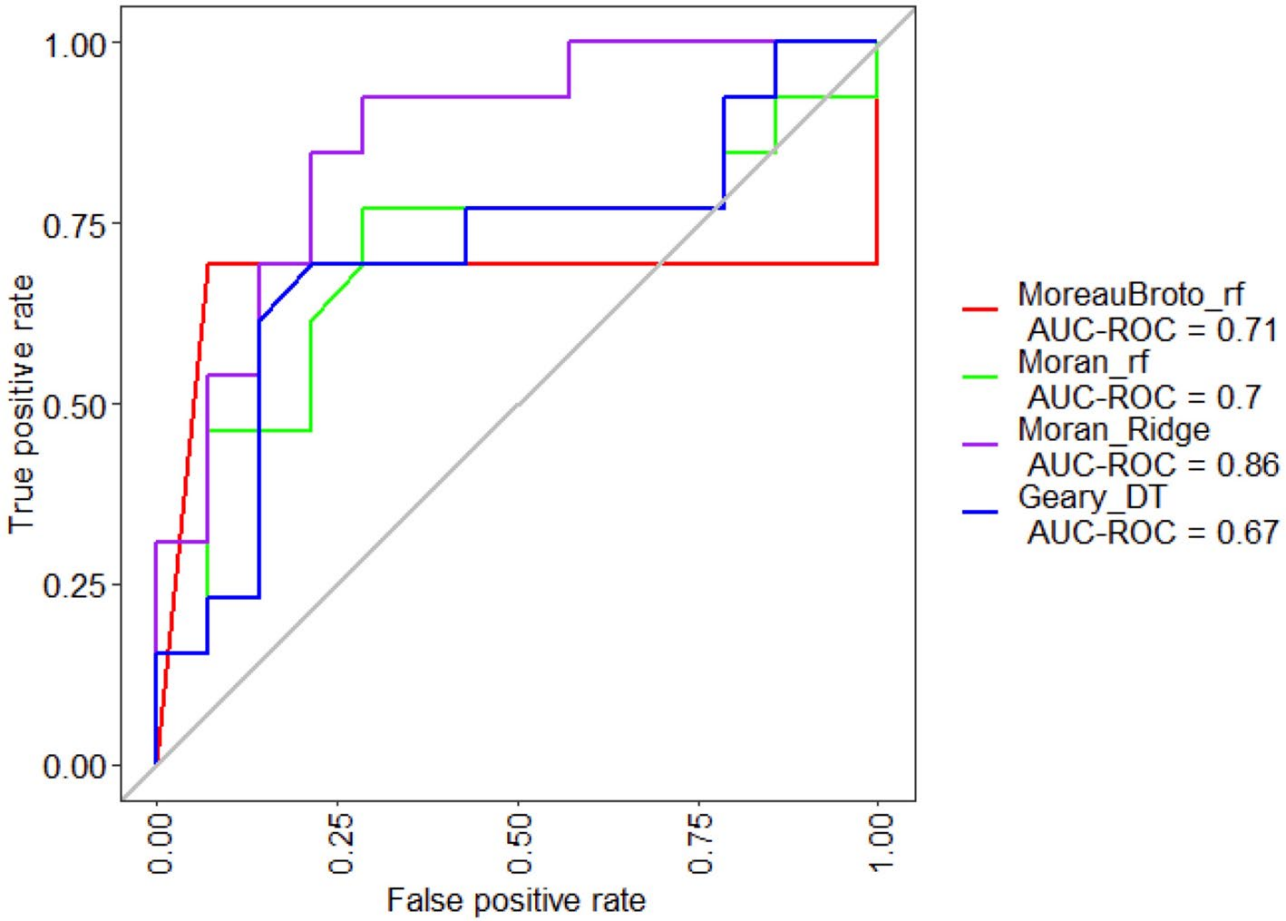
ROC curves for the best performing machine learning-based classifiers.

We developed various machine learning models based on different algorithms such as, DT, RF, KNN, MLP, Ridge, Lasso, ElasticNet, and NeuralNet. The 33 sequences were split into training and testing datasets in 80:20 ratio. Multiple features have been generated using the “protr” package, and these features were tested separately for their classification strength among the protein classes. It was found that models employing Autocorrelation descriptors—MoreauBroto, Moran, and Geary performed well with balanced results in terms of accuracy, sensitivity, specificity, and AUC compared to others. Ridge-based model based on Moran descriptor gave the best-balanced results on training (accuracy: 77.78%, AUC: 0.78) and testing (accuracy: 66.67%, AUC: 0.67) data. Table 10 depicts statistical details of the evaluation parameters of best performed classifiers developed using autocorrelation descriptors. Receiver operating characteristic (ROC) curves of the best four models are shown in Fig. 3. We performed the prediction on 155 patient samples containing single and multiple mutations using our RIDGE-based Moran Classifier at a strict threshold of 0.9. Prediction results reflect that out of 155, only 18 have no impact on the binding with SARS-CoV-2, whereas remaining 137 show abolished binding to the SARS-Cov-2 (Supplementary Table 1). We also validated our RIDGE-based Moran classifier on cell line data with 25 mutants at a similar threshold of 0.9. Our model predicted three mutants with no impact on binding, and remaining 22 were predicted to abolish the binding to the SARS-Cov-2 (Supplementary Table 2).

**Table 10:** Evaluation parameters of top performed classifiers developed using autocorrelation descriptors.

Classifier	Sensitivity (%)	Specificity (%)	Accuracy (%)	MCC	AUC
**Training**					
MoreauBroto (RF)	69.23	71.43	70.37	0.40	0.70
Moran (RF)	76.90	71.40	74.07	0.48	0.74
Moran (Ridge)	76.92	78.57	77.78	0.55	0.78
Geary (DT)	69.33	78.57	74.07	0.48	0.74
**Testing**					
MoreauBroto (RF)	66.66	66.66	66.66	0.33	0.66
Moran (RF)	66.66	66.66	66.66	0.33	0.66
Moran (Ridge)	66.67	66.67	66.67	0.34	0.67
Geary (DT)	66.66	66.66	66.66	0.33	0.66

### ACE2 expression and survival analysis in different cancer types

**Figure 4:**
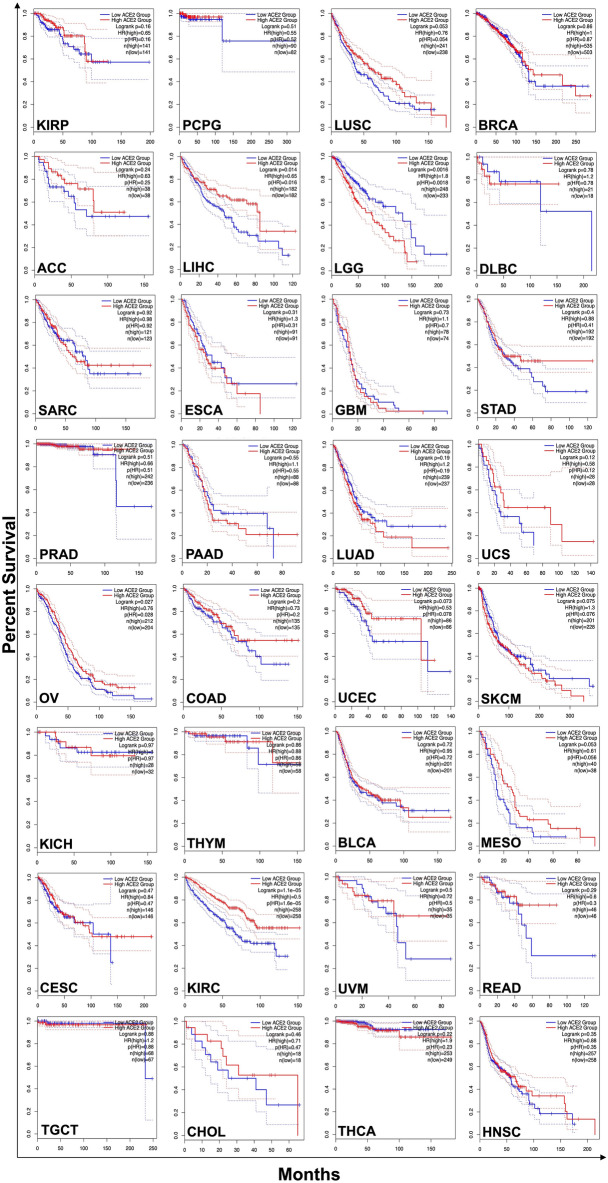
Overall survival analysis was performed using the GEPIA platform. The solid line represents the survival curve and the dotted line represents the 95% confidence interval. Log‐rank p < 0.05 was considered to indicate a statistically significant difference. Patients with expression above the median are indicated by red lines, and patients with expression below the median are indicated by blue lines. Significantly, higher ACE2 expression level was positively associated with overall survival of patients in LIHC (p = 0.014), OV (p = 0.027) and KIRC (p = 1.1e−05) but obtained negatively correlated with overall survival LGG (p = 0.0016). *ACE2* angiotensin-converting enzyme 2, *HR* hazard ratio, *TMP* transcripts per million, *ACC* adrenocortical carcinoma, *BLCA* bladder urothelial carcinoma, *BRCA* breast invasive carcinoma, *CESC* cervical squamous cell carcinoma and endocervical adenocarcinoma, *CHOL* cholangio carcinoma, *COAD* colon adenocarcinoma, *DLBC* lymphoid neoplasm diffuse large B-cell lymphoma, *ESCA* esophageal carcinoma, *GBM* glioblastoma multiforme, *HNSC* head and neck squamous cell carcinoma, *KICH* kidney chromophobe, *KIRC* kidney renal clear cell carcinoma, *KIRP* kidney renal papillary cell carcinoma, *LAML* acute myeloid leukemia, *LGG* brain lower grade glioma, *LIHC* liver hepatocellular carcinoma, *LUAD* lung adenocarcinoma, *LUSC* lung squamous cell carcinoma, *MESO* mesothelioma, *OV* ovarian serous cystadenocarcinoma, *PAAD* pancreatic adenocarcinoma, *PCPG* pheochromocytoma and paraganglioma, *PRAD* prostate adenocarcinoma, *READ* rectum adenocarcinoma, *SARC* sarcoma, *SKCM* skin cutaneous melanoma, *STAD* stomach adenocarcinoma, *TGCT* testicular germ cell tumors, *THCA* thyroid carcinoma, *THYM* thymoma, *UCEC* uterine corpus endometrial carcinoma, *UCS* uterine carcinosarcoma, *UVM* uveal melanoma.

Overall survival analysis of the ACE2 was performed using GEPIA plotters. Survival analyses were performed to assess the significant (p < 0.05) association of the ACE2 gene with the overall survival of the patients in different cancer tissue types (Fig. 4). It demonstrates that high expression of ACE2 is positively linked to the overall survival of the patients in KIRP, PCPG, LUSC, ACC, LIHC, SARC, STAD, PRAD, UCS, OV, COAD, UCEC, THYM, BLCA, MESO, CESC, KIRC, UVM, READ, CHOL, and HNSC. However, an opposite trend can be seen in the cancers like LGG, DLBC, ESCA, GBM, PAAD, LUAD, SKCM, and TGCT, i.e., low expression levels of ACE2 in these cancers are directly correlated with the overall survival of the patients. We have also found that ACE2 expression in LIHC (p = 0.014), LGG (p = 0.0016), OV (p = 0.027), and KIRC (p = 1.1e−05) cancers were significantly associated with the overall survival of the patients. Among the significant ones, the Log-rank test (p < 0.05) and the survival curves indicated that higher ACE2 expression in LIHC, OV, and KIRC were positively associated with overall survival with a Hazard Ratio (HR) of 0.65, 0.76, and 0.5, respectively. However, the higher expression of ACE2 in LGG was negatively associated with the overall survival of the patients with an HR of 1.8. The HR indicates the fold risk associated with one subgroup compared to the other. For example, an HR of 1.8 in the LGG cohort depicts that the group with low expression of ACE2 is at 1.8 fold lower risk of death than the group with high expression of ACE2.

**Figure 5:**
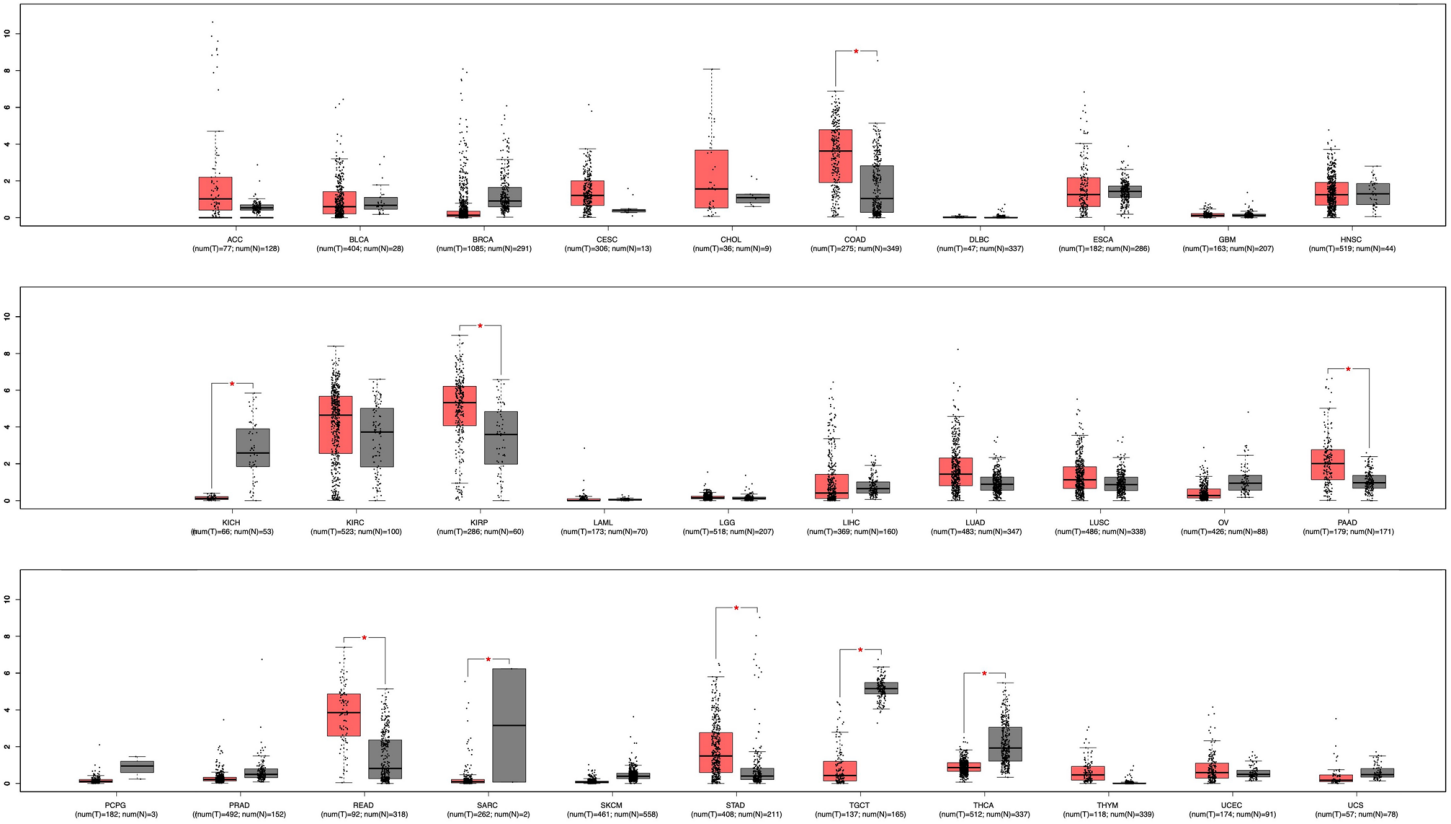
The boxplots represent the comparison of downregulated and upregulated expression of ACE2 gene in normal, N (gray), and tumor T (red) tissue types.

The expression level of the ACE2 gene in different cancer types was analyzed (Fig. 5). Compared with healthy controls, ACE2 was downregulated in BRCA, KICH, OV, PCPG, PRAD, SARC, SKCM, TGCT, THCA, and UCS, wherein downregulated ACE2 expression was significantly obtained in KICH (p < 0.05), SARC (p < 0.05), TGCT (p < 0.05), and THCA (p < 0.05).

Nevertheless, ACE2 gene expression was upregulated in THYM, ACC, BLCA, CESC, CHOL, COAD, ESCA, HNSC, KIRC, KIRP, LIHC, LUAD, LUSC, PAAD, READ, STAD, and UCEC, in which upregulated significant ACE2 gene expression was observed in COAD (p < 0.05), KIRP (p < 0.05), PAAD (p < 0.05), READ (p < 0.05), and STAD (p < 0.05).

**Figure 6:**
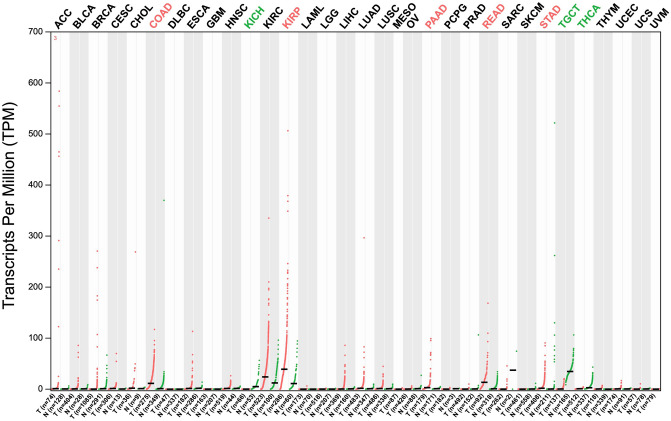
Dot plot of ACE2 gene expression profile across different tumor samples and paired normal tissues. Each dot represents sample expression; red denotes tumor samples and green denotes normal samples. *TPM* transcripts per million, *T* tumor, *N* normal.

The upregulation of ACE2 was the most significantly associated with overall survival of KIRC patients (log-rank p = 1.1e−05; Fig. 4) with an HR of 0.5 which suggests that patients with high expression of ACE2 are nearly 0.5 folds lower risk of death as compared with the patients having increased expression of ACE2. Also, ACE2 downregulation was significantly associated in KICH (p < 0.05), SARC (p < 0.05), and TGCT (p < 0.05) cohorts (Fig. 5). Further analysis revealed that ACE2 expression was lower in TGCT than normal tissue (Fig. 6).

## Discussion

Identifying the mutants serving as possible cancer hotspots and those with an affinity greater than control to bind with the SARS-CoV and SARS-CoV-2, a comparative analysis was performed between impact prediction scores data and the docking studies prediction scores to ascertain the plausibly deleterious mutations. An overall inference score was calculated for each mutant’s binding affinity with SARS-CoV and SARS-CoV-2, establishing an overall decrease or increase in the affinity with respective virus strains. This score was later matched up with the inference scores of the impact prediction studies, calculated similarly to enlist the high-risk mutants in both scenarios. The number of deleterious mutants identified by the different impact prediction tools varies.

Out of the total 32 mutants in the experimental data, PROVEAN recognized 13 mutants with deleterious impact on the protein functionality, whereas Mutation Assessor, SIFT, and PolyPhen-2 listed 4, 8, and 9 high-risk mutants, respectively. Among the 26 single mutants enlisted in the experimental data, P389A, D350A and D355A identified to be the highest risk mutation by all four tools, Mutation Assessor, SIFT, PROVEAN, and PolyPhen-2. R357A, M383A, and R393A are expected to be high-risk mutations based on SIFT, PROVEAN, and PolyPhen-2 tool impact scores. D38A mutant displayed the low-risk scores by all the impact prediction tools, suggesting that it does not significantly affect the protein functionality. Oppositely, the multi-mutants had tolerated risk scores across all four software, considered to have a benign impact on protein functionality. Only D136M in the multi mutant P135S/D136M and G466D in K465Q/G466D/E467K exhibited a deleterious effect based on the PROVEAN impact prediction. Subsequently, the comparison and identification of cancer hotspots with their binding affinity with SARS-CoV and SARS-CoV-2 were performed.

Contrarywise this, the analysis of patient samples data revealed that SIFT predicted the highest number of high-risk mutants, with 89 out of 155 considered the deleterious impact on the proteins’ functionality. PolyPhen-2 and PROVEAN indicated 52 and 51 mutants to have a high-risk impact on tumorigenesis. Nevertheless, as observed in cell line and experimental data analysis, Mutation Assessor was not limited in assessing the patient samples, yet outlined only 24 mutants with high-risk impact while exhibiting medium risk scores for as many as 80 mutants. Moreover, 20 single mutants, F72C, L162F, Y202H, R204I, S317F, D355N, R357S, I358F, R393G, G395V, G399R, A403V, G405W, L450P, W477R, G561R, P565L, M579T, P590L, and N599K and two multi-mutants W302G/F400L and W48L/N437H, were detected by all four prediction tools to exhibit damaging and deleterious influence in tumor development. This predisposition might make the individual carrying these mutants more prone or resistant to COVID-19 infection with either of these two strains.

Likewise, out of the 25 mutants associated with the cell line data, SIFT and PROVEAN impact scores exhibited as many as 15 and 14 deleterious mutants, respectively. However, Mutation Assessor and PolyPhen-2 impact scores showed only 3 and 7 mutants, respectively. All four tools predicted three single mutants, V184A, Y252C, and P612L, with high impact scores, inferring their deleterious and probably damaging effect on tumor progression. Nevertheless, Mutation Assessor predicted the least number of high-risk mutants. Therefore, based on SIFT, PolyPhen-2, and PROVEAN impact score, single mutants, E457K, W635L and Y649C, and a multi-mutant F314L/Y510H was predicted to have damaging ramifications on proteins’ functions. Henceforth, those mutants categorized with high-risk impact prediction scores were matched with their affinity to bind with SARS-CoV and SARS-CoV-2.

Thus, to summarize, PROVEAN identified the highest number of mutants posing as cancer hotspots in the experimental data, while SIFT score revealed this trend in cell line data and patient samples. However, the Mutation Assessor tool could not analyze most of the mutants within the experimental data due to its limitations. Paradox prediction was observed in many mutants; thus, an overall inference scoring facilitated identifying high-risk mutants with functional damaging effects and a greater tendency to bind with either or both SARS-CoV and SARS-C0V-2.

The correlation between experimental and docked mutant impact with SARS-CoV and SARS-CoV-2 has been discussed (Supplementary Table 3). The docking was performed to examine the impact of the interaction between ACE2 mutants with SARS-CoV and SARS-CoV-2. The docking results suggest that association and dissociation of ACE2 interaction with SARS-CoV and SARS-CoV-2 involved switching cancer functions by activating downstream signaling. The ACE2 mutants, having predicted lower binding affinity with SARS-CoV or SARS-CoV-2revealed that these mutants induce structural changes in ACE2, which leads to the disrupted binding. Oppositely, the mutants wherein no inhibition suggest that the ACE2 mutants have increased or a neutral impact on binding with SARS-CoV or SARS-CoV-2. All structure‐based docking tools used to dock ACE2 mutants with SARS-CoV displayed inconsistencies. So, in this study, the docking results of patient data correlated with the experimental validated binding impact have been discussed. Primarily, the common binding affinity of the mutants showing inhibition and no-inhibition of interaction identified using docking tools with both SARS-CoV and SARS-CoV-2 has been discussed for experimental cell lines and patient samples. In the experimental samples, ZDOCK indicates 22 common mutants having an inhibitory and non-inhibitory effect with both SARS-CoV and SARS-CoV-2, wherein, the inhibitory effect was possessed by five mutants (K31D, Y41A, T324A, R559S, and F603T), while 17 mutants (E37A, D38A, E110P, E160R, H239Q, D350A, K353H, K353A, K353D, D355A, R357A, L359K, P389A, Q24K/A25A/K26E, M82N/Y83F/P84S, S425P/P426S/D427N, and K465Q/G466D/E467K) have a non-inhibitory effect. ClusPro implies 13 common mutants having an inhibitory and non-inhibitory effect with both SARS-CoV and SARS-CoV-2, wherein, the inhibitory effect was possessed by five mutants (Y41A, K68D, D350A, K353D, and P389A), while nine mutants (E37A, D38A, E110P, E160R, K309D, E312A, R357A, L359A, and K465Q/G466D/E467K) have a non-inhibitory effect. HDOCK designates 18 common mutants having an inhibitory and non-inhibitory effect with both SARS-CoV and SARS-CoV-2, wherein, the inhibitory effect was possessed by one mutant (E160R), while 17 mutants (D38A, E110P, R192D, R219D, H239Q, K309D, T324A, K353H, K353A, K353D, D355A, R357A, M383A, P389A, R393A, R559S, S425P/P426S/D427N) have a non-inhibitory effect. PatchDock indicates two common mutants having an inhibitory and non-inhibitory effect with both SARS-CoV and SARS-CoV-2, wherein, **no** mutant has an inhibitory effect. In comparison, two mutants (Y41A and D355A) have a non-inhibitory effect. InterEvDock2 indicates 15 common mutants having an inhibitory and non-inhibitory effect with both SARS-CoV and SARS-CoV-2, wherein, the inhibitory effect was possessed by three mutants (R219D, F603T, and N338D/V339D/Q340R), while 12 mutants (K31D, D38A, K68D, E110P, H239Q, K309D, D350A, K353H, K353D, M383A, P389A, and P135S/D136M) have a non-inhibitory effect. **SOAP-PP** indicates 21 common mutants having an inhibitory and non-inhibitory effect with both SARS-CoV and SARS-CoV-2, wherein, the inhibitory effect was possessed by 21 mutants (K31D, E37A, D38A, K68D, E160R, H239Q, K309D, T324A, D350A, K353H, K353A, R357A, L359K, L359A, P389A, R559S, F603T, Q24K/A25A/K26E, M82N/Y83F/P84S, P135S/D136M, and N338D/V339D/Q340R), while no mutant has a non-inhibitory effect. FRODOCK2 indicates 18 common mutants having an inhibitory and non-inhibitory effect with both SARS-CoV and SARS-CoV-2, wherein, the inhibitory effect was possessed by 14 mutants (E110P, E160R, R219D, T324A, K353A, K353D, D355A, L359K, R559S, F603T, Q24K/A25A/K26E, M82N/Y83F/P84S, N338D/V339D/Q340R, and K465Q/G466D/E467K), while four mutants (K353H, L359A, P135S/D136M, and S425P/P426S/D427N) have a non-inhibitory effect. Secondly, among 32 mutants, the interaction of 15 mutants (K31D, Y41A, K68D, K353H, K353A, K353D, D355A, R357A, M383A, P389A, R393A, R559S, Q24K/A25A/K26E, M82N/Y83F/P84S, and S425P/P426S/D427N) was identified inhibited with the SARS-CoV. In comparison, no inhibition was identified for 17 mutants (E37A, D38A, E110P, R192D, R219D, H239Q, K309D, E312A, T324A, D350A, L359K, L359A, F603T, P135S/D136M, N338D/V339D/Q340R, and K465Q/G466D/E467K) in the experimental known studies. The docking studies of SARS-CoV predicted through SOAP-PP signify the 12 mutants having the highest inhibition correlation with experimental validated 15 mutants. In comparison, 2 two mutants have no inhibition correlation with 17 experimentally validated mutants. Similarly, 13 mutants correlated with SARS-CoV-2 inhibition for experimentally validated mutants has been identified by PatchDock, while no mutant has no inhibition correlation with experimentally validated 17 mutants. In contrast, PatchDock identified that all 17 mutants have the highest no-inhibition correlation with experimental validated 17 mutants, which signifies a non-inhibitory effect on SARS-CoV. Likewise, the highest no inhibition impact of 14 mutants on SARS-CoV-2 has been observed by ClusPro. HDOCK shows the no-inhibition correlation of 14 mutants with 17 experimentally validated mutants on SARS-CoV. Interestingly, ClusPro demonstrated the highest cumulative 18 mutants, wherein eight mutants out of 15 have inhibitory, and ten mutants out of 17 have a non-inhibitory effect on interaction with SARS-CoV. Also, 24 out of 32 mutants, in which ten mutants out of 15 and 14 out of 17 have inhibitory and non-inhibitory effects on SARS-CoV-2, respectively. This result signifies that the ClusPro has the highest reliability score over other docking methods. Nonetheless, the hotspot R559S mutant observed corroborated with known experimentally validated results due to its highest inhibition effect on interaction with SARS-CoV identified by ZDOCK, ClusPro, INTEREVDOCK, SOAP-PP, and FRODOCK2. Also, all the docking tools except ZDOCK have identified the highest inhibitory effect on the interaction of triple mutant, M82N/Y83F/P84S with SARS-CoV-2, which signifies the dissociation of binding between them. The mutant D38A demonstrated the highest non-inhibition effect on binding with SARS-CoV experimental result correlated with predicted docking approaches like ZDOCK, CLUSPRO, HDOCK, PatchDock, InterEvDock2, and FRODOCK2. Likewise, the mutants E110P, H239Q, and K309D, have no inhibitory effect on binding with SARS-CoV-2 predicted by five docking tools. These docking results corroborate experimental validated mutants’ impact on inhibition and non-inhibition of interaction with SARS-CoV and SARS-CoV-2. Moreover, ZDOCK identified the mutant R219D with the lowest binding affinity, which signifies that this mutant significantly inhibits its association with SARS-CoV. Although, the lowest binding affinity of multiple mutants, P135S/D136M with SARS-CoV-2, indicates the significant dissociation of their binding. Conversely, the highest binding affinity of multiple mutants, K465Q/G466D/E467K, increases the binding/association with SARS-CoV, though the mutant K309D increases the association with SARS-CoV-2. The ClusPro identified the four hotspot mutants, Y41A, K68D, T324A, and P389A, which disrupt the binding/association with SARS-CoV, but L359K mutant disrupts the association with SARS-CoV-2. However, a multiple mutant M82N/Y83F/P84S and a single mutant L359A imply the highest potential to increase the binding/association with SARS-CoV, and SARS-CoV-2, respectively. The HDOCK docking score exhibits that the Y41A mutant strongly inhibits the association with SARS-CoV, while multiple mutants, P135S/D136M, strongly inhibits the association with SARS-CoV-2. Nonetheless, the R219D mutant exhibits no inhibition on binding with SARS-CoV, whereas R393A has a non-inhibition effect on binding with SARS-CoV-2. PatchDock has identified all mutants, increasing the binding affinity with SARS-CoV, thereby indicating the mutants’ non-inhibiting effect. Also, the Y41A mutant shows the highest binding affinity with SARS-CoV-2. However, mutant F603T shows the lowest binding affinity with SARS-CoV-2. InterEvDock2 exposed a mutant, L359K, and multiple mutants, N338D/V339D/Q340R, which have the highest inhibiting effect with SARS-CoV, and SARS-CoV-2, respectively. In contrast, all mutants have no inhibiting effect on binding with SARS-CoV, while mutants E37A, Y41A, K353A, and R357A, have no inhibiting effect on binding with SARS-CoV-2. SOAP-PP signifies that the ACE2 mutant, P135S/D136M with SARS-CoV, and D355A with SARS-CoV-2, potentially inhibits the binding affinity. Conversely, the R219D mutant exhibits the highest inhibiting effect on SARS-CoV, whereas R192D displays on SARS-CoV-2. Lastly, FRODOCK2 signifies that an ACE2 multiple mutant N338D/V339D/Q340R potentially disrupts the association with SARS-CoV, while R219D inhibits SARS-CoV-2. Nevertheless, Y41A and K353H have increased binding affinity with SARS-CoV, and SARS-CoV-2, which signifies their non-inhibiting effect.

The correlation between experimental and docked patients’ mutants with SARS-CoV and SARS-CoV-2 has been summarized in Supplementary Table 4. No common mutant was observed between experimental and patient samples, but some mutants in experimental and patient samples were found common at specific amino acid positions, and only a change of single amino acid has been noticed. Primarily, discussing the identified common binding affinity using docking tools of mutants showing inhibition and no-inhibition of interaction with SARS-CoV and SARS-CoV-2. The patient mutant samples’ structure-based docking was performed with SARS-CoV and SARS-CoV-2 using ZDOCK. Cumulatively, 94 mutants have common inhibitory and non-inhibitory effects for both SARS-CoV and SARS-CoV-2, wherein 18 were observed to have a common inhibitory effect (G789R, T803I, T20I, R775I, P780S, Q18K/G268C/W610L, D785N, D269N, A264S, E145K/E639K, F72C, S317F, P138S, I694M, D355N, R644Q, D494G, and M383I). However, 76 mutants possessed non-inhibitory effect (S280Y, S280Y/Q598H, V748F, D427N/A576T, D213G, D615Y, N601I, L628F, G561R, R357S, G220C, P565L, L116F, V488M, S44L, D597E, D609N, G764R, G352W, L162F, L450P, G405W, V670L, L320F, I358F, A311V, E398K, R518M, S109L, E189K, K600N, D693N, P336S/K26N, D206Y, K458T, F28L, Y202H, G395V, K419T, A412T, A242T, V491L, G147V, P426L, P426S, I618M, R768L, G399R, V212I, A25V/A396T/I679N, S409L, W302G/F400L, R115Q, A99S, D431G, D367V, S692F, N194K/R306I/E479D, V672A, D713N, A296T, P178S/E182D/D427N, S47C/P284S, V581I, T334R, T324S, L39M, E232K, L120I/V658L, M462I, H195Y/F683L, H195Y, W473L, W477R, I468T, V364A). Similarly, ClusPro signifies 58 common mutants, which have inhibitory and non-inhibitory effects with both SARS-CoV and SARS-CoV-2, wherein, the inhibitory effect was shown by 28 mutants (L39M, F72C, A99S, P138S, V293I, A296T, M383I, P426L, V581I, K625T, V672A, I694M, R716C, N720S, S47C/P284S, P178S/E182D/D427N, L116F, I761T, P565L, R204I, G205V, F762L, M82T/F314L/K600N, D269Y, G337E, N338D, D269N, and V748F). Although, the non-inhibitory effect was observed by 30 mutants (N194K/R306I/E479D, G211W, E232K, F683L, D615Y, L162F, V491L, R393I/E571G/R768W, D367V, R768W, W302G/F400L, Y202H, D597E, D713N, K419T, V670L, L450P, E22D, A264S, R115Q, M462I, T334R, A412T, S109L, D206Y, K458T, R518M, K600N, D693N, and A242T). Likewise, HDOCK suggest 89 common mutants, in which the inhibitory effect for SARS-CoV and SARS-CoV-2 were observed by 12 mutants (P565L, V212I, N720S, L39M, T593I/P729S, G205V, R357S, E22D, K625T, V748F, V59D, and S128I/R169I/S602Y), while, no-inhibition effect was commonly noted by 77 mutants (D713N, G220C, G399R, L628F, W477R, K458T, F683L, E37K, H195Y, H195Y/F683L, L760M, N601I, E375D/K577N/R768W, A311V, K676E, A412T, D367V, S692F, P590L, D693N, L320F, S280Y, S280Y/Q598H, E667K, G561R, R644Q, N578S/Y497C, G147V, R219P, R716H, S317F, G405W, L116F, M82T/F314L/K600N, W48L/N437H, N599K, A403V, E35K, P737H, I256M, E189K, D213G, E182D, D355N, D431G, E398K, T324S, R204I, G764R, R766K, W473L, F762L, G337E, Q305L, P426S, P336S/K26N, I694M, T334R, A264S, S47C/P284S, R716C, L450P, V364A, R518M, R393I/E571G/R768W, A296T, K577N, M383I, L162F, V672A, G272C, R768W, K600N, I618M, E701K, P737L, and R273K). Also, PatchDock indicates non-inhibitory effects with SARS-CoV and SARS-CoV-2 by six mutants (D597E, D609N, F72C, S409L, I618M, and D431G), while no mutant has an inhibitory effect. InterEvDock2 indicates 19 common mutants having an inhibitory and non-inhibitory effect with both SARS-CoV and SARS-CoV-2, wherein, the inhibitory effect was possessed by nine mutants (R204I, R219P, R219H, E398K, K419T, D431G, P737H, G205V, S109L), while ten mutants (F28L, F72C, L39M, D269N, G272C, V491L, T496A, D615Y, F762L, R768L) have a non-inhibitory effect. It has been observed that SOAP-PP predict the maximum number of common 115 mutants that have inhibitory and non-inhibitory effect with SARS-CoV and SARS-CoV-2. The inhibitory effect was noted by 113 mutants (L628F, D609N, R393I/E571G/R768W, N599K, R393G, I618M, D213G, S280Y, S280Y/Q598H, R768L, D431G, S692F, G561R, Y202H, R716C, F72C, W48L/N437H, T334R, D368N, N720S, R357S, F28L, K625T, S409L, D615Y, S317F, D206Y, F683L, R644Q, G395V, W302G/F400L, W473L, R169I/H195Y/N394H, Y613H, M462I, H195Y, H195Y/F683L, D494G, K600N, R273K, W477R, G399R, R766K, E406K, Q305L, N330H, A403V, G405W, D367V, V672A, A99S, A296T, G352W, S47C/P284S, R708Q, A412T, L320F, A264S, S128I/R169I/S602Y, G337E, V670L, D355N, G211W, K676E, A25V/A396T/I679N, E667K, K131Q/F683L, I358F, A311V, D269N, E145K/E639K, R115W, L116F, S44L, V59D, E375D/K577N/R768W, G147V, P565L, K458T, L162F, V364A, P590L, N601I, P178S/E182D/D427N, E701K, P426L, D269Y, P138S, R115Q, D427N/A576T, I761T, G205V, L450P, E182D, N194K/R306I/E479D, F762L, I468T, P426S, L39M, A242T, E232K, P737L, L120I/V658L, D713N, M383I, L73S, V581I, S109L, N338D, T324S, M579T, R518M, and R716H), while 2 mutants (T593I/P729S, P737H) have non-inhibitory effect with SARS-CoV and SARS-CoV-2. Finally, FRODOCK2 reveals 80 mutants with inhibitory and non-inhibitory effects with SARS-CoV and SARS-CoV-2. The 55 mutants noticed the inhibitory effect (V293I, K625T, P426L, D355N, M383I, L120I/V658L, E406K, S692F, T593I/P729S, M579T, D597E, I694M, W473L, A412T, N578S/Y497C, K577N, A296T, S280Y, S280Y/Q598H, Q325P, I618M, F28L, E182D, P565L, V59D, I761T, M82T/F314L/K600N, E22D, D609N, N330H, D615Y, I358F, G220C, D213G, M462I, A242T, L450P, L73S, S409L, G395V, D431G, L116F, V670L, P178S/E182D/D427N, R644Q, G147V, E667K, R115Q, S44L, R357S, G352W, R393G, F72C, R219P, and R204I), whereas, the non-inhibitory effect was observed for 25 mutants (V488M, P737L, L628F, N599K, L162F, V364A, D206Y, K600N, S109L, H195Y, H195Y/F683L, V212I, P336S/K26N, A25V/A396T/I679N, D693N, R518M, L320F, A403V, F683L, L760M, D368N, D427N/A576T, E398K, K676E, and E701K). The inhibitory effect implies that these hotspots do not allow SARS-CoV and SARS-CoV-2 to bind with them, hence resistant to COVID-19. However, the non-inhibitory effect indicates that these mutants have increased association of SARS-CoV and SARS-CoV-2, hence having higher chances of infection with COVID-19.

Nevertheless, a comparison of ClusPro with other tools was considered because its results were observed to have the highest similarity compared with experimental data. In the patient sample by ClusPro among 155 mutants the inhibited interaction for SARS-CoV was observed with 85 mutants (E35K, E37K, L73S, R115W, R204I, G205V, D269N, S280Y, M579T, T593I/P729S, S280Y/Q598H, T324S, W477R, R708Q, F762L, A99S, A311V, S409L, N601I, L120I/V658L, E189K, I358F, G395V, E398K, E489K, P590L, N578S/Y497C, D355N, E667K, S692F, N720S, L760M, R768L, A25V/A396T/I679N, K577N, D431G, V581I, S47C/P284S, H34N, I256M, R273K, N338D, D368N, R393G, A403V, E406K, P426S, I468T, R716H, P336S/K26N, Y613H, K131Q/F683L, E375D/K577N/R768W, F72C, P138S, V293I, M383I, D494G, I694M, W48L/N437H, L39M, A296T, G337E, V364A, P426L, V672A, R716C, P178S/E182D/D427N, S128I/R169I/S602Y, D269Y, W473L, I761T, T496A, K625T, R169I/H195Y/N394H, H195Y, Q305L, H195Y/F683L, L320F, K676E, L116F, E701K, P565L, V748F, M82T/F314L/K600N). However, no inhibition with SARS-CoV was identified for 70 mutants (P737H, D427N/A576T, G220C, R766K, Q325P, P737L, D213G, S44L, R357S, G561R, F28L, N194K/R306I/E479D, R219H, G211W, I618M, E232K, G399R, F683L, R219P, N599K, D615Y, L162F, V491L, R393I/E571G/R768W, D367V, R768W, W302G/F400L, Y202H, D597E, D713N, G764R, G272C, K419T, L628F, V670L, V488M, G352W, G405W, L450P, E22D, V59D, E182D, A264S, S317F, R644Q, E145K/E639K, R115Q, M462I, T334R, G147V, A412T, S109L, D206Y, K458T, R518M, K600N, D693N, A242T, L8F, T20I, V212I, N330H, D609N, R775I, P780S, D785N, G789R, T798P, T803I, and Q18K/G268C/W610L). Similarly, ClusPro inhibited interaction for SARS-CoV-2 was observed by 56 mutants (L39M, V59D, F72C, A99S, P138S, E182D, V293I, A296T, S317F, M383I, P426L, V581I, I618M, K625T, V672A, I694M, R716C, N720S, S47C/P284S, E145K/E639K, P178S/E182D/D427N, L116F, I761T, S44L, F28L, G272C, P565L, G399R, G352W, R219P, R219H, R204I, G205V, L628F, V488M, F762L, D213G, D427N/A576T, Q325P, P737L, M82T/F314L/K600N, G147V, D269Y, G337E, G405W, N338D, D269N, P737H, G764R, D609N, N599K, G561R, V748F, R766K, G220C, R357S). However, non-inhibitory interaction for SARS-CoV-2 was identified with 99 mutants (E22D, T20I, L760M, R768W, R768L, V212I, A242T, L162F, K419T, V491L, G395V, V670L, Y202H, L450P, I358F, S409L, A311V, M462I, S109L, D206Y, G211W, K458T, R518M, K600N, D693N, E398K, W477R, K577N, S692F, R115Q, E189K, A412T, E35K, L73S, S280Y, W473L, T593I/P729S, S280Y/Q598H, R169I/H195Y/N394H, R393I/E571G/R768W, E232K, T496A, F683L, D713N, N194K/R306I/E479D, A25V/A396T/I679N, A264S, T334R, D367V, D431G, D597E, K676E, W302G/F400L, L120I/V658L, P336S/K26N, H195Y, Q305L, R393G, E406K, D615Y, E667K, H195Y/F683L, L320F, E701K, L8F, H34N, E37K, R115W, I256M, R273K, T324S, N330H, D355N, V364A, D368N, A403V, P426S, I468T, E489K, D494G, M579T, P590L, N601I, Y613H, R644Q, R708Q, R716H, R775I, P780S, D785N, G789R, T798P, T803I, K131Q/F683L, W48L/N437H, N578S/Y497C, S128/R169I/S602Y, E375D/K577N/R768W, Q18K/G268C/W610L). SOAP-PP docking of SARS-CoV signifies the highest inhibitory effect by 80 mutants compared to ClusPro 85 mutants, while 14 compared to ClusPro 70 mutants have a non-inhibitory effect. Similarly, PatchDock docking compared to ClusPro indicates the inhibitory effect for SARS-CoV-2 by 51 out of 56 mutants, while 12/99 mutants have a non-inhibitory effect. In contrast, PatchDock docking signifies that no mutant has an inhibiting effect on SARS-CoV, while all 70 mutants have a non-inhibitory effect. Comparatively, SOAP-PP docked mutants show the highest inhibition correlation for SARS-CoV with experimentally validated mutants, while PatchDock displays the highest inhibition correlation with experimentally validated mutants for SARS-CoV-2. Also, InterEvDock2 demonstrated the highest cumulative 105 mutants out of 155 mutants, in which 14 mutants out of 56 have inhibitory, and the highest number of 91 mutants out of 99 have non-inhibitory effect with SARS-CoV-2.

FRODOCK2 exhibited the highest correlated 97 mutants, wherein 63 mutants out of 85 have inhibitory, and 34 mutants out of 70 have a non-inhibitory effect on interaction with SARS-CoV. In contrast, cumulative 89 mutants affect the interaction with SARS-CoV-2, wherein 33 mutants out of 56 have inhibitory, and 56 mutants out of 99 have a non-inhibitory effect. These results signify that the SOAP-PP has the highest reliability score of inhibition over other docking methods, followed by FRODOCK2 and InterEvDock2 because these methods show the highest cumulative score.

Nonetheless, the hotspot F72C, G205V, D269Y, E406K, E489K, M579T, R708Q, N720S, S128IR169I/S602Y mutant observed corroborated to validated results of ClusPro due to its highest inhibitory effect on interaction with SARS-CoV identified by five docking tools. Also, six docking tools have identified the highest inhibitory effect on the interaction of mutants, G205V, G352W, K625T, and I761T with SARS-CoV-2 signifies the dissociation of binding between them. The mutant G205V was observed to have the highest inhibitory effect in both SARS-CoV and SARS-CoV-2. The mutant T798P demonstrated the highest non-inhibition effect on binding with SARS-CoV experimental result correlation with predicted docking approaches by all seven tools simultaneously. Likewise, the mutants L8F have no inhibitory effect on binding with SARS-CoV-2 predicted by seven docking tools. These docking results are corroborated with the validated tool ClusPro mutants’ impact on inhibition and non-inhibition of interaction with SARS-CoV and SARS-CoV-2. Moreover, ZDOCK identified the mutant Q325P with the lowest binding affinity, which signifies that this mutant significantly inhibits its association with SARS-CoV. Although the lowest binding affinity of a single mutant, R768W with SARS-CoV-2 indicates the significant dissociation of their binding.

Conversely, the highest binding affinity of a single mutant, V748F, increases the binding/association with SARS-CoV, though the mutant S280Y increases the association with SARS-CoV-2. The ClusPro identified a hotspot multiple mutant M82T/F314L/K600N, which disrupts the binding/association with SARS-CoV, but the R357S mutant disrupts the association with SARS-CoV-2. However, a single mutant P737H and a single mutant E22Dimply the highest potential to increase the binding/association with SARS-CoV, and SARS-CoV-2, respectively. The HDOCK docking score exhibits that the M579T mutant strongly inhibits the association with SARS-CoV, while multiple mutants, K131Q/F683L, strongly inhibit the association with SARS-CoV-2. Nonetheless, D713N mutant strongly exhibits no inhibition on binding with SARS-CoV, as well as with SARS-CoV-2. PatchDock has identified all mutants, which increase the binding affinity with SARS-CoV, thereby signifying the mutants’ non-inhibiting effect with mutant P737L has the highest No-Inhibition effect. Also, the D597E mutant shows the highest binding affinity with SARS-CoV-2. However, a multiple mutant S47C/P284S shows the lowest binding affinity with SARS-CoV-2. InterEvDock2 exposed a mutant, G220C, and a single mutant, I761T, which have the highest inhibiting effect with SARS-CoV, and SARS-CoV-2, respectively. In contrast, a single mutant G561R has no inhibiting effect on binding with SARS-CoV, while mutant R768W has no inhibiting effect on binding with SARS-CoV, while mutant R768W has no inhibiting effect on binding with SARS-CoV-2. SOAP-PP signifies that the ACE2 mutant, G764R, and L628F, potentially inhibit the binding with SARS-CoV, and SARS-CoV-2, respectively. Conversely, the R219H mutant exhibits the highest inhibiting effect on SARS-CoV, whereas Q325P displays on SARS-CoV-2. Lastly, FRODOCK2 signifies that the ACE2 single mutant, G561R, potentially disrupts the association with SARS-CoV, while R219H inhibits the association with SARS-CoV-2. Nevertheless, mutants P737H and D367V have increased binding affinity with SARS-CoV, and SARS-CoV-2, which signifies their non-inhibiting effect.

Finally, the mutant complexes of patients’ samples were compared with experimental complexes to find the common mutants. None of the mutants found common between experimentally known mutants and in-patient mutants. However, some mutants present in the empirical study and patient data are common at amino acid impact on positions. Only a change of 1 amino acid is noticed rest of the mutant is similar. Hence, it needs further validation to identify the effect in experimental or patient studies. Experimental mutant E37A is identical to patient mutant E37K, except there is a change of one amino acid, “K,” which, might induce a difference in the binding affinity with SARS-CoV and SARS-CoV-2. In experimental studies, E37A showed a no-inhibitory effect with SARS-CoV, while in patient docking, E37K shows inhibition with SARS-CoV while neutral with SARS-CoV-2. An experimental mutant T324A displays a non-inhibitory effect with SARS-CoV in experimental studies, but docking of patient mutant T324S results indicates inhibitory and neutral binding effect with SARS-CoV and SARS-CoV-2, respectively. Likewise, the experimental mutant R219D and patient docked mutants R219P and R219H have a non-inhibitory effect with SARS-CoV, while an inhibitory effect was observed for SARS-CoV-2. A similar pattern was observed in the experimental mutant D355A, and patient docked mutant D355N indicates an inhibitory effect with SARS-CoV, while patient mutant D355N suggests a neutral effect on binding with SARS-CoV-2. Again, the same pattern was observed in an experimental mutant R393A and patient mutant R393G, which has an inhibitory impact with SARS-CoV, while a non-inhibitory effect was displayed for SARS-CoV-2. The inhibitory effect was exhibited with experimental mutant R357A and patient mutant R357S for SARS-CoV and SARS-CoV-2, respectively. However, patient mutant R357S shows a non-inhibitory effect for SARS-CoV. Interestingly, the inhibitory effect of experimental mutant M383A and patient mutant M383I have been demonstrated for SARS-CoV and SARS-CoV-2.

A comparison of cell lines’ docking ClusPro results with other tools has been performed (Supplementary Table 5). Docking analysis performed on the cell line data identified that 19 out of 25 mutants, N322I, A25V, L100V, V184A, S218N, Y252C, P253T, T276K, T334A, A413V, K416N, P426L, Q472P, P612L, W635L, Y649C, A386T/F314I, F603C/K619N, and L664I/D382Y, have an inhibited interaction with SARS-CoV. However, six mutants, S5F, A782V, F314L/Y510H, E457K, E668K, and E145K/E495K/I233S, revealed non-inhibitory interaction with SARS-CoV. Likewise, 11 mutants, W635L, L100V, S218N, Y252C, T276K, N322I, A413V, P426L, A386T/F314I, F603C/K619N, and E145K/E495K/I233S, have an inhibited interaction with SARS-CoV-2 based on ClusPro predicted values. On the other hand, 14 mutants, S5F, V184A, T334A, Y649C, A25V, P253T, K416N, E457K, Q472P, P612L, E668K, and F314L/Y510H, displayed a non-inhibitory interaction with SARS-CoV-2. For ACE2 mutants and SARS-CoV binding, SOAP-PP predicted the highest number of inhibitory interactions, 23 out of 25, compared to 19 mutants indicated by ClusPro. This corresponds with the experimental data, wherein the maximum number of inhibitory interactions were also demonstrated in SOAP-PP scoring, 27 out of 32 mutants. Interestingly, SOAP-PP revealed two non-inhibitory bindings, S5F and A782V, between SARS-CoV and ACE2 mutants within the cell lines instead of the six identified by ClusPro. However, PatchDock recognized the highest number of non-inhibitory interactions between SARS-CoV and ACE2 mutants, 24 out of 25, contrary to the six recognized by ClusPro. This relates to the experimental data, in which all 32 mutants were defined with a non-inhibitory role with SARS-CoV. Similarly, ZDOCK and HDOCK also identified 21 and 23 mutants, respectively, with a non-inhibitory effect on SARS-CoV binding among the cell lines. Weighing up the cell lines and experimental data for SARS-CoV and ACE2 mutants, SOAP-PP docked scores for inhibitory interactions and PatchDock scores for non-inhibitory interactions were significantly more reliable since more correlation was seen between the two sets of data than in the ClusPro scores. Collating the interactions of the mutants with SARS-CoV-2, PatchDock scores illustrated 21 out of 25 mutants with an inhibitory effect, compared to 11 identified by ClusPro. This correlates with the experimental data wherein PatchDock revealed 30 out of 32 mutants to show an inhibitory effect. Although, HDOCK summarized 21 mutants possessing a non-inhibitory impact with SARS-CoV-2 binding than the 14 mutants observed in ClusPro. This also correlates with the experimental data since the maximum number of non-inhibitory mutants were identified by HDOCK, as many as 22 out of 32. Examining the non-inhibitory binding effect of the wild-type ACE2 with both SARS-CoV and SARS-CoV-2, InterEvDock2 scores predicted 21 and 22 mutants displaying such an effect, respectively. This correlates with the 17 out of 32 mutants scanned by InterEvDock2 in experimental data with a neutral binding to SARS-CoV-2, the maximum number identified compared to other docking tools. Concerning the SARS-CoV-2 and ACE2 mutants data set, more association among the PatchDock within inhibitory and HDOCK within non-inhibitory scores of both the cell lines and experimental data sets were found, debasing reliability over the ClusPro scores. InterEvDock2 illustrated a higher correlation of the cell lines data among the neutral binding values with the experimental data.

Nonetheless, cell lines-based comparison was also performed to identify the cell lines’ hotspots (Supplementary Table 5). Two single mutants, S5F and A782V, were associated with a non-inhibitory binding interaction by 7 and 5 out of 7 docking tools towards SARS-CoV and by 7 and 4 out of 7 tools towards SARS-CoV-2, respectively (ClusPro, HDOCK, PatchDock, InterEvDock2, SOAP-PP, and FRODOCK2). Four mutants, L100V, P426L, A386T/F314I, and F603C/K619N, were certified as the hotspots of inhibitory binding towards both SARS-CoV and SARS-CV-2, based on the prediction values of ClusPro, SOAP-PP, and FRODOCK2 tools. Additionally, based on only ClusPro and SOAP-PP scoring, five mutants, S218N, Y252C, T276K, N322I, A413V, and W635L, showed similar inhibitory binding affinity towards SARS-CoV and SARS-CoV-2. However, the least binding affinity for SARS-CoV was attained by F603C/K619N and E145K/E495K/I233S, and for SARS-CoV-2 by Y649C, as revealed by at least two tools. Similarly, within three mutants, S218N, P253T, and E457K, non-inhibitory interaction for both SARS-CoV and SARS-CoV-2 was reported by three docking tools. S218N was identified as a hotspot mutant for enhanced SARS-CoV-2 binding, with the highest score predicted by PatchDock and HDOCK. A comparison between mutants identified between the cell lines and experimental data was performed to find any possible common mutants. However, the mutants between these two data sets was found no commonality.

The mutant sequences of ACE2 in patients and cell lines tend to associate or dissociate with the S-protein of SARS-CoV-2. The experimentally validated 33 sequences consist of 16 mutants and a wild-type that were found to have a binding affinity towards S-protein. In contrast, 17 mutants were shown to have the disrupted interaction with the S-protein. These 33 sequences were included as training and test datasets in the ratio of 80:20, respectively. It has been observed that autocorrelation-based descriptors performed the best when employed in various classifiers. Also, among the different classification models, RF and DT classifiers outperformed others in accuracy, sensitivity, specificity, and AUC. The best performing classification model was used to predict the binding outcome for the 156 patient mutants and 25 cell line mutants. All the detailed results of the analysis can be found in Supplementary Tables 1 and 2. We observed that the large number of mutants in both the samples were disrupting the bindings to SARS-CoV-2. Here, we have listed the top 10 scorer mutants from each sample that were found to disrupt the binding of ACE2 with SARS-CoV-2; patients: T593I, P729S, D355N, A403V, E406K, K600N, I358F, D368N, K577N, Q305L, and P565L; Cell Lines: E668K, N322I, P426L, Q472P, A413V, T334A, P612L, E145K, E495K, I233S, and A25V. We would like to emphasize the one major limitation of the developed classification models is the small amount of data available for building the models. We believe that further improvement can be possible in these models subjective the availability of the data shortly.

The present study analyzed GEPIA datasets and revealed ACE2 expression in different cancer and normal tissues. Survival analysis was performed for the ACE2 gene with significant impacts on overall survival identified in other cancer types. GEPIA uses the log-rank test for the evaluation of the hypothesis. The Cox proportional hazard ratio and 95% confidence interval score were included in the Fig. 4. All the analysis has been carried out using median cut-off as the threshold here, a well-accepted parameter for doing such studies^49^. Survival analysis depicts that higher expression of ACE2 is directly associated with the overall survival of the patients in KIRP, PCPG, LUSC, ACC, LIHC, SARC, STAD, PRAD, UCS, OV, COAD, UCEC, THYM, BLCA, MESO, CESC, KIRC, UVM, READ, CHOL, and HNSC cancers. In addition, a reverse scenario was observed for the cancers like LGG, DLBC, ESCA, GBM, PAAD, LUAD, SKCM, and TGCT, i.e., higher expression of ACE2 inversely correlated with the overall survival of the patients. We would like to mention that ACE2 expression in LIHC, LGG, OV, and KIRC cancers significantly associated with the overall survival of the patients. Our results suggest that ACE2 expression in cancers may serve a regulatory role in combating COVID-19. ACE2 was found to be downregulated in the cancers such as TGCT, THCA, and KICH, whereas upregulated was observed in the COAD, KIRP, PAAD, READ, STAD, and LUAD cancers^10^. However, the mechanism remains unclear, and future studies are required to elucidate the pathways involved in mutated ACE2 expression in cancers. The results of this study were obtained through in silico data analysis, and validation of the results via animal experiments and clinical trials is required.

## Conclusion

This study is used to identify which ACE2 mutations are cancer-associated and which are not associated with cancer. Further, these mutations were used to predict the impact on binding with SARS-CoV and SARS-CoV-2. The mutations that do not have any deleterious or severe impact and have a lower affinity with CoVs would likely be potential key hotspots to prevent COVID-19 infection.

## Supplementary Information

Supplementary Tables.

## Data Availability

The datasets generated and/or analyzed during the current study are available upon request from the corresponding author.

## References

[1] Lai CC, Shih TP, Ko WC, Tang HJ, Hsueh PR (2020). Severe acute respiratory syndrome coronavirus 2 (SARS-CoV-2) and coronavirus disease-2019 (COVID-19): The epidemic and the challenges. Int. J. Antimicrob. Agents.

[2] Sohrabi, C., Alsafi, Z., O’Neill, N., Khan, M., Kerwan, A. & Al-Jabir, A. C. World Health Organization declares global emergency: A review of the 2019 novel coronavirus (Covid-19).* Int. J. Surg.* **76** (2020). 10.1016/j.ijsu.2020.02.034 PMC7105032 32112977

[3] Raghav PK, Kalyanaraman K, Kumar D (2021). Human cell receptors: Potential drug targets to combat COVID-19. Amino Acids.

[4] https://covid19.who.int/

[5] Xie Y, Karki CB, Du D, Li H, Wang J, Sobitan A, Teng S, Tang Q, Li L (2020). Spike proteins of SARS-CoV and SARS-CoV-2 utilize different mechanisms to bind with human ACE2. Front. Mol. Biosci..

[6] Yi C, Sun X, Ye J, Ding L, Liu M, Yang Z, Lu X, Zhang Y, Ma L, Gu W, Qu A, Xu J, Shi Z, Ling Z, Sun B (2020). Key residues of the receptor binding motif in the spike protein of SARS-CoV-2 that interact with ACE2 and neutralizing antibodies. Cell. Mol. Immunol..

[7] Saponaro F, Rutigliano G, Sestito S, Bandini L, Storti B, Bizzarri R, Zucchi R (2020). ACE2 in the era of SARS-CoV-2: Controversies and novel perspectives. Front. Mol. Biosci..

[8] Pinto BG, Oliveira AE, Singh Y, Jimenez L, Gonçalves AN, Ogava RL, Creighton R, Peron JPS, Nakaya HI (2020). ACE2 expression is increased in the lungs of patients with comorbidities associated with severe COVID-19. J. Infect. Dis..

[9] Dai YJ, Hu F, Li H, Huang HY, Wang DW, Liang Y (2020). A profiling analysis on the receptor ACE2 expression reveals the potential risk of different type of cancers vulnerable to SARS-CoV-2 infection. Ann. Transl. Med..

[10] Gottschalk G, Knox K, Roy A (2021). ACE2: At the crossroad of COVID-19 and lung cancer. Gene Rep..

[11] Zhang Z, Li L, Li M, Wang X (2020). The SARS-CoV-2 host cell receptor ACE2 correlates positively with immunotherapy response and is a potential protective factor for cancer progression. Comput. Struct. Biotechnol. J..

[12] Bailey MH, Tokheim C, Porta-Pardo E, Sengupta S, Bertrand D, Weerasinghe A (2018). Comprehensive characterization of cancer driver genes and mutations. Cell.

[13] Hart SN, Polley EC, Yussuf A, Yadav S, Goldgar DE, Hu C, LaDuca H, Smith LP, Fujimoto J, Li S, Couch FJ, Dolinsky JS (2020). Mutation prevalence tables for hereditary cancer derived from multigene panel testing. Hum. Mutat..

[14] Torkamani A, Schork NJ (2008). Prediction of cancer driver mutations in protein kinases. Cancer Res..

[15] Harvey WT, Carabelli AM, Jackson B, Gupta RK, Thomson EC, Harrison EM (2021). SARS-CoV-2 variants, spike mutations and immune escape. Nat. Rev. Microbiol..

[16] Chen Y, Liu L, Wei Q, Zhu H, Jiang H, Tu X, Qin C, Chen Z (2008). Rhesus angiotensin converting enzyme 2 supports entry of severe acute respiratory syndrome coronavirus in Chinese macaques. Virology.

[17] Li W, Zhang C, Sui J, Kuhn JH, Moore MJ, Luo S (2005). Receptor and viral determinants of SARS-coronavirus adaptation to human ACE2. EMBO J..

[18] Rushworth CA, Guy JL, Turner AJ (2008). Residues affecting the chloride regulation and substrate selectivity of the angiotensin-converting enzymes (ACE and ACE2) identified by site-directed mutagenesis. FEBS J..

[19] Guy JL, Jackson RM, Jensen HA, Hooper NM, Turner AJ (2005). Identification of critical active-site residues in angiotensin-converting enzyme-2 (ACE2) by site-directed mutagenesis. FEBS J..

[20] Jia HP, Look DC, Tan P, Shi L, Hickey M, Gakhar L, Chappell MC, Wohlford-Lenane C, McCray PB (2009). Ectodomain shedding of angiotensin converting enzyme 2 in human airway epithelia. Am. J. Physiol. Lung Cell. Mol. Physiol..

[21] Koohi-Moghadam M, Wang H, Wang Y, Yang X, Li H, Wang J, Sun H (2019). Predicting disease-associated mutation of metal-binding sites in proteins using a deep learning approach. Nat. Mach. Intell..

[22] Gao HN, Lu HZ, Cao B, Du B, Shang H, Gan JH (2013). Clinical findings in 111 cases of influenza A (H7N9) virus infection. N. Engl. J. Med..

[23] Ferrer-Costa C, Orozco M, de la Cruz X (2002). Characterization of disease-associated single amino acid polymorphisms in terms of sequence and structure properties. J. Mol. Biol..

[24] Sitbon E, Pietrokovski S (2007). Occurrence of protein structure elements in conserved sequence regions. BMC Struct. Biol..

[25] Fischer A, Greenman C, Mustonen V (2011). Germline fitness-based scoring of cancer mutations. Genetics.

[26] Stitziel NO, Tseng YY, Pervouchine D, Goddeau D, Kasif S, Liang J (2003). Structural location of disease-associated single-nucleotide polymorphisms. J. Mol. Biol..

[27] Sunyaev S, Ramensky V, Bork P (2000). Towards a structural basis of human non-synonymous single nucleotide polymorphisms. Trends Genet..

[28] Todd AE, Orengo CA, Thornton JM (2002). Plasticity of enzyme active sites. Trends Biochem. Sci..

[29] Valdar WS (2002). Scoring residue conservation. Proteins Struct. Funct. Bioinform..

[30] Chakravarty D, Gao J, Phillips S, Kundra R, Zhang H, Wang J (2017). OncoKB: A precision oncology knowledge base. JCO Precis. Oncol..

[31] Chang MT, Bhattarai TS, Schram AM, Bielski CM, Donoghue MT, Jonsson P (2018). Accelerating discovery of functional mutant alleles in cancer. Cancer Discov..

[32] Gao J, Chang MT, Johnsen HC, Gao SP, Sylvester BE, Sumer SO, Zhang H, Solit DB, Taylor BS, Schultz N, Sander C (2017). 3D clusters of somatic mutations in cancer reveal numerous rare mutations as functional targets. Genome Med..

[33] Merid SK, Goranskaya D, Alexeyenko A (2014). Distinguishing between driver and passenger mutations in individual cancer genomes by network enrichment analysis. BMC Bioinform..

[34] Ng PC, Henikoff S (2003). SIFT: Predicting amino acid changes that affect protein function. Nucleic Acids Res..

[35] Vaser R, Adusumalli S, Leng SN, Sikic M, Ng PC (2016). SIFT missense predictions for genomes. Nat. Protoc..

[36] Adzhubei I, Jordan DM, Sunyaev SR (2013). Predicting functional effect of human missense mutations using PolyPhen-2. Curr. Protoc. Hum. Genet..

[37] Schwede T, Kopp J, Guex N, Peitsch MC (2003). SWISS-MODEL: An automated protein homology-modeling server. Nucleic Acids Res..

[38] Pierce BG, Wiehe K, Hwang H, Kim BH, Vreven T, Weng Z (2014). ZDOCK server: Interactive docking prediction of protein–protein complexes and symmetric multimers. Bioinformatics.

[39] Kozakov D, Hall DR, Xia B, Porter KA, Padhorny D, Yueh C, Beglov D, Vajda S (2017). The ClusPro web server for protein–protein docking. Nat. Protoc..

[40] Yan Y, Zhang D, Zhou P, Li B, Huang SY (2017). HDOCK: A web server for protein–protein and protein–DNA/RNA docking based on a hybrid strategy. Nucleic Acids Res..

[41] Mashiach E, Schneidman-Duhovny D, Peri A, Shavit Y, Nussinov R, Wolfson HJ (2010). An integrated suite of fast docking algorithms. Proteins Struct. Funct. Bioinform..

[42] Mashiach E, Schneidman-Duhovny D, Andrusier N, Nussinov R, Wolfson HJ (2008). FireDock: A web server for fast interaction refinement in molecular docking. Nucleic Acids Res..

[43] Xiao N, Cao DS, Zhu MF, Xu QS (2015). protr/ProtrWeb: R package and web server for generating various numerical representation schemes of protein sequences. Bioinformatics.

[44] Grun, B. & Leisch, F. FlexMix version 2: Finite mixtures with concomitant variables and varying and constant parameters (2008).

[45] Maleki F, Muthukrishnan N, Ovens K, Reinhold C, Forghani R (2020). Machine learning algorithm validation: From essentials to advanced applications and implications for regulatory certification and deployment. Neuroimaging Clin..

[46] Tang Z, Li C, Kang B, Gao G, Li C, Zhang Z (2017). GEPIA: A web server for cancer and normal gene expression profiling and interactive analyses. Nucleic Acids Res..

[47] Li B, Feng W, Luo O, Xu T, Cao Y, Wu H, Yu D, Ding Y (2017). Development and validation of a three-gene prognostic signature for patients with hepatocellular carcinoma. Sci. Rep..

[48] Wu J, Lou Y, Ma YM, Xu J, Shi T (2021). A novel risk-score model with eight MiRNA signatures for overall survival of patients with lung adenocarcinoma. Front. Genet..

[49] Mishra NK, Southekal S, Guda C (2019). Survival analysis of multi-omics data identifies potential prognostic markers of pancreatic ductal adenocarcinoma. Front. Genet..

