# Supplementary material for: Experimental and clinical data analysis for identification of COVID-19 resistant ACE2 mutations
Source: Sci Rep. 2023 Feb 9;13:2351. doi: 10.1038/s41598-022-20773-9 (PMC9910265; doi:10.1038/s41598-022-20773-9)
Supplement: Supplementary file 1 — Supplementary Tables. [file 41598_2022_20773_MOESM1_ESM.docx]

**Experimental and clinical data analysis for identification of COVID-19 resistance ACE2 mutations**

Pawan Kumar Raghav^1^*, Aditya Raghav^2^, Anjali Lathwal^3^, Archit Saxena^4^, Zoya Mann^2^**,** Manisha Sengar^5^, Raja Rajalingam^1^

^1^Immunogenetics and Transplantation Laboratory, Department of Surgery, University of California San Francisco, San Francisco, California, USA.

^2^BioExIn, Delhi, India

^3^Department of Computational Biology, Indraprastha Institute of Information Technology, New Delhi, India

^4^Amity Institute of Biotechnology, Amity University, Sector-125, Noida, Uttar Pradesh, India

^5^Department of Zoology, Deshbandhu College, University of Delhi, Delhi, India

***Correspondence:**

Dr. Pawan Kumar Raghav, Immunogenetics and Transplantation Laboratory, Department of Surgery, University of California San Francisco, San Francisco, California, USA; Email: PwnRghv@gmail.com, Pawan.Raghav@ucsf.edu; Ph: +1(415)-476-0795; Fax: +1(415)-476-0379

**Running Title:** Impact of ACE2 mutations in cancer and interaction with SARS-CoV-2

**Supplementary Tables Caption**

Supplementary Table 1. Machine learning prediction of patient samples.

Supplementary Table 2. Machine learning prediction of cell line samples.

Supplementary Table 3. Inference score (inhibition and no-inhibition) calculated by binding of experimental verified ACE2 mutant with SARS-CoV and SARS-CoV-2.

Supplementary Table 4. Inference score (inhibition and no-inhibition) calculated by binding of patient ACE2 mutant with SARS-CoV and SARS-CoV-2.

Supplementary Table 5. Inference score (inhibition and no-inhibition) calculated by binding of cell line ACE2 mutant with SARS-CoV and SARS-CoV-2.

**Supplementary Table 1.**

| **Sample_ID** | **Cancer_Type** | **Mutants** | **Function** | **Score** |
| --- | --- | --- | --- | --- |
| Pt15 | Melanoma | L8F | Abolished Binding with SARS-CoV-2 | 0.991226 |
| TCGA-D3-A2JP-06 | Cutaneous Melanoma | Q18K, G268C, W610L | Abolished Binding with SARS-CoV-2 | 0.984554 |
| ME009 | Cutaneous Melanoma | T20I | Abolished Binding with SARS-CoV-2 | 0.936849 |
| TCGA-ER-A19H-06 | Cutaneous Melanoma | E22D | Abolished Binding with SARS-CoV-2 | 0.988182 |
| 587376 | Colorectal Adenocarcinoma | S47C, P284S | Abolished Binding with SARS-CoV-2 | 0.991966 |
| TCGA-AP-A1DK-01 | Uterine Endometrioid Carcinoma | W48L, N437H | Abolished Binding with SARS-CoV-2 | 0.974973 |
| coadread_dfci_2016_3646 | Colorectal Adenocarcinoma | V59D | Abolished Binding with SARS-CoV-2 | 0.994359 |
| TCGA-EO-A3B0-01 | Uterine Endometrioid Carcinoma | F72C | Abolished Binding with SARS-CoV-2 | 0.934714 |
| YUZINO | Cutaneous Melanoma | S109L | Abolished Binding with SARS-CoV-2 | 0.985472 |
| coadread_dfci_2016_1794, TCGA-FF-A7CW-01 | Colorectal Adenocarcinoma, Diffuse Large B-Cell Lymphoma, NOS | R115Q | Abolished Binding with SARS-CoV-2 | 0.975123 |
| TCGA-ZF-A9RG-01 | Bladder Urothelial Carcinoma | L116F | Abolished Binding with SARS-CoV-2 | 0.98375 |
| TCGA-B5-A1MR-01 | Uterine Endometrioid Carcinoma | L120I, V658L | Abolished Binding with SARS-CoV-2 | 0.986798 |
| TCGA-B5-A11E-01 | Uterine Endometrioid Carcinoma | S128I, R169I, S602Y | Abolished Binding with SARS-CoV-2 | 0.929986 |
| TCGA-AX-A1CE-01, TCGA-D8-A1JG-01 | Uterine Endometrioid Carcinoma, Breast Invasive Ductal Carcinoma | L162F | Abolished Binding with SARS-CoV-2 | 0.962729 |
| TCGA-AG-A002-01 | Rectal Adenocarcinoma | R169I, H195Y, N394H | Abolished Binding with SARS-CoV-2 | 0.987164 |
| TCGA-NA-A5I1-01 | Uterine Carcinosarcoma/Uterine Malignant Mixed Mullerian Tumor | E182D | Abolished Binding with SARS-CoV-2 | 0.974798 |
| TCGA-AP-A056-01 | Uterine Endometrioid Carcinoma | N194K, R306I, E479D | Abolished Binding with SARS-CoV-2 | 0.97028 |
| TCGA-VQ-A8E7-01 | Tubular Stomach Adenocarcinoma | Y202H | Abolished Binding with SARS-CoV-2 | 0.992465 |
| TCGA-AP-A059-01 | Uterine Endometrioid Carcinoma | R204I | Abolished Binding with SARS-CoV-2 | 0.941506 |
| coadread_dfci_2016_1254 | Colorectal Adenocarcinoma | V212I | Abolished Binding with SARS-CoV-2 | 0.990377 |
| TCGA-BR-4292-01 | Stomach Adenocarcinoma | R219H | Abolished Binding with SARS-CoV-2 | 0.991068 |
| TCGA-44-2659-01 | Lung Adenocarcinoma | R219P | Abolished Binding with SARS-CoV-2 | 0.980017 |
| TCGA-D3-A2JB-06 | Cutaneous Melanoma | G272C | Abolished Binding with SARS-CoV-2 | 0.996261 |
| TCGA-D1-A103-01 | Uterine Endometrioid Carcinoma | R273K | Abolished Binding with SARS-CoV-2 | 0.968842 |
| TCGA-2J-AABP-01 | Pancreatic Adenocarcinoma | V293I | Abolished Binding with SARS-CoV-2 | 0.992788 |
| coadread_dfci_2016_116 | Colorectal Adenocarcinoma | W302G, F400L | Abolished Binding with SARS-CoV-2 | 0.999375 |
| H104362 | Hepatocellular Adenoma | Q305L | Abolished Binding with SARS-CoV-2 | 0.94587 |
| TCGA-69-7980-01 | Lung Adenocarcinoma | L320F | Abolished Binding with SARS-CoV-2 | 0.990631 |
| TCGA-AA-A01P-01 | Colon Adenocarcinoma | T324S | Abolished Binding with SARS-CoV-2 | 0.975799 |
| TCGA-CU-A0YR-01 | Bladder Urothelial Carcinoma | Q325P | Abolished Binding with SARS-CoV-2 | 0.961386 |
| TCGA-D7-6528-01 | Tubular Stomach Adenocarcinoma | N330H | Abolished Binding with SARS-CoV-2 | 0.927919 |
| MO_1288 | Breast Invasive Ductal Carcinoma | T334R | Abolished Binding with SARS-CoV-2 | 0.943646 |
| MEL-IPI_Pat74-Tumor-SM-4DK2Z, Pat74, TCGA-AA-3977-01 | Cutaneous Melanoma, Cutaneous Melanoma, Colon Adenocarcinoma | G337E | Abolished Binding with SARS-CoV-2 | 0.980061 |
| TCGA-VQ-A8P2-01 | Mucinous Stomach Adenocarcinoma | N338D | Abolished Binding with SARS-CoV-2 | 0.977047 |
| TCGA-06-1801-01 | Glioblastoma Multiforme | I358F | Abolished Binding with SARS-CoV-2 | 0.990221 |
| TCGA-AA-3947-01 | Mucinous Adenocarcinoma of the Colon and Rectum | V364A | Abolished Binding with SARS-CoV-2 | 0.973899 |
| TCGA-D1-A17Q-01 | Uterine Endometrioid Carcinoma | E375D, K577N, R768W | Abolished Binding with SARS-CoV-2 | 0.959294 |
| Au8 | Desmoplastic Melanoma | R393G | Abolished Binding with SARS-CoV-2 | 0.932193 |
| TCGA-EO-A22U-01 | Uterine Endometrioid Carcinoma | R393I, E571G, R768W | Abolished Binding with SARS-CoV-2 | 0.990244 |
| MO_1433 | Lung Adenocarcinoma | M462I | Abolished Binding with SARS-CoV-2 | 0.984195 |
| TCGA-92-7341-01 | Lung Squamous Cell Carcinoma | W477R | Abolished Binding with SARS-CoV-2 | 0.994822 |
| MEL-IPI_Pat62-Tumor-SM-4DK2N, Pat62 | Cutaneous Melanoma, Cutaneous Melanoma | V488M | Abolished Binding with SARS-CoV-2 | 0.935494 |
| TCGA-22-1016-01 | Lung Squamous Cell Carcinoma | V491L | Abolished Binding with SARS-CoV-2 | 0.984506 |
| TCGA-ZJ-AB0H-01 | Cervical Squamous Cell Carcinoma | T496A | Abolished Binding with SARS-CoV-2 | 0.98185 |
| TCGA-A5-A1OF-01 | Uterine Mixed Endometrial Carcinoma | R518M | Abolished Binding with SARS-CoV-2 | 0.986573 |
| 5-PT027-T1 | Skin Cancer, Non-Melanoma | P565L | Abolished Binding with SARS-CoV-2 | 0.99702 |
| TCGA-06-6389-01 | Glioblastoma Multiforme | V581I | Abolished Binding with SARS-CoV-2 | 0.958911 |
| Pat_41_Post | Melanoma | T593I, P729S | Abolished Binding with SARS-CoV-2 | 0.955143 |
| FR9547 | Lung Adenocarcinoma | N599K | Abolished Binding with SARS-CoV-2 | 0.997691 |
| DFCI-CLL139-Tumor | Chronic Lymphocytic Leukemia/Small Lymphocytic Lymphoma | N601I | Abolished Binding with SARS-CoV-2 | 0.995052 |
| TCGA-AU-6004-01 | Colon Adenocarcinoma | Y613H | Abolished Binding with SARS-CoV-2 | 0.995978 |
| TCGA-AA-A010-01 | Colon Adenocarcinoma | L628F | Abolished Binding with SARS-CoV-2 | 0.973477 |
| TCGA-BR-4201-01 | Stomach Adenocarcinoma | R644Q | Abolished Binding with SARS-CoV-2 | 0.988984 |
| TCGA-55-8506-01 | Lung Adenocarcinoma | V670L | Abolished Binding with SARS-CoV-2 | 0.985455 |
| MSKCC-0411_R | Bladder Urothelial Carcinoma | V672A | Abolished Binding with SARS-CoV-2 | 0.988464 |
| CRUK0027-R2 | Non-Small Cell Lung Cancer | K676E | Abolished Binding with SARS-CoV-2 | 0.953539 |
| TCGA-L5-A8NQ-01 | Esophageal Squamous Cell Carcinoma | S692F | Abolished Binding with SARS-CoV-2 | 0.956312 |
| coadread_dfci_2016_102, TCGA-RD-A8NB-01 | Colorectal Adenocarcinoma, Diffuse Type Stomach Adenocarcinoma | R708Q | Abolished Binding with SARS-CoV-2 | 0.976865 |
| coadread_dfci_2016_3064 | Colorectal Adenocarcinoma | R716H | Abolished Binding with SARS-CoV-2 | 0.902039 |
| CHC2128T | Hepatocellular Carcinoma | P737H | Abolished Binding with SARS-CoV-2 | 0.972928 |
| CSCC-31-T | Cutaneous Squamous Cell Carcinoma | P737L | Abolished Binding with SARS-CoV-2 | 0.971393 |
| NCH-CA-3 | Colorectal Adenocarcinoma | V748F | Abolished Binding with SARS-CoV-2 | 0.991371 |
| TCGA-19-1390-01 | Glioblastoma Multiforme | R766K | Abolished Binding with SARS-CoV-2 | 0.950272 |
| TCGA-BS-A0UJ-01 | Uterine Endometrioid Carcinoma | R775I | Abolished Binding with SARS-CoV-2 | 0.932951 |
| TCGA-55-8205-01 | Lung Adenocarcinoma | T798P | Abolished Binding with SARS-CoV-2 | 0.967652 |
| TCGA-VS-A958-01 | Cervical Squamous Cell Carcinoma | T803I | Abolished Binding with SARS-CoV-2 | 0.949053 |
| Pt1 | Melanoma | G789R | Abolished Binding with SARS-CoV-2 | 0.975632 |
| SC_9081-TM, YURED | Prostate Adenocarcinoma, Cutaneous Melanoma | D785N | Abolished Binding with SARS-CoV-2 | 0.985862 |
| TCGA-ER-A1A1-06 | Cutaneous Melanoma | P780S | Abolished Binding with SARS-CoV-2 | 0.987448 |
| 5-PT001-T1 | Skin Cancer, Non-Melanoma | G764R | Abolished Binding with SARS-CoV-2 | 0.976896 |
| TCGA-4A-A93Y-01 | Papillary Renal Cell Carcinoma | F762L | Abolished Binding with SARS-CoV-2 | 0.972786 |
| AMPAC_719 | Ampullary Carcinoma | I761T | Abolished Binding with SARS-CoV-2 | 0.956141 |
| TCGA-AJ-A3BH-01 | Uterine Endometrioid Carcinoma | L760M | Abolished Binding with SARS-CoV-2 | 0.977042 |
| OS-47-SJ | Osteosarcoma | N720S | Abolished Binding with SARS-CoV-2 | 0.970458 |
| DLBCL-RICOVER_1150 | Activated B-cell Type | D713N | Abolished Binding with SARS-CoV-2 | 0.991445 |
| 5-VS045-T1 | Skin Cancer, Non-Melanoma | E701K | Abolished Binding with SARS-CoV-2 | 0.986143 |
| TCGA-CR-6484-01 | Head and Neck Squamous Cell Carcinoma | I694M | Abolished Binding with SARS-CoV-2 | 0.985302 |
| TCGA-73-4658-01 | Lung Adenocarcinoma | D693N | Abolished Binding with SARS-CoV-2 | 0.989882 |
| TCGA-AG-3892-01, TCGA-F5-6814-01 | Rectal Adenocarcinoma, Rectal Adenocarcinoma | F683L | Abolished Binding with SARS-CoV-2 | 0.988775 |
| MO_1146 | Cutaneous Melanoma | E667K | Abolished Binding with SARS-CoV-2 | 0.964255 |
| TCGA-AA-A00N-01 | Mucinous Adenocarcinoma of the Colon and Rectum | K625T | Abolished Binding with SARS-CoV-2 | 0.980346 |
| TCGA-EK-A2RN-01 | Cervical Squamous Cell Carcinoma | I618M | Abolished Binding with SARS-CoV-2 | 0.956152 |
| TCGA-AA-3984-01 | Colon Adenocarcinoma | D609N | Abolished Binding with SARS-CoV-2 | 0.981786 |
| TCGA-A5-A2K5-01 | Uterine Endometrioid Carcinoma | K600N | Abolished Binding with SARS-CoV-2 | 0.981019 |
| TCGA-ND-A4WC-01 | Uterine Carcinosarcoma/Uterine Malignant Mixed Mullerian Tumor | D597E | Abolished Binding with SARS-CoV-2 | 0.983115 |
| TCGA-EO-A22R-01 | Uterine Endometrioid Carcinoma | N578S, Y497C | Abolished Binding with SARS-CoV-2 | 0.986893 |
| TCGA-A5-A0G2-01 | Uterine Serous Carcinoma/Uterine Papillary Serous Carcinoma | K577N | Abolished Binding with SARS-CoV-2 | 0.935375 |
| 5-NB008-T1 | Skin Cancer, Non-Melanoma | G561R | Abolished Binding with SARS-CoV-2 | 0.996792 |
| TCGA-Z2-A8RT-06 | Cutaneous Melanoma | E489K | Abolished Binding with SARS-CoV-2 | 0.934526 |
| coadread_dfci_2016_3498 | Colorectal Adenocarcinoma | I468T | Abolished Binding with SARS-CoV-2 | 0.975164 |
| TCGA-D7-6527-01 | Papillary Stomach Adenocarcinoma | K458T | Abolished Binding with SARS-CoV-2 | 0.913502 |
| TCGA-24-1564-01 | Serous Ovarian Cancer | L450P | Abolished Binding with SARS-CoV-2 | 0.990695 |
| 19424739 | Renal Non-Clear Cell Carcinoma | D431G | Abolished Binding with SARS-CoV-2 | 0.993077 |
| TCGA-FI-A2D5-01 | Uterine Endometrioid Carcinoma | D427N, A576T | Abolished Binding with SARS-CoV-2 | 0.990195 |
| LSD4744, LSD4744_T | Cutaneous Melanoma | P426S | Abolished Binding with SARS-CoV-2 | 0.993034 |
| TCGA-BS-A0UF-01 | Uterine Endometrioid Carcinoma | K419T | Abolished Binding with SARS-CoV-2 | 0.978181 |
| TCGA-BR-7707-01 | Stomach Adenocarcinoma | A412T | Abolished Binding with SARS-CoV-2 | 0.99253 |
| MEL-JWCI-WGS-12 | Cutaneous Melanoma | E406K | Abolished Binding with SARS-CoV-2 | 0.977935 |
| H072511 | Hepatocellular Adenoma | G405W | Abolished Binding with SARS-CoV-2 | 0.998385 |
| CRUK0001-R1, CRUK0001-R2, CRUK0001-R3 | Non-Small Cell Lung Cancer | A403V | Abolished Binding with SARS-CoV-2 | 0.974066 |
| YULAN | Cutaneous Melanoma | G399R | Abolished Binding with SARS-CoV-2 | 0.998998 |
| 585208 | Small Cell Lung Cancer | E398K | Abolished Binding with SARS-CoV-2 | 0.985693 |
| TCGA-56-7731-01 | Lung Squamous Cell Carcinoma | G395V | Abolished Binding with SARS-CoV-2 | 0.962336 |
| Pat_22_Post, Pat_22_Pre | Melanoma | D368N | Abolished Binding with SARS-CoV-2 | 0.982274 |
| WA48 | Prostate Adenocarcinoma | D367V | Abolished Binding with SARS-CoV-2 | 0.997077 |
| LUAD-LIP77 | Lung Adenocarcinoma | R357S | Abolished Binding with SARS-CoV-2 | 0.997425 |
| MEL-IPI_Pat11-Tumor-SM-4DK17, Pat11 | Melanoma of Unknown Primary, Cutaneous Melanoma | D355N | Abolished Binding with SARS-CoV-2 | 0.989712 |
| coadread_dfci_2016_341 | Colorectal Adenocarcinoma | G352W | Abolished Binding with SARS-CoV-2 | 0.999066 |
| TCGA-B5-A11H-01 | Uterine Endometrioid Carcinoma | P336S, K26N | Abolished Binding with SARS-CoV-2 | 0.980714 |
| TCGA-D3-A8GI-06 | Cutaneous Melanoma | A311V | Abolished Binding with SARS-CoV-2 | 0.96886 |
| TCGA-BA-A6DA-01 | Head and Neck Squamous Cell Carcinoma | A296T | Abolished Binding with SARS-CoV-2 | 0.996991 |
| 5-VS009-T2 | Skin Cancer, Non-Melanoma | D269N | Abolished Binding with SARS-CoV-2 | 0.976455 |
| TCGA-A5-A0G1-01 | Uterine Serous Carcinoma/Uterine Papillary Serous Carcinoma | D269Y | Abolished Binding with SARS-CoV-2 | 0.972665 |
| MO_1111 | Lung Adenocarcinoma | A264S | Abolished Binding with SARS-CoV-2 | 0.976742 |
| TCGA-95-7948-01, TCGA-HC-A76W-01 | Lung Adenocarcinoma, Prostate Adenocarcinoma | I256M | Abolished Binding with SARS-CoV-2 | 0.981222 |
| CLL-GCLL-0034-Tumor-SM-41JMX | Chronic Lymphocytic Leukemia/Small Lymphocytic Lymphoma | A242T | Abolished Binding with SARS-CoV-2 | 0.982475 |
| HN_62652 | Head and Neck Squamous Cell Carcinoma | E232K | Abolished Binding with SARS-CoV-2 | 0.978588 |
| coadread_dfci_2016_197 | Colorectal Adenocarcinoma | D213G | Abolished Binding with SARS-CoV-2 | 0.973129 |
| coadread_dfci_2016_3094 | Colorectal Adenocarcinoma | D206Y | Abolished Binding with SARS-CoV-2 | 0.986342 |
| PR4046, PR4046_T | Melanoma | G205V | Abolished Binding with SARS-CoV-2 | 0.922765 |
| TCGA-EE-A29N-06 | Cutaneous Melanoma | H195Y | Abolished Binding with SARS-CoV-2 | 0.985609 |
| TCGA-AP-A1E0-01 | Uterine Endometrioid Carcinoma | H195Y, F683L | Abolished Binding with SARS-CoV-2 | 0.985609 |
| Pt26 | Melanoma | E189K | Abolished Binding with SARS-CoV-2 | 0.927546 |
| TCGA-06-5416-01 | Glioblastoma Multiforme | P178S, E182D, D427N | Abolished Binding with SARS-CoV-2 | 0.969452 |
| 5-VS022-T1 | Skin Cancer, Non-Melanoma | E145K, E639K | Abolished Binding with SARS-CoV-2 | 0.990154 |
| TCGA-EE-A3AG-06 | Cutaneous Melanoma | P138S | Abolished Binding with SARS-CoV-2 | 0.957393 |
| TCGA-AX-A0J0-01 | Uterine Endometrioid Carcinoma | K131Q, F683L | Abolished Binding with SARS-CoV-2 | 0.988122 |
| TCGA-EY-A5W2-01 | Uterine Endometrioid Carcinoma | R115W | Abolished Binding with SARS-CoV-2 | 0.961968 |
| TCGA-62-A46R-01 | Lung Adenocarcinoma | A99S | Abolished Binding with SARS-CoV-2 | 0.990204 |
| TCGA-BS-A0UV-01 | Uterine Endometrioid Carcinoma | M82T, F314L, K600N | Abolished Binding with SARS-CoV-2 | 0.996085 |
| TCGA-UF-A719-01 | Head and Neck Squamous Cell Carcinoma | L73S | Abolished Binding with SARS-CoV-2 | 0.989828 |
| TCGA-AP-A0LM-01 | Uterine Endometrioid Carcinoma | L39M | Abolished Binding with SARS-CoV-2 | 0.993337 |
| TCGA-VR-AA7B-01 | Esophageal Squamous Cell Carcinoma | E37K | Abolished Binding with SARS-CoV-2 | 0.970061 |
| TCGA-B5-A3FA-01 | Uterine Endometrioid Carcinoma | E35K | Abolished Binding with SARS-CoV-2 | 0.978442 |
| TCGA-99-7458-01 | Lung Adenocarcinoma | H34N | Abolished Binding with SARS-CoV-2 | 0.984788 |
| coadread_dfci_2016_593 | Colorectal Adenocarcinoma | F28L | Abolished Binding with SARS-CoV-2 | 0.985589 |
| TCGA-DU-6392-01 | Astrocytoma | A25V, A396T, I679N | Abolished Binding with SARS-CoV-2 | 0.971063 |
| TCGA-EE-A2MR-06 | Cutaneous Melanoma | S44L | No Impact on Binding with SARS-CoV-2 | 0.780976 |
| MO_1072 | Penile Squamous Cell Carcinoma | G220C | No Impact on Binding with SARS-CoV-2 | 0.423312 |
| TCGA-FR-A729-06 | Cutaneous Melanoma | S317F | No Impact on Binding with SARS-CoV-2 | 0.856072 |
| TCGA-EE-A20F-06 | Cutaneous Melanoma | M383I | No Impact on Binding with SARS-CoV-2 | 0.845266 |
| TCGA-GN-A26C-01 | Cutaneous Melanoma | S409L | No Impact on Binding with SARS-CoV-2 | 0.87405 |
| CSCC-27-T | Cutaneous Squamous Cell Carcinoma | P426L | No Impact on Binding with SARS-CoV-2 | 0.835433 |
| TCGA-QK-A8Z8-01 | Head and Neck Squamous Cell Carcinoma | W473L | No Impact on Binding with SARS-CoV-2 | 0.899294 |
| TCGA-F1-6874-01 | Intestinal Type Stomach Adenocarcinoma | P590L | No Impact on Binding with SARS-CoV-2 | 0.817709 |
| TCGA-HZ-7922-01 | Pancreatic Adenocarcinoma | R716C | No Impact on Binding with SARS-CoV-2 | 0.697421 |
| LUAD_E00522 | Lung Adenocarcinoma | R768L | No Impact on Binding with SARS-CoV-2 | 0.872146 |
| TCGA-E6-A1LX-01 | Uterine Endometrioid Carcinoma | R768W | No Impact on Binding with SARS-CoV-2 | 0.883992 |
| SJERG016_D_WES | Acute Lymphoid Leukemia | D615Y | No Impact on Binding with SARS-CoV-2 | 0.801286 |
| PCNSL_4 | Diffuse Large B-Cell Lymphoma, NOS | M579T | No Impact on Binding with SARS-CoV-2 | 0.870354 |
| TCGA-AA-A022-01 | Colon Adenocarcinoma | D494G | No Impact on Binding with SARS-CoV-2 | 0.674958 |
| TCGA-AX-A05Z-01 | Uterine Endometrioid Carcinoma | S280Y | No Impact on Binding with SARS-CoV-2 | 0.747612 |
| TCGA-EE-A183-06 | Cutaneous Melanoma | S280Y, Q598H | No Impact on Binding with SARS-CoV-2 | 0.747612 |
| TCGA-44-7670-01 | Lung Adenocarcinoma | G211W | No Impact on Binding with SARS-CoV-2 | 0.061784 |
| TCGA-39-5035-01 | Lung Squamous Cell Carcinoma | G147V | No Impact on Binding with SARS-CoV-2 | 0.821235 |

**Supplementary Table 2.**

| **S.No.** | **Sample_ID** | **Cancer_Type** | **Mutants** | **Function** | **Score** |
| --- | --- | --- | --- | --- | --- |
| 1 | JSC1_HAEMATOPOIETIC_AND_LYMPHOID_TISSUE | Mixed Cancer Types | S5F | Abolished Binding with SARS-CoV-2 | 0.985613 |
| 2 | JHOS2_OVARY | Mixed Cancer Types | V184A | Abolished Binding with SARS-CoV-2 | 0.981135 |
| 3 | HEC59_ENDOMETRIUM | Mixed Cancer Types | S218N | Abolished Binding with SARS-CoV-2 | 0.984853 |
| 4 | SUDHL10_HAEMATOPOIETIC_AND_LYMPHOID_TISSUE | Mixed Cancer Types | T276K | Abolished Binding with SARS-CoV-2 | 0.994237 |
| 5 | MCC26_SKIN | Mixed Cancer Types | N322I | Abolished Binding with SARS-CoV-2 | 0.987779 |
| 6 | CAL54_KIDNEY | Mixed Cancer Types | T334A | Abolished Binding with SARS-CoV-2 | 0.988042 |
| 7 | LS411N_LARGE_INTESTINE | Mixed Cancer Types | Q472P | Abolished Binding with SARS-CoV-2 | 0.98643 |
| 8 | HCC_2998 | Colorectal Adenocarcinoma | F603C, K619N | Abolished Binding with SARS-CoV-2 | 0.965011 |
| 9 | HCC2998_LARGE_INTESTINE | Mixed Cancer Types | F603C, K619N | Abolished Binding with SARS-CoV-2 | 0.965011 |
| 10 | CORL32_LUNG | Mixed Cancer Types | W635L | Abolished Binding with SARS-CoV-2 | 0.910476 |
| 11 | HCT_15, HCT15_LARGE_INTESTINE | Colorectal Adenocarcinoma, Mixed Cancer Types | Y649C | Abolished Binding with SARS-CoV-2 | 0.995293 |
| 12 | JHUEM7_ENDOMETRIUM | Mixed Cancer Types | L664I, D382Y | Abolished Binding with SARS-CoV-2 | 0.98598 |
| 13 | MESSA_SOFT_TISSUE | Mixed Cancer Types | A782V | Abolished Binding with SARS-CoV-2 | 0.981695 |
| 14 | PECAPJ15_UPPER_AERODIGESTIVE_TRACT | Mixed Cancer Types | E668K | Abolished Binding with SARS-CoV-2 | 0.975889 |
| 15 | GMEL_SKIN | Mixed Cancer Types | E457K | Abolished Binding with SARS-CoV-2 | 0.990775 |
| 16 | EN_ENDOMETRIUM | Mixed Cancer Types | K416N | Abolished Binding with SARS-CoV-2 | 0.993628 |
| 17 | LS123_LARGE_INTESTINE | Mixed Cancer Types | A413V | Abolished Binding with SARS-CoV-2 | 0.927719 |
| 18 | HEC251_ENDOMETRIUM | Mixed Cancer Types | F314L, Y510H | Abolished Binding with SARS-CoV-2 | 0.951065 |
| 19 | NCIH513_PLEURA | Mixed Cancer Types | P253T | Abolished Binding with SARS-CoV-2 | 0.967574 |
| 20 | MCC13_SKIN | Mixed Cancer Types | E145K, E495K, I233S | Abolished Binding with SARS-CoV-2 | 0.990154 |
| 21 | OSRC2_KIDNEY | Mixed Cancer Types | L100V | Abolished Binding with SARS-CoV-2 | 0.974474 |
| 22 | ISHIKAWAHERAKLIO02ER_ENDOMETRIUM | Mixed Cancer Types | A25V | Abolished Binding with SARS-CoV-2 | 0.971063 |
| 23 | A172_CENTRAL_NERVOUS_SYSTEM | Mixed Cancer Types | Y252C | No Impact on Binding with SARS-CoV-2 | 0.870041 |
| 24 | LU165_LUNG | Mixed Cancer Types | P426L | No Impact on Binding with SARS-CoV-2 | 0.835433 |
| 25 | TMK1_STOMACH | Mixed Cancer Types | P612L | No Impact on Binding with SARS-CoV-2 | 0.851625 |

**Supplementary Table 3.**

| **Complexes** | **Experimentally Verified** | **Docking Verified** | | | | | | | | | | | | | |  |  |
| --- | --- | --- | --- | --- | --- | --- | --- | --- | --- | --- | --- | --- | --- | --- | --- | --- | --- |
|  |  | **ZDOCK** | | **CLUSPRO** | | **HDOCK** | | **PATCHDOCK** | | **INTEREVDOCK** | | **SOAP PP** | | **FRODOCK2** | | **INFERENCE SARS-CoV** | **INFERENCE SARS-CoV-2** |
| **wild** | **Binds with SARS-CoV** | **Binds with SARS-CoV** | Binds with SARS-CoV-2 | **Binds with SARS-CoV** | Binds with SARS-CoV-2 | **Binds with SARS-CoV** | Binds with SARS-CoV-2 | **Binds with SARS-CoV** | Binds with SARS-CoV-2 | **Binds with SARS-CoV** | Binds with SARS-CoV-2 | **Binds with SARS-CoV** | Binds with SARS-CoV-2 | **Binds with SARS-CoV** | Binds with SARS-CoV-2 | **Binds with SARS-CoV** |  |
| K31D | Inhibition | Inhibition | Inhibition | Inhibition | No Inhibition | Inhibition | No Inhibition | No Inhibition | Inhibition | **No Inhibition/Highest Affinity** | Neutral | Inhibition | Inhibition | No Inhibition | Inhibition | **C. Inhibition = 4/7**  **No inhibition = 3/7** | **Inhibition = 4/7**  **No inhibition = 3/7** |
| E37A | No Inhibition | No Inhibition | No Inhibition | No Inhibition | No Inhibition | Inhibition | No Inhibition | No Inhibition | Inhibition | Inhibition | No Inhibition/Highest Affinity | Inhibition | Inhibition | No Inhibition | Inhibition | **Inhibition = 3/7**  **C. No inhibition = 4/7** | **Inhibition = 3/7**  **No inhibition =4/7** |
| D38A | No Inhibition | No Inhibition | No Inhibition | No Inhibition | No Inhibition | No Inhibition | No Inhibition | No Inhibition | Inhibition | **No Inhibition/Highest Affinity** | No Inhibition | Inhibition | Inhibition | No Inhibition | Inhibition | **Inhibition = 1/7**  **No inhibition = 6/7** | **Inhibition = 3/7**  **No inhibition = 4/7** |
| Y41A | Inhibition | Inhibition | Inhibition | **Inhibition/Lowest affinity** | Inhibition | **Inhibition/Lowest affinity** | No Inhibition | No Inhibition | **No Inhibition/Highest Affinity** | Inhibition | No Inhibition/Highest Affinity | No Inhibition | Inhibition | **No Inhibition/Highest Affinity** | Inhibition | **Inhibition = 4/7**  **No inhibition = 3/7** | **Inhibition = 4/7**  **No inhibition = 3/7** |
| K68D | Inhibition | No Inhibition | No Inhibition | **Inhibition/Lowest affinity** | Inhibition | No Inhibition | Inhibition | No Inhibition | Inhibition | **No Inhibition/Highest Affinity** | Neutral | Inhibition | Inhibition | Inhibition | No Inhibition | **Inhibition = 3/7**  **No inhibition = 4/7** | **Inhibition = 4/7**  **No inhibition = 3/7** |
| E110P | No Inhibition | No Inhibition | No Inhibition | No Inhibition | No Inhibition | No Inhibition | No Inhibition | No Inhibition | Inhibition | **No Inhibition/Highest Affinity** | Neutral | Inhibition | No Inhibition | Inhibition | Inhibition | **Inhibition = 2/7**  **No inhibition = 5/7** | **Inhibition = 2/7**  **No inhibition =5//7** |
| E160R | No Inhibition | No Inhibition | No Inhibition | No Inhibition | No Inhibition | Inhibition | Inhibition | No Inhibition | Inhibition | Inhibition | Neutral | Inhibition | Inhibition | Inhibition | Inhibition | **Inhibition = 4/7**  **No inhibition = 3/7** | **Inhibition = 4/7**  **No inhibition = 3/7** |
| R192D | No Inhibition | No Inhibition | Inhibition | Inhibition | No Inhibition | No Inhibition | No Inhibition | No Inhibition | Inhibition | **No Inhibition/Highest Affinity** | Inhibition | Inhibition | No Inhibition/Highest Affinity | Inhibition | No Inhibition | **Inhibition = 3/7**  **No inhibition = 4/7** | **Inhibition = 3/7**  **No inhibition = 4/7** |
| R219D | No Inhibition | **Inhibition/Lowest affinity** | No Inhibition | No Inhibition | Inhibition | **No Inhibition/Highest Affinity** | No Inhibition | No Inhibition | Inhibition | Inhibition | Inhibition | **No Inhibition/Highest Affinity** | Inhibition | Inhibition | **Inhibition/Lowest affinity** | **Inhibition = 3/7**  **No inhibition = 4/7** | **Inhibition = 5/7**  **No inhibition = 2/7** |
| H239Q | No Inhibition | No Inhibition | No Inhibition | Inhibition | Neutral | No Inhibition | No Inhibition | No Inhibition | Inhibition | **No Inhibition/Highest Affinity** | Neutral | Inhibition | Inhibition | Inhibition | No Inhibition | **Inhibition = 3/7**  **No inhibition = 4/7** | **Inhibition = 2/7**  **No inhibition = 5/7** |
| K309D | No Inhibition | Inhibition | **No Inhibition/Highest Affinity** | No Inhibition | No Inhibition | No Inhibition | No Inhibition | No Inhibition | Inhibition | **No Inhibition/Highest Affinity** | No Inhibition | Inhibition | Inhibition | Inhibition | No Inhibition | **Inhibition = 3/7**  **No inhibition = 4/7** | **Inhibition = 2/7**  **No inhibition = 5/7** |
| E312A | No Inhibition | No Inhibition | Inhibition | No Inhibition | No Inhibition | Inhibition | No Inhibition | No Inhibition | Inhibition | Inhibition | No Inhibition | No Inhibition | Inhibition | No Inhibition | Inhibition | **Inhibition = 2/7**  **No inhibition = 5/7** | **Inhibition = 4/7**  **No inhibition = 3/7** |
| T324A | No Inhibition | Inhibition | Inhibition | **Inhibition/Lowest affinity** | Neutral | No Inhibition | No Inhibition | No Inhibition | Inhibition | Inhibition | Neutral | Inhibition | Inhibition | Inhibition | Inhibition | **Inhibition = 5/7**  **No inhibition = 2/7** | **Inhibition = 4/7**  **No inhibition = 3/7** |
| D350A | No Inhibition | No Inhibition | No Inhibition | Inhibition | Inhibition | No Inhibition | Inhibition | No Inhibition | Inhibition | **No Inhibition/Highest Affinity** | Neutral | Inhibition | Inhibition | Inhibition | No Inhibition | **Inhibition = 3/7**  **No inhibition = 4/7** | **Inhibition = 4/7**  **No inhibition = 3/7** |
| K353H | Inhibition | No Inhibition | No Inhibition | No Inhibition | Inhibition | No Inhibition | No Inhibition | No Inhibition | Inhibition | **No Inhibition/Highest Affinity** | Neutral | Inhibition | Inhibition | No Inhibition | **No Inhibition/Highest Affinity** | **Inhibition = 1/7**  **No inhibition = 6/7** | **Inhibition = 3/7**  **No inhibition = 4/7** |
| K353A | Inhibition | No Inhibition | No Inhibition | No Inhibition | Inhibition | No Inhibition | No Inhibition | No Inhibition | Inhibition | Inhibition | No Inhibition/Highest Affinity | Inhibition | Inhibition | Inhibition | Inhibition | **Inhibition = 3/7**  **No inhibition = 4/7** | **Inhibition = 4/7**  **No inhibition = 3/7** |
| K353D | Inhibition | No Inhibition | No Inhibition | Inhibition | Inhibition | No Inhibition | No Inhibition | No Inhibition | Inhibition | **No Inhibition/Highest Affinity** | Neutral | Inhibition | No Inhibition | Inhibition | Inhibition | **Inhibition = 3/7**  **No inhibition = 4/7** | **Inhibition = 3/7**  **No inhibition = 4/7** |
| D355A | Inhibition | No Inhibition | No Inhibition | No Inhibition | Inhibition | No Inhibition | No Inhibition | No Inhibition | No Inhibition | Inhibition | Neutral | No Inhibition | **Inhibition/Lowest affinity** | Inhibition | Inhibition | **Inhibition = 2/7**  **No inhibition = 5/7** | **Inhibition = 3/7**  **No inhibition = 4/7** |
| R357A | Inhibition | No Inhibition | No Inhibition | No Inhibition | No Inhibition | No Inhibition | No Inhibition | No Inhibition | Inhibition | Inhibition | No Inhibition/Highest Affinity | Inhibition | Inhibition | No Inhibition | Inhibition | **Inhibition = 2/7**  **No inhibition = 5/7** | **Inhibition = 3/7**  **No inhibition = 4/7** |
| L359K | No Inhibition | No Inhibition | No Inhibition | No Inhibition | Inhibition/Lowest affinity | No Inhibition | Inhibition | No Inhibition | Inhibition | **Inhibition/Lowest affinity** | Neutral | Inhibition | Inhibition | Inhibition | Inhibition | **Inhibition = 2/7**  **No inhibition = 5/7** | **Inhibition = 5/7**  **No inhibition = 2/7** |
| L359A | No Inhibition | No Inhibition | Inhibition | No Inhibition | No Inhibition/Highest Affinity | No Inhibition | Inhibition | No Inhibition | Inhibition | Inhibition | Neutral | Inhibition | Inhibition | No Inhibition | No Inhibition | **Inhibition = 2/7**  **No inhibition = 5/7** | **Inhibition = 4/7**  **No inhibition = 3/7** |
| M383A | Inhibition | No Inhibition | Inhibition | Inhibition | No Inhibition | No Inhibition | No Inhibition | No Inhibition | Inhibition | **No Inhibition/Highest Affinity** | No Inhibition | Inhibition | No Inhibition | No Inhibition | Inhibition | **Inhibition = 2/7**  **No inhibition = 5/7** | **Inhibition = 3/7**  **No inhibition = 4/7** |
| P389A | Inhibition | No Inhibition | No Inhibition | **Inhibition/Lowest affinity** | Inhibition | No Inhibition | No Inhibition | No Inhibition | Inhibition | **No Inhibition/Highest Affinity** | Neutral | Inhibition | Inhibition | Inhibition | No Inhibition | **Inhibition = 3/7**  **No inhibition = 4/7** | **Inhibition = 3/7**  **No inhibition = 4/7** |
| R393A | Inhibition | No Inhibition | Inhibition | Inhibition | No Inhibition | No Inhibition | No Inhibition/Highest Affinity | No Inhibition | Inhibition | Inhibition | No Inhibition | No Inhibition | Inhibition | No Inhibition | Inhibition | **Inhibition = 2/7**  **No inhibition = 5/7** | **Inhibition = 4/7**  **No inhibition = 3/7** |
| R559S | Inhibition | Inhibition | Inhibition | Inhibition | Neutral | No Inhibition | No Inhibition | No Inhibition | Inhibition | Inhibition | Neutral | Inhibition | Inhibition | Inhibition | Inhibition | **Inhibition = 5/7**  **No inhibition = 2/7** | **Inhibition = 4/7**  **No inhibition = 3/7** |
| F603T | No Inhibition | Inhibition  0 | Inhibition | Inhibition | No Inhibition | No Inhibition | Inhibition | No Inhibition | Inhibition/Lowest affinity | Inhibition | Inhibition | Inhibition | Inhibition | Inhibition | Inhibition | **Inhibition = 5/7**  **No inhibition = 2/7** | **Inhibition = 6/7**  **No inhibition = 1/7** |
| Q24K, A25A, K26E | Inhibition | No Inhibition  0 | No Inhibition | No Inhibition | Inhibition | Inhibition | No Inhibition | No Inhibition | Inhibition | Inhibition | Neutral | Inhibition | Inhibition | Inhibition | Inhibition | **Inhibition = 4/7**  **No inhibition = 3/7** | **Inhibition = 1/7**  **No inhibition = 6/7** |
| M82N, Y83F, P84S | Inhibition | No Inhibition  0 | No Inhibition | **No Inhibition/Highest Affinity** | Inhibition | No Inhibition | Inhibition | No Inhibition | Inhibition | **No Inhibition/Highest Affinity** | Inhibition | Inhibition | Inhibition | Inhibition | Inhibition | **Inhibition = 2/7**  **No inhibition = 5/7** | **Inhibition = 6/7**  **No inhibition = 1/7** |
| P135S, D136M | No Inhibition | No Inhibition | **Inhibition/Lowest affinity** | Inhibition | Neutral | No Inhibition | Inhibition/Lowest affinity | No Inhibition | Inhibition | **No Inhibition/Highest Affinity** | Neutral | **Inhibition/Lowest affinity** | Inhibition | No Inhibition | No Inhibition | **Inhibition = 2/7**  **No inhibition = 5/7** | **Inhibition = 4/7**  **No inhibition = 3/7** |
| N338D, V339D, Q340R | No Inhibition | Inhibition | No Inhibition | Inhibition | No Inhibition | No Inhibition | Inhibition | No Inhibition | Inhibition | Inhibition | Inhibition/Lowest affinity | Inhibition | Inhibition | **Inhibition/Lowest affinity** | Inhibition | **Inhibition = 5/7**  **No inhibition = 2/7** | **Inhibition = 5/7**  **No inhibition = 2/7** |
| S425P, P426S, D427N | Inhibition | No Inhibition | No Inhibition | No Inhibition | Inhibition | No Inhibition | No Inhibition | No Inhibition | Inhibition | Inhibition | Neutral | Inhibition | No Inhibition | No Inhibition | No Inhibition | **Inhibition = 2/7**  **No inhibition = 5/7** | **Inhibition = 2/7**  **No inhibition = 5/7** |
| K465Q, G466D, E467K | No Inhibition | **No Inhibition/Highest Affinity** | No Inhibition | No Inhibition | No Inhibition | No Inhibition | Inhibition | **No Inhibition/Highest Affinity** | Inhibition | Inhibition | No Inhibition | Inhibition | No Inhibition | Inhibition | Inhibition | **Inhibition = 3/7**  **No inhibition = 4/7** | **Inhibition = 3/7**  **No inhibition = 4/7** |
| **Common BA between SARS-CoV and SARS-CoV-2** | NA | **Inhibition: 05**  **No Inhibition: 17**  **Total common between SARS-CoV& SARS-CoV-2: 22** | | **Inhibition: 05**  **No Inhibition: 09**  **Total common between SARS-CoV& SARS-CoV-2: 13** | | **Inhibition: 01**  **No Inhibition: 17**  **Total common between SARS-CoV& SARS-CoV-2: 18** | | **Inhibition: 0**  **No Inhibition: 2**  **Total common between SARS-CoV& SARS-CoV-2: 2** | | **Inhibition: 3**  **No Inhibition: 12**  **Total common between SARS-CoV& SARS-CoV-2: 15** | | **Inhibition: 21**  **No Inhibition: 0**  **Total common between SARS-CoV& SARS-CoV-2: 20** | | **Inhibition: 14**  **No Inhibition: 4**  **Total common between SARS-CoV& SARS-CoV-2: 18** | |  |  |
| **CorrelatedScore**  **SARS-CoV& CoV-2** | Inhibition: 15/15  No inhibition: 17/17 | Inhibition: 3/15  No inhibition: 12/17  Tot: 15/32 | Inhibition: 5/15  No inhibition: 11/17  Tot: 16/32 | Inhibition: 8/15  No inhibition: 10/17  Tot: 18/32 | Inhibition: 10/15  No inhibition: 14/17**(H)**  Tot: 24/32 | Inhibition: 3/15  No inhibition: 14/17  Tot: 17/32 | Inhibition: 2/15  No inhibition: 9/17  Tot: 11/32 | Inhibition: 0/15  No inhibition: 17/17**(H)**  Tot: 17/32 | Inhibition: 13/15**(H)**  No inhibition: 0/17  Tot: 13/32 | Inhibition: 8/15  No inhibition: 7/17  Tot: 15/32 | Inhibition: 1/15  No inhibition: 13/17  Tot: 14/32 | Inhibition: 12/15**(H)**  No inhibition: 2/17  Tot: 14/32 | Inhibition: 12/15  No inhibition: 3/17  Tot: 15/32 | Inhibition: 8/15  No inhibition: 5/17  Tot: 13/32 | Inhibition: 11/15  No inhibition: 6/17  Tot: 17/32 |  |  |
|  | **EXP.** | **Cov** | **Cov-2** | **Cov** | **Cov-2** | **Cov** | **Cov-2** | **Cov** | **Cov-2** | **Cov** | **Cov-2** | **Cov** | **Cov-2** | **Cov** | **Cov-2** |  |  |
| **Complexes** | **Experimetally** | **ZDOCK** | | **CLUSPRO** | | **HDOCK** | | **PATCHDOCK** | | **INTEREVDOCK** | | **SOAP PP** | | **FRODOCK2** | |  |  |

**Supplementary Table 4.**

| **Complexes** | **Validated tool** | | **Docking Verified** | | | | | | | | | | | |  |  |
| --- | --- | --- | --- | --- | --- | --- | --- | --- | --- | --- | --- | --- | --- | --- | --- | --- |
|  | **(ClusPro)** | | **ZDOCK** | | **HDOCK** | | **PATCHDOCK** | | **INTEREVDOCK** | | **SOAP PP** | | **FRODOCK2** | | **INFERENCE** | **INFERENCE** |
| **Wild** | **Binds with SARS-CoV** | **Binds with SARS-CoV-2** | **Binds with SARS-CoV** | **Binds with SARS-CoV-2** | **Binds with SARS-CoV** | **Binds with SARS-CoV-2** | **Binds with SARS-CoV** | **Binds with SARS-CoV-2** | **Binds with SARS-CoV** | **Binds with SARS-CoV-2** | **Binds with SARS-CoV** | **Binds with SARS-CoV-2** | **Binds with SARS-CoV** | **Binds with SARS-CoV-2** | **Binds with SARS-CoV** | **Binds with SARS-CoV-2** |
| L8F | Neutral | Neutral | Inhibition | No Inhibition | Neutral | Neutral | Neutral | Neutral | Neutral | Neutral | Neutral | Neutral | Neutral | Neutral | Inhibition = 1/7  No Inhibition =6/7 | Inhibition = 0/7  No Inhibition = 7/7 |
| T20I | Neutral | No Inhibition | Inhibition | Inhibition | Neutral | Neutral | Neutral | Neutral | Neutral | Neutral | Neutral | Neutral | Neutral | Neutral | Inhibition = 1/7  No Inhibition = 6/7 | Inhibition = 1/7  No Inhibition = 6/7 |
| E22D | No Inhibition | No Inhibition | No Inhibition | Inhibition | Inhibition | Inhibition | No Inhibition | Inhibition | Inhibition | Neutral | Inhibition | No Inhibition | Inhibition | Inhibition | Inhibition = 4/7  No Inhibition = 3/7 | Inhibition = 4/7  No Inhibition = 3/7 |
| F28L | No Inhibition | Inhibition | No Inhibition | No Inhibition | No Inhibition | Inhibition | No Inhibition | Inhibition | No Inhibition | No Inhibition | Inhibition | Inhibition | Inhibition | Inhibition | Inhibition = 2/7  No Inhibition = 5/7 | Inhibition = 5/7  No Inhibition = 2/7 |
| H34N | Inhibition | Neutral | No Inhibition | Inhibition | Inhibition | No Inhibition | No Inhibition | Inhibition | Inhibition | No Inhibition | Inhibition | No Inhibition | No Inhibition | Inhibition | Inhibition = 4/7  No Inhibition = 3/7 | Inhibition = 3/7  No Inhibition = 4/7 |
| E35K | Inhibition | No Inhibition | No Inhibition | Inhibition | No Inhibition | No Inhibition | No Inhibition | Inhibition | Inhibition | Neutral | No Inhibition | Inhibition | Inhibition | No Inhibition | Inhibition = 3/7  No Inhibition = 4/7 | Inhibition = 3/7  No Inhibition = 4/7 |
| E37K | Inhibition | Neutral | No Inhibition | Inhibition | No Inhibition | No Inhibition | No Inhibition | Inhibition | Inhibition | No Inhibition | Inhibition | No Inhibition | Inhibition | No Inhibition | Inhibition = 4/7  No Inhibition = 3/7 | Inhibition = 2/7  No Inhibition = 5/7 |
| L39M | Inhibition | Inhibition | No Inhibition | No Inhibition | Inhibition | Inhibition | No Inhibition | Inhibition | No Inhibition | No Inhibition | Inhibition | Inhibition | Inhibition | No Inhibition | Inhibition = 4/7  No Inhibition = 3/7 | Inhibition = 4/7  No Inhibition = 3/7 |
| S44L | No Inhibition | Inhibition | No Inhibition | No Inhibition | No Inhibition | Inhibition | No Inhibition | Inhibition | No Inhibition | Neutral | Inhibition | Inhibition | Inhibition | Inhibition | Inhibition = 2/7  No Inhibition = 5/7 | Inhibition = 5/7  No Inhibition = 2/7 |
| V59D | No Inhibition | Inhibition | Inhibition | No Inhibition | Inhibition | Inhibition | No Inhibition | Inhibition | Inhibition | Neutral | Inhibition | Inhibition | Inhibition | Inhibition | Inhibition = 5/7  No Inhibition = 2/7 | Inhibition = 5/7  No Inhibition = 2/7 |
| F72C | Inhibition | Inhibition | Inhibition | Inhibition | Inhibition | No Inhibition | No Inhibition | No Inhibition | No Inhibition | No Inhibition | Inhibition | Inhibition | Inhibition | Inhibition | Inhibition = 5/7  No Inhibition = 2/7 | Inhibition = 4/7  No Inhibition = 3/7 |
| L73S | Inhibition | No Inhibition | No Inhibition | Inhibition | Inhibition | No Inhibition | No Inhibition | Inhibition | No Inhibition | Neutral | Inhibition | Inhibition | Inhibition | Inhibition | Inhibition = 4/7  No Inhibition = 3/7 | Inhibition = 4/7  No Inhibition = 3/7 |
| A99S | Inhibition | Inhibition | No Inhibition | No Inhibition | Inhibition | No Inhibition | No Inhibition | Inhibition | No Inhibition | Neutral | Inhibition | Inhibition | Inhibition | No Inhibition | Inhibition = 4/7  No Inhibition = 3/7 | Inhibition = 3/7  No Inhibition = 4/7 |
| S109L | No Inhibition | No Inhibition | No Inhibition | No Inhibition | No Inhibition | Inhibition | No Inhibition | Inhibition | Inhibition | Inhibition | Inhibition | Inhibition | No Inhibition | No Inhibition | Inhibition = 2/7  No Inhibition = 5/7 | Inhibition = 4/7  No Inhibition = 3/7 |
| R115Q | No Inhibition | No Inhibition | No Inhibition | No Inhibition | No Inhibition | Inhibition | No Inhibition | Inhibition | No Inhibition | Neutral | Inhibition | Inhibition | Inhibition | Inhibition | Inhibition = 2/7  No Inhibition = 5/7 | Inhibition = 4/7  No Inhibition = 3/7 |
| R115W | Inhibition | Neutral | No Inhibition | Inhibition | No Inhibition | Inhibition | No Inhibition | Inhibition | No Inhibition | Inhibition | Inhibition | Inhibition | No Inhibition | Inhibition | Inhibition = 2/7  No Inhibition = 5/7 | Inhibition = 6/7  No Inhibition = 1/7 |
| L116F | Inhibition | Inhibition | No Inhibition | No Inhibition | No Inhibition | No Inhibition | No Inhibition | Inhibition | No Inhibition | Inhibition | Inhibition | Inhibition | Inhibition | Inhibition | Inhibition = 3/7  No Inhibition = 4/7 | Inhibition = 5/7  No Inhibition = 2/7 |
| P138S | Inhibition | Inhibition | Inhibition | Inhibition | No Inhibition | Inhibition | No Inhibition | Neutral | No Inhibition | Neutral | Inhibition | Inhibition | Inhibition | No Inhibition | Inhibition = 4/7  No Inhibition = 3/7 | Inhibition = 4/7  No Inhibition = 3/7 |
| G147V | No Inhibition | Inhibition | No Inhibition | No Inhibition | No Inhibition | No Inhibition | No Inhibition | Inhibition | Inhibition | Neutral | Inhibition | Inhibition | Inhibition | Inhibition | Inhibition = 3/7  No Inhibition = 4/7 | Inhibition = 4/7  No Inhibition = 3/7 |
| L162F | No Inhibition | No Inhibition | No Inhibition | No Inhibition | No Inhibition | No Inhibition | No Inhibition | Inhibition | No Inhibition | Neutral | Inhibition | Inhibition | No Inhibition | No Inhibition | Inhibition = 1/7  No Inhibition = 6/7 | Inhibition = 2/7  No Inhibition = 5/7 |
| E182D | No Inhibition | Inhibition | No Inhibition | Inhibition | No Inhibition | No Inhibition | No Inhibition | Inhibition | No Inhibition | Neutral | Inhibition | Inhibition | Inhibition | Inhibition | Inhibition = 2/7  No Inhibition = 5/7 | Inhibition = 5/7  No Inhibition = 2/7 |
| E189K | Inhibition | No Inhibition | No Inhibition | No Inhibition | No Inhibition | No Inhibition | No Inhibition | Inhibition | Inhibition | Neutral | Inhibition | No Inhibition | Inhibition | No Inhibition | Inhibition = 4/7  No Inhibition = 3/7 | Inhibition = 1/7  No Inhibition = 6/7 |
| H195Y | Inhibition | No Inhibition | No Inhibition | No Inhibition | No Inhibition | No Inhibition | No Inhibition | Inhibition | Inhibition | Neutral | Inhibition | Inhibition | No Inhibition | No Inhibition | Inhibition = 3/7  No Inhibition = 4/7 | Inhibition = 2/7  No Inhibition = 5/7 |
| Y202H | No Inhibition | No Inhibition | No Inhibition | No Inhibition | Inhibition | No Inhibition | No Inhibition | Inhibition | Inhibition | Neutral | Inhibition | Inhibition | Inhibition | No Inhibition | Inhibition = 4/7  No Inhibition = 3/7 | Inhibition = 2/7  No Inhibition = 5/7 |
| R204I | Inhibition | Inhibition | Inhibition | No Inhibition | No Inhibition | No Inhibition | No Inhibition | Inhibition | Inhibition | Inhibition | No Inhibition | Inhibition | Inhibition | Inhibition | Inhibition = 4/7  No Inhibition = 3/7 | Inhibition = 5/7  No Inhibition = 2/7 |
| G205V | Inhibition | Inhibition | Inhibition | No Inhibition | Inhibition | Inhibition | No Inhibition | Inhibition | Inhibition | Inhibition | Inhibition | Inhibition | No Inhibition | Inhibition | Inhibition = 5/7  No Inhibition = 2/7 | Inhibition = 6/7  No Inhibition = 1/7 |
| D206Y | No Inhibition | No Inhibition | No Inhibition | No Inhibition | No Inhibition | Inhibition | No Inhibition | Inhibition | No Inhibition | Inhibition | Inhibition | Inhibition | No Inhibition | No Inhibition | Inhibition = 1/7  No Inhibition = 6/7 | Inhibition = 4/7  No Inhibition = 3/7 |
| G211W | No Inhibition | No Inhibition | No Inhibition | Inhibition | No Inhibition | Inhibition | No Inhibition | Inhibition | No Inhibition | Inhibition | Inhibition | Inhibition | No Inhibition | Inhibition | Inhibition = 1/7  No Inhibition = 6/7 | Inhibition = 6/7  No Inhibition = 1/7 |
| V212I | Neutral | No Inhibition | No Inhibition | No Inhibition | Inhibition | Inhibition | No Inhibition | Inhibition | No Inhibition | Neutral | Inhibition | No Inhibition | No Inhibition | No Inhibition | Inhibition = 2/7  No Inhibition = 5/7 | Inhibition = 2/7  No Inhibition = 5/7 |
| D213G | No Inhibition | Inhibition | No Inhibition | No Inhibition | No Inhibition | No Inhibition | No Inhibition | Inhibition | Inhibition | No Inhibition | Inhibition | Inhibition | Inhibition | Inhibition | Inhibition = 3/7  No Inhibition = 4/7 | Inhibition = 4/7  No Inhibition = 3/7 |
| R219P | No Inhibition | Inhibition | Inhibition | No Inhibition | No Inhibition | No Inhibition | No Inhibition | Inhibition | Inhibition | Inhibition | No Inhibition | Inhibition | Inhibition | Inhibition | Inhibition = 3/7  No Inhibition = 4/7 | Inhibition = 5/7  No Inhibition = 2/7 |
| R219H | No Inhibition | Inhibition | Inhibition | No Inhibition | Inhibition | No Inhibition | No Inhibition | Inhibition | Inhibition | Inhibition | No Inhibition | Inhibition | No Inhibition | Inhibition | Inhibition = 3/7  No Inhibition = 4/7 | Inhibition = 5/7  No Inhibition = 2/7 |
| G220C | No Inhibition | Inhibition | No Inhibition | No Inhibition | No Inhibition | No Inhibition | No Inhibition | Inhibition | Inhibition | Neutral | Inhibition | No Inhibition | Inhibition | Inhibition | Inhibition = 3/7  No Inhibition = 4/7 | Inhibition = 3/7  No Inhibition = 4/7 |
| E232K | No Inhibition | No Inhibition | No Inhibition | No Inhibition | Inhibition | No Inhibition | No Inhibition | Inhibition | Inhibition | Neutral | Inhibition | Inhibition | Inhibition | No Inhibition | Inhibition = 4/7  No Inhibition = 3/7 | Inhibition = 2/7  No Inhibition = 5/7 |
| A242T | No Inhibition | No Inhibition | No Inhibition | No Inhibition | Inhibition | No Inhibition | No Inhibition | Inhibition | No Inhibition | Neutral | Inhibition | Inhibition | Inhibition | Inhibition | Inhibition = 3/7  No Inhibition = 4/7 | Inhibition = 3/7  No Inhibition = 4/7 |
| I256M | Inhibition | Neutral | No Inhibition | Inhibition | No Inhibition | No Inhibition | No Inhibition | Inhibition | Neutral | Neutral | Inhibition | No Inhibition | Inhibition | No Inhibition | Inhibition = 3/7  No Inhibition = 4/7 | Inhibition = 2/7  No Inhibition = 5/7 |
| A264S | No Inhibition | No Inhibition | Inhibition | Inhibition | No Inhibition | No Inhibition | No Inhibition | Inhibition | Inhibition | Neutral | Inhibition | Inhibition | Neutral | Inhibition | Inhibition = 3/7  No Inhibition = 4/7 | Inhibition = 4/7  No Inhibition = 3/7 |
| D269N | Inhibition | Inhibition | Inhibition | Inhibition | No Inhibition | Inhibition | No Inhibition | Inhibition | No Inhibition | No Inhibition | Inhibition | Inhibition | Inhibition | No Inhibition | Inhibition = 4/7  No Inhibition = 3/7 | Inhibition = 5/7  No Inhibition = 2/7 |
| D269Y | Inhibition | Inhibition | No Inhibition | Inhibition | Inhibition | No Inhibition | No Inhibition | Inhibition | Inhibition | Neutral | Inhibition | Inhibition | Inhibition | No Inhibition | Inhibition = 5/7  No Inhibition = 2/7 | Inhibition = 4/7  No Inhibition = 3/7 |
| G272C | No Inhibition | Inhibition | No Inhibition | Inhibition | No Inhibition | No Inhibition | No Inhibition | Inhibition | No Inhibition | No Inhibition | Inhibition | No Inhibition | No Inhibition | Inhibition | Inhibition = 1/7  No Inhibition = 6/7 | Inhibition = 4/7  No Inhibition = 3/7 |
| R273K | Inhibition | Neutral | No Inhibition | Inhibition | No Inhibition | No Inhibition | No Inhibition | Inhibition | Inhibition | Neutral | Inhibition | Inhibition | Inhibition | No Inhibition | Inhibition = 4/7  No Inhibition = 3/7 | Inhibition = 3/7  No Inhibition = 4/7 |
| S280Y | Inhibition | No Inhibition | No Inhibition | No Inhibition | No Inhibition | No Inhibition | No Inhibition | Inhibition | No Inhibition | Neutral | Inhibition | Inhibition | Inhibition | Inhibition | Inhibition = 3/7  No Inhibition = 4/7 | Inhibition = 3/7  No Inhibition = 4/7 |
| V293I | Inhibition | Inhibition | No Inhibition | Inhibition | No Inhibition | Inhibition | No Inhibition | Inhibition | No Inhibition | Neutral | Inhibition | No Inhibition | Inhibition | Inhibition | Inhibition = 3/7  No Inhibition = 4/7 | Inhibition = 5/7  No Inhibition = 2/7 |
| A296T | Inhibition | Inhibition | No Inhibition | No Inhibition | No Inhibition | No Inhibition | No Inhibition | Inhibition | No Inhibition | Neutral | Inhibition | Inhibition | Inhibition | Inhibition | Inhibition = 3/7  No Inhibition = 4/7 | Inhibition = 4/7  No Inhibition = 3/7 |
| Q305L | Inhibition | No Inhibition | No Inhibition | Inhibition | No Inhibition | No Inhibition | No Inhibition | Inhibition | Inhibition | No Inhibition | Inhibition | Inhibition | Inhibition | No Inhibition | Inhibition = 4/7  No Inhibition = 3/7 | Inhibition = 3/7  No Inhibition = 4/7 |
| A311V | Inhibition | No Inhibition | No Inhibition | No Inhibition | No Inhibition | No Inhibition | No Inhibition | Inhibition | No Inhibition | Neutral | Inhibition | Inhibition | Inhibition | No Inhibition | Inhibition = 3/7  No Inhibition = 4/7 | Inhibition = 2/7  No Inhibition = 5/7 |
| S317F | No Inhibition | Inhibition | Inhibition | Inhibition | No Inhibition | No Inhibition | No Inhibition | Inhibition | No Inhibition | Inhibition | Inhibition | Inhibition | Inhibition | No Inhibition | Inhibition = 3/7  No Inhibition = 4/7 | Inhibition = 5/7  No Inhibition = 2/7 |
| L320F | Inhibition | No Inhibition | No Inhibition | No Inhibition | No Inhibition | No Inhibition | No Inhibition | Inhibition | No Inhibition | Neutral | Inhibition | Inhibition | No Inhibition | No Inhibition | Inhibition = 2/7  No Inhibition = 5/7 | Inhibition = 2/7  No Inhibition = 5/7 |
| T324S | Inhibition | Neutral | No Inhibition | No Inhibition | No Inhibition | No Inhibition | No Inhibition | Inhibition | No Inhibition | Neutral | Inhibition | Inhibition | Inhibition | No Inhibition | Inhibition = 3/7  No Inhibition = 4/7 | Inhibition = 2/7  No Inhibition = 5/7 |
| Q325P | No Inhibition | Inhibition | Inhibition | No Inhibition | Inhibition | No Inhibition | No Inhibition | Inhibition | Inhibition | No Inhibition | Inhibition | No Inhibition | Inhibition | Inhibition | Inhibition = 5/7  No Inhibition = 2/7 | Inhibition = 3/7  No Inhibition = 4/7 |
| N330H | Neutral | Neutral | No Inhibition | Inhibition | No Inhibition | Inhibition | No Inhibition | Inhibition | Inhibition | Neutral | Inhibition | Inhibition | Inhibition | Inhibition | Inhibition = 3/7  No Inhibition = 4/7 | Inhibition = 5/7  No Inhibition = 2/7 |
| T334R | No Inhibition | No Inhibition | No Inhibition | No Inhibition | No Inhibition | No Inhibition | No Inhibition | Inhibition | Neutral | Inhibition | Inhibition | Inhibition | Inhibition | No Inhibition | Inhibition = 2/7  No Inhibition = 5/7 | Inhibition = 3/7  No Inhibition = 4/7 |
| G337E | Inhibition | Inhibition | No Inhibition | Inhibition | No Inhibition | No Inhibition | No Inhibition | Inhibition | No Inhibition | Inhibition | Inhibition | Inhibition | Inhibition | No Inhibition | Inhibition = 3/7  No Inhibition = 4/7 | Inhibition = 5/7  No Inhibition = 2/7 |
| N338D | Inhibition | Inhibition | No Inhibition | Inhibition | Inhibition | No Inhibition | No Inhibition | Inhibition | No Inhibition | Neutral | Inhibition | Inhibition | Inhibition | No Inhibition | Inhibition = 4/7  No Inhibition = 3/7 | Inhibition = 4/7  No Inhibition = 3/7 |
| G352W | No Inhibition | Inhibition | No Inhibition | No Inhibition | No Inhibition | Inhibition | No Inhibition | Inhibition | No Inhibition | Inhibition | Inhibition | Inhibition | Inhibition | Inhibition | Inhibition = 2/7  No Inhibition = 5/7 | Inhibition = 6/7  No Inhibition = 1/7 |
| D355N | Inhibition | Neutral | Inhibition | Inhibition | No Inhibition | No Inhibition | No Inhibition | Inhibition | Neutral | Neutral | Inhibition | Inhibition | Inhibition | Inhibition | Inhibition = 4/7  No Inhibition = 3/7 | Inhibition = 4/7  No Inhibition = 3/7 |
| R357S | No Inhibition | Inhibition | No Inhibition | No Inhibition | Inhibition | Inhibition | No Inhibition | Inhibition | Inhibition | Neutral | Inhibition | Inhibition | Inhibition | Inhibition | Inhibition = 4/7  No Inhibition = 3/7 | Inhibition = 5/7  No Inhibition = 2/7 |
| I358F | Inhibition | No Inhibition | No Inhibition | No Inhibition | Inhibition | No Inhibition | No Inhibition | Inhibition | No Inhibition | Neutral | Inhibition | Inhibition | Inhibition | Inhibition | Inhibition = 4/7  No Inhibition = 3/7 | Inhibition = 3/7  No Inhibition = 4/7 |
| V364A | Inhibition | Neutral | No Inhibition | No Inhibition | No Inhibition | No Inhibition | No Inhibition | Inhibition | Inhibition | Neutral | Inhibition | Inhibition | No Inhibition | No Inhibition | Inhibition = 3/7  No Inhibition = 4/7 | Inhibition = 2/7  No Inhibition = 5/7 |
| D367V | No Inhibition | No Inhibition | No Inhibition | No Inhibition | No Inhibition | No Inhibition | No Inhibition | Inhibition | No Inhibition | Neutral | Inhibition | Inhibition | Inhibition | No Inhibition | Inhibition = 2/7  No Inhibition = 5/7 | Inhibition = 2/7  No Inhibition = 5/7 |
| D368N | Inhibition | Neutral | No Inhibition | Inhibition | Inhibition | No Inhibition | No Inhibition | Inhibition | No Inhibition | Neutral | Inhibition | Inhibition | No Inhibition | No Inhibition | Inhibition = 3/7  No Inhibition = 4/7 | Inhibition = 3/7  No Inhibition = 4/7 |
| M383I | Inhibition | Inhibition | Inhibition | Inhibition | No Inhibition | No Inhibition | No Inhibition | Inhibition | Neutral | No Inhibition | Inhibition | Inhibition | Inhibition | Inhibition | Inhibition = 4/7  No Inhibition = 3/7 | Inhibition = 5/7  No Inhibition = 2/7 |
| R393G | Inhibition | No Inhibition | No Inhibition | Inhibition | No Inhibition | Inhibition | No Inhibition | Inhibition | Inhibition | No Inhibition | Inhibition | Inhibition | Inhibition | Inhibition | Inhibition = 4/7  No Inhibition = 3/7 | Inhibition = 5/7  No Inhibition = 2/7 |
| G395V | Inhibition | No Inhibition | No Inhibition | No Inhibition | No Inhibition | Inhibition | No Inhibition | Inhibition | No Inhibition | Neutral | Inhibition | Inhibition | Inhibition | Inhibition | Inhibition = 3/7  No Inhibition = 4/7 | Inhibition = 4/7  No Inhibition = 3/7 |
| E398K | Inhibition | No Inhibition | No Inhibition | No Inhibition | No Inhibition | No Inhibition | No Inhibition | Inhibition | Inhibition | Inhibition | Inhibition | No Inhibition | No Inhibition | No Inhibition | Inhibition = 3/7  No Inhibition = 4/7 | Inhibition = 2/7  No Inhibition = 5/7 |
| G399R | No Inhibition | Inhibition | No Inhibition | No Inhibition | No Inhibition | No Inhibition | No Inhibition | Inhibition | No Inhibition | Neutral | Inhibition | Inhibition | No Inhibition | Inhibition | Inhibition = 1/7  No Inhibition = 6/7 | Inhibition = 4/7  No Inhibition = 3/7 |
| A403V | Inhibition | Neutral | No Inhibition | Inhibition | No Inhibition | No Inhibition | No Inhibition | Inhibition | No Inhibition | Neutral | Inhibition | Inhibition | No Inhibition | No Inhibition | Inhibition = 2/7  No Inhibition = 5/7 | Inhibition = 3/7  No Inhibition = 4/7 |
| G405W | No Inhibition | Inhibition | No Inhibition | No Inhibition | No Inhibition | No Inhibition | No Inhibition | Inhibition | No Inhibition | Neutral | Inhibition | Inhibition | Inhibition | No Inhibition | Inhibition = 2/7  No Inhibition = 5/7 | Inhibition = 3/7  No Inhibition = 4/7 |
| E406K | Inhibition | No Inhibition | No Inhibition | Inhibition | Inhibition | No Inhibition | No Inhibition | Inhibition | Inhibition | Neutral | Inhibition | Inhibition | Inhibition | Inhibition | Inhibition = 5/7  No Inhibition = 2/7 | Inhibition = 4/7  No Inhibition = 3/7 |
| S409L | Inhibition | No Inhibition | No Inhibition | No Inhibition | Inhibition | No Inhibition | No Inhibition | No Inhibition | No Inhibition | Neutral | Inhibition | Inhibition | Inhibition | Inhibition | Inhibition = 4/7  No Inhibition = 3/7 | Inhibition = 2/7  No Inhibition = 5/7 |
| A412T | No Inhibition | No Inhibition | No Inhibition | No Inhibition | No Inhibition | No Inhibition | No Inhibition | Inhibition | No Inhibition | Neutral | Inhibition | Inhibition | Inhibition | Inhibition | Inhibition = 2/7  No Inhibition = 5/7 | Inhibition = 3/7  No Inhibition = 4/7 |
| K419T | No Inhibition | No Inhibition | No Inhibition | No Inhibition | Inhibition | No Inhibition | No Inhibition | Inhibition | Inhibition | Inhibition | Inhibition | No Inhibition | No Inhibition | Inhibition | Inhibition = 3/7  No Inhibition = 4/7 | Inhibition = 3/7  No Inhibition = 4/7 |
| P426L | Inhibition | Inhibition | No Inhibition | No Inhibition | No Inhibition | Inhibition | No Inhibition | Inhibition | No Inhibition | Neutral | Inhibition | Inhibition | Inhibition | Inhibition | Inhibition = 3/7  No Inhibition = 4/7 | Inhibition = 5/7  No Inhibition = 2/7 |
| P426S | Inhibition | Neutral | No Inhibition | No Inhibition | No Inhibition | No Inhibition | No Inhibition | Inhibition | Inhibition | Neutral | Inhibition | Inhibition | Inhibition | No Inhibition | Inhibition = 4/7  No Inhibition = 3/7 | Inhibition = 2/7  No Inhibition = 5/7 |
| D431G | Inhibition | No Inhibition | No Inhibition | No Inhibition | No Inhibition | No Inhibition | No Inhibition | No Inhibition | Inhibition | Inhibition | Inhibition | Inhibition | Inhibition | Inhibition | Inhibition = 4/7  No Inhibition = 3/7 | Inhibition = 3/7  No Inhibition = 4/7 |
| L450P | No Inhibition | No Inhibition | No Inhibition | No Inhibition | No Inhibition | No Inhibition | No Inhibition | Inhibition | Inhibition | Neutral | Inhibition | Inhibition | Inhibition | Inhibition | Inhibition = 3/7  No Inhibition = 4/7 | Inhibition = 3/7  No Inhibition = 4/7 |
| K458T | No Inhibition | No Inhibition | No Inhibition | No Inhibition | No Inhibition | No Inhibition | No Inhibition | Inhibition | Inhibition | Neutral | Inhibition | Inhibition | Inhibition | No Inhibition | Inhibition = 3/7  No Inhibition = 4/7 | Inhibition = 2/7  No Inhibition = 5/7 |
| M462I | No Inhibition | No Inhibition | No Inhibition | No Inhibition | Inhibition | No Inhibition | No Inhibition | Inhibition | No Inhibition | Neutral | Inhibition | Inhibition | Inhibition | Inhibition | Inhibition = 3/7  No Inhibition = 4/7 | Inhibition = 3/7  No Inhibition = 4/7 |
| I468T | Inhibition | Neutral | No Inhibition | No Inhibition | No Inhibition | Inhibition | No Inhibition | Inhibition | Inhibition | Neutral | Inhibition | Inhibition | Inhibition | No Inhibition | Inhibition = 4/7  No Inhibition = 3/7 | Inhibition = 3/7  No Inhibition = 4/7 |
| W473L | Inhibition | No Inhibition | No Inhibition | No Inhibition | No Inhibition | No Inhibition | No Inhibition | Inhibition | No Inhibition | Neutral | Inhibition | Inhibition | Inhibition | Inhibition | Inhibition = 3/7  No Inhibition = 4/7 | Inhibition = 3/7  No Inhibition = 4/7 |
| W477R | Inhibition | No Inhibition | No Inhibition | No Inhibition | No Inhibition | No Inhibition | No Inhibition | Inhibition | Inhibition | Neutral | Inhibition | Inhibition | Inhibition | No Inhibition | Inhibition = 4/7  No Inhibition = 3/7 | Inhibition = 2/7  No Inhibition = 5/7 |
| V488M | No Inhibition | Inhibition | No Inhibition | No Inhibition | Inhibition | No Inhibition | No Inhibition | Inhibition | No Inhibition | Inhibition | No Inhibition | Inhibition | No Inhibition | No Inhibition | Inhibition = 1/7  No Inhibition = 6/7 | Inhibition = 4/7  No Inhibition = 3/7 |
| E489K | Inhibition | Neutral | No Inhibition | Inhibition | Inhibition | No Inhibition | No Inhibition | Inhibition | Inhibition | Neutral | Inhibition | No Inhibition | Inhibition | No Inhibition | Inhibition = 5/7  No Inhibition = 2/7 | Inhibition = 2/7  No Inhibition = 5/7 |
| V491L | No Inhibition | No Inhibition | No Inhibition | No Inhibition | Inhibition | No Inhibition | No Inhibition | Inhibition | No Inhibition | No Inhibition | Inhibition | No Inhibition | Inhibition | No Inhibition | Inhibition = 3/7  No Inhibition = 4/7 | Inhibition = 1/7  No Inhibition = 6/7 |
| D494G | Inhibition | Neutral | Inhibition | Inhibition | No Inhibition | Inhibition | No Inhibition | Inhibition | No Inhibition | Neutral | Inhibition | Inhibition | No Inhibition | Inhibition | Inhibition = 3/7  No Inhibition = 4/7 | Inhibition = 5/7  No Inhibition = 2/7 |
| T496A | Inhibition | No Inhibition | No Inhibition | Inhibition | Inhibition | No Inhibition | No Inhibition | Inhibition | No Inhibition | No Inhibition | Inhibition | No Inhibition | No Inhibition | Inhibition | Inhibition = 3/7  No Inhibition = 4/7 | Inhibition = 3/7  No Inhibition = 4/7 |
| R518M | No Inhibition | No Inhibition | No Inhibition | No Inhibition | No Inhibition | No Inhibition | No Inhibition | Inhibition | Inhibition | Neutral | Inhibition | Inhibition | No Inhibition | No Inhibition | Inhibition = 5/7  No Inhibition = 2/7 | Inhibition = 2/7  No Inhibition = 5/7 |
| G561R | No Inhibition | Inhibition | No Inhibition | No Inhibition | No Inhibition | No Inhibition | No Inhibition | Inhibition | No Inhibition | Neutral | Inhibition | Inhibition | Inhibition | No Inhibition | Inhibition = 2/7  No Inhibition = 5/7 | Inhibition = 3/7  No Inhibition = 4/7 |
| P565L | Inhibition | Inhibition | No Inhibition | No Inhibition | Inhibition | Inhibition | No Inhibition | Inhibition | No Inhibition | Neutral | Inhibition | Inhibition | Inhibition | Inhibition | Inhibition = 4/7  No Inhibition = 3/7 | Inhibition = 5/7  No Inhibition = 2/7 |
| K577N | Inhibition | No Inhibition | No Inhibition | Inhibition | No Inhibition | No Inhibition | No Inhibition | Inhibition | Inhibition | Neutral | Inhibition | No Inhibition | Inhibition | Inhibition | Inhibition = 4/7  No Inhibition = 3/7 | Inhibition = 3/7  No Inhibition = 4/7 |
| M579T | Inhibition | Neutral | Neutral | No Inhibition | Inhibition | No Inhibition | No Inhibition | Inhibition | Inhibition | Neutral | Inhibition | Inhibition | Inhibition | Inhibition | Inhibition = 5/7  No Inhibition = 2/7 | Inhibition = 3/7  No Inhibition = 4/7 |
| V581I | Inhibition | Inhibition | No Inhibition | No Inhibition | Inhibition | No Inhibition | No Inhibition | Inhibition | No Inhibition | Neutral | Inhibition | Inhibition | Inhibition | No Inhibition | Inhibition = 4/7  No Inhibition = 3/7 | Inhibition = 3/7  No Inhibition = 4/7 |
| P590L | Inhibition | Neutral | No Inhibition | Inhibition | No Inhibition | No Inhibition | No Inhibition | Inhibition | Inhibition | Neutral | Inhibition | Inhibition | No Inhibition | Inhibition | Inhibition = 3/7  No Inhibition = 4/7 | Inhibition = 4/7  No Inhibition = 3/7 |
| D597E | No Inhibition | No Inhibition | No Inhibition | No Inhibition | Inhibition | No Inhibition | No Inhibition | No Inhibition | Inhibition | Neutral | Inhibition | No Inhibition | Inhibition | Inhibition | Inhibition = 4/7  No Inhibition = 3/7 | Inhibition = 1/7  No Inhibition = 6/7 |
| N599K | No Inhibition | Inhibition | No Inhibition | Inhibition | No Inhibition | No Inhibition | No Inhibition | Inhibition | No Inhibition | Neutral | Inhibition | Inhibition | No Inhibition | No Inhibition | Inhibition = 1/7  No Inhibition = 6/7 | Inhibition = 4/7  No Inhibition = 3/7 |
| K600N | No Inhibition | No Inhibition | No Inhibition | No Inhibition | No Inhibition | No Inhibition | No Inhibition | Inhibition | Inhibition | Neutral | Inhibition | Inhibition | No Inhibition | No Inhibition | Inhibition = 2/7  No Inhibition = 5/7 | Inhibition = 2/7  No Inhibition = 5/7 |
| N601I | Inhibition | Neutral | No Inhibition | No Inhibition | No Inhibition | No Inhibition | No Inhibition | Inhibition | Neutral | Neutral | Inhibition | Inhibition | Inhibition | No Inhibition | Inhibition = 3/7  No Inhibition = 4/7 | Inhibition = 2/7  No Inhibition = 5/7 |
| D609N | Neutral | Inhibition | No Inhibition | No Inhibition | Inhibition | No Inhibition | No Inhibition | No Inhibition | Inhibition | Neutral | Inhibition | Inhibition | Inhibition | Inhibition | Inhibition = 4/7  No Inhibition = 3/7 | Inhibition = 3/7  No Inhibition = 4/7 |
| Y613H | Inhibition | Neutral | No Inhibition | Inhibition | No Inhibition | Inhibition | No Inhibition | Inhibition | No Inhibition | Neutral | Inhibition | Inhibition | Inhibition | No Inhibition | Inhibition = 3/7  No Inhibition = 4/7 | Inhibition = 4/7  No Inhibition = 3/7 |
| D615Y | No Inhibition | No Inhibition | No Inhibition | No Inhibition | Inhibition | No Inhibition | No Inhibition | Inhibition | No Inhibition | No Inhibition | Inhibition | Inhibition | Inhibition | Inhibition | Inhibition = 3/7  No Inhibition = 4/7 | Inhibition = 3/7  No Inhibition = 4/7 |
| I618M | No Inhibition | Inhibition | No Inhibition | No Inhibition | No Inhibition | No Inhibition | No Inhibition | No Inhibition | Inhibition | Neutral | Inhibition | Inhibition | Inhibition | Inhibition | Inhibition = 3/7  No Inhibition = 4/7 | Inhibition = 4/7  No Inhibition = 3/7 |
| K625T | Inhibition | Inhibition | No Inhibition | Inhibition | Inhibition | Inhibition | No Inhibition | Inhibition | No Inhibition | Neutral | Inhibition | Inhibition | Inhibition | Inhibition | Inhibition = 4/7  No Inhibition = 3/7 | Inhibition = 6/7  No Inhibition = 1/7 |
| L628F | No Inhibition | Inhibition | No Inhibition | No Inhibition | No Inhibition | No Inhibition | No Inhibition | Inhibition | No Inhibition | Inhibition | Inhibition | Inhibition | No Inhibition | No Inhibition | Inhibition = 1/7  No Inhibition = 6/7 | Inhibition = 4/7  No Inhibition = 3/7 |
| R644Q | No Inhibition | Neutral | Inhibition | Inhibition | No Inhibition | No Inhibition | No Inhibition | Inhibition | No Inhibition | Neutral | Inhibition | Inhibition | Inhibition | Inhibition | Inhibition = 3/7  No Inhibition = 4/7 | Inhibition = 4/7  No Inhibition = 3/7 |
| E667K | Inhibition | No Inhibition | No Inhibition | Inhibition | No Inhibition | No Inhibition | No Inhibition | Inhibition | No Inhibition | Neutral | Inhibition | Inhibition | Inhibition | Inhibition | Inhibition = 3/7  No Inhibition = 4/7 | Inhibition = 4/7  No Inhibition = 3/7 |
| V670L | No Inhibition | No Inhibition | No Inhibition | No Inhibition | No Inhibition | Inhibition | No Inhibition | Inhibition | No Inhibition | Neutral | Inhibition | Inhibition | Inhibition | Inhibition | Inhibition = 2/7  No Inhibition = 5/7 | Inhibition = 4/7  No Inhibition = 3/7 |
| V672A | Inhibition | Inhibition | No Inhibition | No Inhibition | No Inhibition | No Inhibition | No Inhibition | Inhibition | No Inhibition | Neutral | Inhibition | Inhibition | Inhibition | No Inhibition | Inhibition = 3/7  No Inhibition = 4/7 | Inhibition = 3/7  No Inhibition = 4/7 |
| K676E | Inhibition | No Inhibition | No Inhibition | Inhibition | No Inhibition | No Inhibition | No Inhibition | Inhibition | Inhibition | Neutral | Inhibition | Inhibition | No Inhibition | No Inhibition | Inhibition = 3/7  No Inhibition = 4/7 | Inhibition = 3/7  No Inhibition = 4/7 |
| F683L | No Inhibition | No Inhibition | No Inhibition | Inhibition | No Inhibition | No Inhibition | No Inhibition | Inhibition | Inhibition | Neutral | Inhibition | Inhibition | No Inhibition | No Inhibition | Inhibition = 2/7  No Inhibition = 5/7 | Inhibition = 3/7  No Inhibition = 4/7 |
| S692F | Inhibition | No Inhibition | No Inhibition | No Inhibition | No Inhibition | No Inhibition | No Inhibition | Inhibition | Inhibition | No Inhibition | Inhibition | Inhibition | Inhibition | Inhibition | Inhibition = 4/7  No Inhibition = 3/7 | Inhibition = 3/7  No Inhibition = 4/7 |
| D693N | No Inhibition | No Inhibition | No Inhibition | No Inhibition | No Inhibition | No Inhibition | No Inhibition | Inhibition | Inhibition | Neutral | Inhibition | No Inhibition | No Inhibition | No Inhibition | Inhibition = 2/7  No Inhibition = 5/7 | Inhibition = 1/7  No Inhibition = 6/7 |
| I694M | Inhibition | Inhibition | Inhibition | Inhibition | No Inhibition | No Inhibition | No Inhibition | Neutral | Inhibition | Neutral | No Inhibition | Inhibition | Inhibition | Inhibition | Inhibition = 4/7  No Inhibition = 3/7 | Inhibition = 4/7  No Inhibition = 3/7 |
| E701K | Inhibition | No Inhibition | No Inhibition | Inhibition | No Inhibition | No Inhibition | No Inhibition | Inhibition | Inhibition | Neutral | Inhibition | Inhibition | No Inhibition | No Inhibition | Inhibition = 3/7  No Inhibition = 4/7 | Inhibition = 3/7  No Inhibition = 4/7 |
| R708Q | Inhibition | Neutral | No Inhibition | Inhibition | Inhibition | No Inhibition | No Inhibition | Inhibition | Inhibition | Neutral | Inhibition | Inhibition | Inhibition | No Inhibition | Inhibition = 5/7  No Inhibition = 2/7 | Inhibition = 3/7  No Inhibition = 4/7 |
| D713N | No Inhibition | No Inhibition | No Inhibition | No Inhibition | No Inhibition | No Inhibition | No Inhibition | Inhibition | No Inhibition | Neutral | Inhibition | Inhibition | No Inhibition | No Inhibition | Inhibition = 1/7  No Inhibition = 6/7 | Inhibition = 2/7  No Inhibition = 5/7 |
| R716H | Inhibition | Neutral | No Inhibition | Inhibition | No Inhibition | No Inhibition | No Inhibition | Inhibition | No Inhibition | Neutral | Inhibition | Inhibition | Inhibition | No Inhibition | Inhibition = 3/7  No Inhibition = 4/7 | Inhibition = 3/7  No Inhibition = 4/7 |
| R716C | Inhibition | Inhibition | No Inhibition | Inhibition | No Inhibition | No Inhibition | No Inhibition | Inhibition | No Inhibition | Inhibition | Inhibition | Inhibition | Inhibition | No Inhibition | Inhibition = 3/7  No Inhibition = 4/7 | Inhibition = 5/7  No Inhibition = 2/7 |
| N720S | Inhibition | Inhibition | No Inhibition | Inhibition | Inhibition | Inhibition | No Inhibition | Inhibition | Inhibition | Neutral | Inhibition | Inhibition | Inhibition | No Inhibition | Inhibition = 5/7  No Inhibition = 2/7 | Inhibition = 5/7  No Inhibition =2/7 |
| P737H | No Inhibition | Inhibition | No Inhibition | Inhibition | No Inhibition | No Inhibition | No Inhibition | Inhibition | Inhibition | Inhibition | No Inhibition | No Inhibition | No Inhibition | Inhibition | Inhibition = 1/7  No Inhibition = 6/7 | Inhibition = 5/7  No Inhibition = 2/7 |
| P737L | No Inhibition | Inhibition | Inhibition | No Inhibition | No Inhibition | No Inhibition | No Inhibition | Inhibition | Inhibition | Neutral | Inhibition | Inhibition | No Inhibition | No Inhibition | Inhibition = 3/7  No Inhibition = 4/7 | Inhibition = 3/7  No Inhibition = 4/7 |
| V748F | Inhibition | Inhibition | No Inhibition | No Inhibition | Inhibition | Inhibition | No Inhibition | Inhibition | Inhibition | No Inhibition | No Inhibition | Inhibition | No Inhibition | Inhibition | Inhibition = 3/7  No Inhibition = 4/7 | Inhibition = 5/7  No Inhibition = 2/7 |
| L760M | Inhibition | No Inhibition | Inhibition | No Inhibition | No Inhibition | No Inhibition | No Inhibition | Inhibition | Inhibition | Neutral | Inhibition | No Inhibition | No Inhibition | No Inhibition | Inhibition = 4/7  No Inhibition = 3/7 | Inhibition = 1/7  No Inhibition = 6/7 |
| I761T | Inhibition | Inhibition | No Inhibition | Inhibition | Inhibition | No Inhibition | No Inhibition | Inhibition | No Inhibition | Inhibition | Inhibition | Inhibition | Inhibition | Inhibition | Inhibition = 4/7  No Inhibition = 3/7 | Inhibition = 6/7  No Inhibition = 1/7 |
| F762L | Inhibition | Inhibition | No Inhibition | Inhibition | No Inhibition | No Inhibition | No Inhibition | Inhibition | No Inhibition | No Inhibition | Inhibition | Inhibition | Inhibition | No Inhibition | Inhibition = 3/7  No Inhibition = 4/7 | Inhibition = 4/7  No Inhibition = 3/7 |
| G764R | No Inhibition | Inhibition | No Inhibition | No Inhibition | No Inhibition | No Inhibition | No Inhibition | Inhibition | Inhibition | No Inhibition | Inhibition | No Inhibition | No Inhibition | Inhibition | Inhibition = 2/7  No Inhibition = 5/7 | Inhibition = 3/7  No Inhibition = 4/7 |
| R766K | No Inhibition | Inhibition | No Inhibition | Inhibition | No Inhibition | No Inhibition | No Inhibition | Inhibition | No Inhibition | Inhibition | Inhibition | Inhibition | Inhibition | No Inhibition | Inhibition = 2/7  No Inhibition = 5/7 | Inhibition = 5/7  No Inhibition = 2/7 |
| R768L | Inhibition | No Inhibition | No Inhibition | No Inhibition | Inhibition | No Inhibition | No Inhibition | Inhibition | No Inhibition | No Inhibition | Inhibition | Inhibition | Inhibition | No Inhibition | Inhibition = 4/7  No Inhibition = 3/7 | Inhibition = 2/7  No Inhibition = 5/7 |
| R768W | No Inhibition | No Inhibition | No Inhibition | Inhibition | No Inhibition | No Inhibition | No Inhibition | Inhibition | Inhibition | No Inhibition | No Inhibition | Inhibition | No Inhibition | Inhibition | Inhibition = 1/7  No Inhibition = 6/7 | Inhibition = 4/7  No Inhibition = 3/7 |
| R775I | Neutral | Neutral | Inhibition | Inhibition | Neutral | Neutral | Neutral | Neutral | Neutral | Neutral | Neutral | Neutral | Neutral | Neutral | Inhibition = 1/7  No Inhibition = 6/7 | Inhibition = 1/7  No Inhibition = 6/7 |
| P780S | Neutral | Neutral | Inhibition | Inhibition | Neutral | Neutral | Neutral | Neutral | Neutral | Neutral | No Inhibition | Neutral | Neutral | Neutral | Inhibition = 1/7  No Inhibition = 6/7 | Inhibition = 1/7  No Inhibition = 6/7 |
| D785N | Neutral | Neutral | Inhibition | Inhibition | Neutral | Neutral | Neutral | Neutral | Neutral | Neutral | Neutral | Neutral | Neutral | Neutral | Inhibition = 1/7  No Inhibition = 6/7 | Inhibition = 1/7  No Inhibition = 6/7 |
| G789R | Neutral | Neutral | Inhibition | Inhibition | Neutral | Neutral | Neutral | Neutral | Neutral | Neutral | Neutral | Neutral | Neutral | Neutral | Inhibition = 1/7  No Inhibition = 6/7 | Inhibition = 1/7  No Inhibition = 6/7 |
| T798P | Neutral | Neutral | Neutral | Inhibition | Neutral | Neutral | Neutral | Neutral | Neutral | Neutral | Neutral | Neutral | Neutral | Neutral | Inhibition = 0/7  No Inhibition = 7/7 | Inhibition = 1/7  No Inhibition = 6/7 |
| T803I | Neutral | Neutral | Inhibition | Inhibition | Neutral | Neutral | Neutral | Neutral | Neutral | Neutral | Neutral | Neutral | Neutral | Neutral | Inhibition = 1/7  No Inhibition = 6/7 | Inhibition = 1/7  No Inhibition = 6/7 |
| S47C, P284S | Inhibition | Inhibition | No Inhibition | No Inhibition | No Inhibition | No Inhibition | No Inhibition | Inhibition | Inhibition | Neutral | Inhibition | Inhibition | Inhibition | No Inhibition | Inhibition = 4/7  No Inhibition = 3/7 | Inhibition = 3/7  No Inhibition = 4/7 |
| E145K, E639K | No Inhibition | Inhibition | Inhibition | Inhibition | Inhibition | No Inhibition | No Inhibition | Inhibition | Inhibition | Neutral | Inhibition | Inhibition | Inhibition | No Inhibition | Inhibition = 5/7  No Inhibition = 2/7 | Inhibition = 4/7  No Inhibition = 3/7 |
| W302G, F400L | No Inhibition | No Inhibition | No Inhibition | No Inhibition | No Inhibition | Inhibition | No Inhibition | Inhibition | Inhibition | No Inhibition | Inhibition | Inhibition | No Inhibition | Inhibition | Inhibition = 2/7  No Inhibition = 5/7 | Inhibition = 4/7  No Inhibition = 3/7 |
| T593I, P729S | Inhibition | No Inhibition | No Inhibition | Inhibition | Inhibition | Inhibition | No Inhibition | Inhibition | No Inhibition | Neutral | No Inhibition | No Inhibition | Inhibition | Inhibition | Inhibition = 3/7  No Inhibition = 4/7 | Inhibition = 4/7  No Inhibition = 3/7 |
| H195Y, F683L | Inhibition | No Inhibition | No Inhibition | No Inhibition | No Inhibition | No Inhibition | No Inhibition | Inhibition | Inhibition | Neutral | Inhibition | Inhibition | No Inhibition | No Inhibition | Inhibition = 3/7  No Inhibition = 4/7 | Inhibition = 2/7  No Inhibition = 5/7 |
| K131Q, F683L | Inhibition | Neutral | No Inhibition | Inhibition | No Inhibition | Inhibition | No Inhibition | Inhibition | Inhibition | Neutral | Inhibition | Inhibition | No Inhibition | Inhibition | Inhibition = 3/7  No Inhibition = 4/7 | Inhibition = 5/7  No Inhibition = 2/7 |
| L120I, V658L | Inhibition | No Inhibition | No Inhibition | No Inhibition | No Inhibition | Inhibition | No Inhibition | Inhibition | Neutral | No Inhibition | Inhibition | Inhibition | Inhibition | Inhibition | Inhibition = 3/7  No Inhibition = 4/7 | Inhibition = 4/7  No Inhibition = 3/7 |
| S280Y, Q598H | Inhibition | No Inhibition | No Inhibition | No Inhibition | No Inhibition | No Inhibition | No Inhibition | Inhibition | No Inhibition | Neutral | Inhibition | Inhibition | Inhibition | Inhibition | Inhibition = 3/7  No Inhibition = 4/7 | Inhibition = 3/7  No Inhibition = 4/7 |
| D427N, A576T | No Inhibition | Inhibition | No Inhibition | No Inhibition | No Inhibition | Inhibition | No Inhibition | Inhibition | Inhibition | Neutral | Inhibition | Inhibition | No Inhibition | No Inhibition | Inhibition = 2/7  No Inhibition = 5/7 | Inhibition = 4/7  No Inhibition = 3/7 |
| W48L, N437H | Inhibition | Neutral | No Inhibition | Inhibition | No Inhibition | No Inhibition | No Inhibition | Inhibition | Inhibition | No Inhibition | Inhibition | Inhibition | No Inhibition | Inhibition | Inhibition = 3/7  No Inhibition = 4/7 | Inhibition = 4/7  No Inhibition = 3/7 |
| P336S, K26N | Inhibition | No Inhibition | No Inhibition | No Inhibition | No Inhibition | No Inhibition | No Inhibition | Inhibition | No Inhibition | Neutral | Inhibition | No Inhibition | No Inhibition | No Inhibition | Inhibition = 2/7  No Inhibition = 5/7 | Inhibition = 1/7  No Inhibition = 6/7 |
| N578S, Y497C | Inhibition | Neutral | No Inhibition | Inhibition | No Inhibition | No Inhibition | No Inhibition | Inhibition | Inhibition | Neutral | Inhibition | No Inhibition | Inhibition | Inhibition | Inhibition = 4/7  No Inhibition = 3/7 | Inhibition = 3/7  No Inhibition = 4/7 |
| P178S, E182D, D427N | Inhibition | Inhibition | No Inhibition | No Inhibition | No Inhibition | Inhibition | No Inhibition | Inhibition | No Inhibition | Neutral | Inhibition | Inhibition | Inhibition | Inhibition | Inhibition = 3/7  No Inhibition = 4/7 | Inhibition = 5/7  No Inhibition = 2/7 |
| R169I, H195Y, N394H | Inhibition | No Inhibition | No Inhibition | Inhibition | Inhibition | No Inhibition | No Inhibition | Inhibition | No Inhibition | Neutral | Inhibition | Inhibition | No Inhibition | Inhibition | Inhibition = 3/7  No Inhibition = 4/7 | Inhibition = 4/7  No Inhibition = 3/7 |
| N194K, R306I, E479D | No Inhibition | No Inhibition | No Inhibition | No Inhibition | Inhibition | No Inhibition | No Inhibition | Inhibition | No Inhibition | Neutral | Inhibition | Inhibition | Inhibition | No Inhibition | Inhibition = 3/7  No Inhibition = 4/7 | Inhibition = 2/7  No Inhibition = 5/7 |
| S128I, R169I, S602Y | Inhibition | Neutral | No Inhibition | Inhibition | Inhibition | Inhibition | No Inhibition | Inhibition | Inhibition | Neutral | Inhibition | Inhibition | Inhibition | No Inhibition | Inhibition = 5/7  No Inhibition = 2/7 | Inhibition = 4/7  No Inhibition = 3/7 |
| M82T, F314L, K600N | Inhibition | Inhibition | No Inhibition | Inhibition | No Inhibition | No Inhibition | No Inhibition | Inhibition | No Inhibition | Neutral | Inhibition | No Inhibition | Inhibition | Inhibition | Inhibition = 3/7  No Inhibition = 4/7 | Inhibition = 4/7  No Inhibition = 3/7 |
| E375D, K577N, R768W | Inhibition | Neutral | No Inhibition | Inhibition | No Inhibition | No Inhibition | No Inhibition | Inhibition | Neutral | Neutral | Inhibition | Inhibition | Inhibition | No Inhibition | Inhibition = 3/7  No Inhibition = 4/7 | Inhibition = 3/7  No Inhibition = 4/7 |
| Q18K, G268C, W610L | Neutral | Neutral | Inhibition | Inhibition | Neutral | Neutral | Neutral | Neutral | Neutral | Neutral | Neutral | Neutral | Neutral | Neutral | Inhibition = 1/7  No Inhibition = 6/7 | Inhibition = 1/7  No Inhibition = 6/7 |
| A25V, A396T, I679N | Inhibition | No Inhibition | No Inhibition | No Inhibition | No Inhibition | Inhibition | No Inhibition | Inhibition | No Inhibition | Neutral | Inhibition | Inhibition | No Inhibition | No Inhibition | Inhibition = 2/7  No Inhibition = 5/7 | Inhibition = 3/7  No Inhibition = 4/7 |
| R393I, E571G, R768W | No Inhibition | No Inhibition | No Inhibition | Inhibition | No Inhibition | No Inhibition | No Inhibition | Inhibition | No Inhibition | Neutral | Inhibition | Inhibition | No Inhibition | Inhibition | Inhibition = 1/7  No Inhibition = 6/7 | Inhibition = 4/7  No Inhibition = 3/7 |
| **Common BA between SARS-CoV and SARS-CoV-2** | **Inhibition: 28**  **No Inhibition: 30**  **Total common between SARS-CoV& SARS-CoV-2: 58** | | **Inhibition: 18**  **No Inhibition: 76**  **Total common between SARS-CoV& SARS-CoV-2: 94** | | **Inhibition: 12**  **No Inhibition: 77**  **Total common between SARS-CoV& SARS-CoV-2: 89** | | **Inhibition: 00**  **No Inhibition: 06**  **Total common between SARS-CoV& SARS-CoV-2: 06** | | **Inhibition: 9**  **No Inhibition: 10**  **Total common between SARS-CoV& SARS-CoV-2: 19** | | **Inhibition: 113**  **No Inhibition: 02**  **Total common between SARS-CoV& SARS-CoV-2: 115** | | **Inhibition: 55**  **No Inhibition: 25**  **Total common between SARS-CoV& SARS-CoV-2: 80** | |  |  |
| **Inference Score correlated with ClusPro** | Inhibition: 85/85  No inhibition: 70/70 | Inhibition: 56/56  No inhibition: 99/99 | Inhibition: 10/85  No inhibition: 53/70  Tot: 63/155 | Inhibition: 22/56  No inhibition: 52/99  Tot: 74/155 | Inhibition: 26/85  No inhibition: 52/70  Tot: 78/155 | Inhibition: 17/56  No inhibition: 79/99  Tot: 96/155 | Inhibition: 0/85  No inhibition: 70/70**(H)**  Tot: 70/155 | Inhibition: 51/56**(H)**  No inhibition: 12/99  Tot: 63/155 | Inhibition: 36/85  No inhibition: 39/70  Tot: 75/155 | Inhibition: 14/56  No inhibition: 91/99**(H)**  Tot: 105/155 | Inhibition: 80/85**(H)**  No inhibition: 14/70  Tot: 94/155 | Inhibition: 49/56  No inhibition: 27/99  Tot: 76/155 | Inhibition: 63/85  No inhibition: 34/70  Tot: 97/155 | Inhibition: 33/56  No inhibition: 56/99  Tot: 89/155 |  |  |
|  | CoV | CoV-2 | CoV | CoV-2 | CoV | CoV-2 | CoV | CoV-2 | CoV | CoV-2 | CoV | CoV-2 | CoV | CoV-2 |  |  |
|  | **ClusPro** | | **ZDOCK** | | **HDOCK** | | **PATCHDOCK** | | **InterEvDock2** | | **SOAP PP** | | **FRODOCK 2** | |  |  |

**Supplementary Table 5.**

| **Mutants** | **ZDOCK** | | **ClusPro** | | **HDOCK** | | **PatchDock** | | **InterEvDock2** | | **SOAP PP** | | **FRODOCK2** | | **INFERENCE** | |
| --- | --- | --- | --- | --- | --- | --- | --- | --- | --- | --- | --- | --- | --- | --- | --- | --- |
|  | **SARS-CoV** | **SARS-CoV-2** | **SARS-CoV** | **SARS-CoV-2** | **SARS-CoV** | **SARS-CoV-2** | **SARS-CoV** | **SARS-CoV-2** | **SARS-CoV** | **SARS-CoV-2** | **SARS-CoV** | **SARS-CoV-2** | **SARS-CoV** | **SARS-CoV-2** | **SARS-CoV** | **SARS-CoV-2** |
| WILD TYPE | **Binds with SARS-CoV** | **Binds with SARS-CoV-2** | **Binds with SARS-CoV** | **Binds with SARS-CoV-2** | **Binds with SARS-CoV** | **Binds with SARS-CoV-2** | **Binds with SARS-CoV** | **Binds with SARS-CoV-2** | **Binds with SARS-CoV** | **Binds with SARS-CoV-2** | **Binds with SARS-CoV** | **Binds with SARS-CoV-2** | **Binds with SARS-CoV** | **Binds with SARS-CoV-2** |  |  |
| S5F | No inhibition | No inhibition | Neutral | Neutral | Neutral | Neutral | Neutral | Neutral | Neutral | Neutral | Neutral | Neutral | Neutral | Neutral | Inhibition= 0/7  No inhibition= 7/7 | Inhibition= 0/7  No inhibition= 7/7 |
| A25V | No inhibition | No inhibition | Inhibition | No inhibition | No inhibition | Inhibition | No inhibition | Inhibition | No inhibition | Neutral | Inhibition | Inhibition | No inhibition | No inhibition | Inhibition= 2/7  No inhibition= 5/7 | Inhibition= 3/7  No inhibition= 4/7 |
| L100V | No inhibition | Inhibition | Inhibition | Inhibition | No inhibition | No inhibition | No inhibition | Inhibition | No inhibition | Neutral | Inhibition | Inhibition | Inhibition | Inhibition | Inhibition= 3/7  No inhibition= 4/7 | Inhibition= 5/7  No inhibition= 2/7 |
| V184A | No inhibition | Inhibition | Inhibition | Neutral | No inhibition | No inhibition | No inhibition | Inhibition | No inhibition | Neutral | Inhibition | No inhibition | Inhibition | Inhibition | Inhibition= 3/7  No inhibition= 4/7 | Inhibition= 3/7  No inhibition= 4/7 |
| S218N | Inhibition | No inhibition | Inhibition | Inhibition | No inhibition | No inhibition | No inhibition | No inhibition | No inhibition | Neutral | Inhibition | Inhibition | No inhibition | No inhibition | Inhibition= 3/7  No inhibition= 4/7 | Inhibition= 2/7  No inhibition= 5/7 |
| Y252C | No inhibition | Inhibition | Inhibition | Inhibition | No inhibition | No inhibition | No inhibition | Inhibition | No inhibition | Neutral | Inhibition | Inhibition | No inhibition | No inhibition | Inhibition= 2/7  No inhibition= 5/7 | Inhibition= 4/7  No inhibition= 3/7 |
| P253T | No inhibition | No inhibition | Inhibition | No inhibition | No inhibition | No inhibition | No inhibition | Inhibition | No inhibition | No inhibition | Inhibition | Inhibition | Inhibition | No inhibition | Inhibition= 3/7  No inhibition= 4/7 | Inhibition= 2/7  No inhibition= 5/7 |
| T276K | No inhibition | No inhibition | Inhibition | Inhibition | No inhibition | No inhibition | No inhibition | Inhibition | No inhibition | Neutral | Inhibition | Inhibition | Inhibition | No inhibition | Inhibition= 3/7  No inhibition= 4/7 | Inhibition= 3/7  No inhibition= 4/7 |
| N322I | No inhibition | Inhibition | Inhibition | Inhibition | No inhibition | No inhibition | No inhibition | Inhibition | Inhibition | Neutral | Inhibition | Inhibition | Inhibition | No inhibition | Inhibition= 4/7  No inhibition= 3/7 | Inhibition= 4/7  No inhibition= 3/7 |
| T334A | No inhibition | Inhibition | Inhibition | Neutral | No inhibition | No inhibition | Inhibition | Inhibition | No inhibition | Neutral | Inhibition | Inhibition | No inhibition | Inhibition | Inhibition= 3/7  No inhibition= 4/7 | Inhibition= 4/7  No inhibition= 3/7 |
| A413V | No inhibition | No inhibition | Inhibition | Inhibition | Inhibition | No inhibition | No inhibition | Inhibition | No inhibition | Neutral | Inhibition | Inhibition | Inhibition | No inhibition | Inhibition= 4/7  No inhibition= 3/7 | Inhibition= 3/7  No inhibition= 4/7 |
| K416N | No inhibition | No inhibition | Inhibition | No inhibition | No inhibition | No inhibition | No inhibition | Inhibition | Inhibition | Neutral | Inhibition | Inhibition | Inhibition | No inhibition | Inhibition= 4/7  No inhibition= 3/7 | Inhibition= 2/7  No inhibition= 5/7 |
| P426L | No inhibition | No inhibition | Inhibition | Inhibition | No inhibition | Inhibition | No inhibition | Inhibition | No inhibition | Neutral | Inhibition | Inhibition | Inhibition | Inhibition | Inhibition= 3/7  No inhibition= 4/7 | Inhibition= 5/7  No inhibition= 2/7 |
| E457K | No inhibition | No inhibition | No inhibition | No inhibition | No inhibition | Inhibition | No inhibition | Inhibition | No inhibition | Neutral | Inhibition | No inhibition | No inhibition | No inhibition | Inhibition= 1/7  No inhibition= 6/7 | Inhibition= 2/7  No inhibition= 5/7 |
| Q472P | No inhibition | No inhibition | Inhibition | No inhibition | No inhibition | No inhibition | No inhibition | Inhibition | No inhibition | Neutral | Inhibition | Inhibition | Inhibition | Inhibition | Inhibition= 3/7  No inhibition= 4/7 | Inhibition= 3/7  No inhibition= 4/7 |
| P612L | No inhibition | No inhibition | Inhibition | No inhibition | No inhibition | No inhibition | No inhibition | Inhibition | No inhibition | Neutral | Inhibition | Inhibition | Inhibition | Inhibition | Inhibition= 2/7  No inhibition= 5/7 | Inhibition= 2/7  No inhibition= 5/7 |
| W635L | No inhibition | No inhibition | Inhibition | Inhibition | No inhibition | No inhibition | No inhibition | Inhibition | No inhibition | Inhibition | Inhibition | Inhibition | Inhibition | Inhibition | Inhibition= 3/7  No inhibition= 4/7 | Inhibition= 4/7  No inhibition= 3/7 |
| Y649C | No inhibition | Inhibition | Inhibition | Neutral | No inhibition | No inhibition | No inhibition | Inhibition | No inhibition | Inhibition | Inhibition | Inhibition | Inhibition | Inhibition | Inhibition= 2/7  No inhibition= 5/7 | Inhibition= 5/7  No inhibition= 1/7 |
| E668K | Inhibition | Inhibition | No inhibition | No inhibition | No inhibition | Inhibition | No inhibition | Inhibition | Inhibition | Neutral | Inhibition | No inhibition | Inhibition | Inhibition | Inhibition= 4/7  No inhibition= 3/7 | Inhibition= 4/7  No inhibition= 3/7 |
| A782V | No inhibition | Inhibition | Neutral | Neutral | Neutral | Neutral | Neutral | Neutral | Neutral | Neutral | Neutral | Neutral | Neutral | Neutral | Inhibition= 0/7  No inhibition= 1/7 | Inhibition=1/7  No inhibition= 6/7 |
| A386T, F314I | No inhibition | Inhibition | Inhibition | Inhibition | No inhibition | No inhibition | No inhibition | Inhibition | No inhibition | Neutral | Inhibition | Inhibition | Inhibition | Inhibition | Inhibition= 3/7  No inhibition= 4/7 | Inhibition= 5/7  No inhibition= 2/7 |
| F603C, K619N | Inhibition | No inhibition | Inhibition | Inhibition | No inhibition | No inhibition | No inhibition | No inhibition | No inhibition | Inhibition | Inhibition | Inhibition | Inhibition | Inhibition | Inhibition= 4/7  No inhibition= 3/7 | Inhibition= 4/7  No inhibition= 3/7 |
| F314L, Y510H | No inhibition | Inhibition | Neutral | No inhibition | No inhibition | No inhibition | No inhibition | Inhibition | No inhibition | Neutral | Inhibition | Inhibition | Inhibition | No inhibition | Inhibition= 2/7  No inhibition= 5/7 | Inhibition= 3/7  No inhibition= 4/7 |
| L664I, D382Y | No inhibition | Inhibition | Inhibition | Neutral | No inhibition | No inhibition | No inhibition | Inhibition | No inhibition | No inhibition | Inhibition | No inhibition | Inhibition | Inhibition | Inhibition= 3/7  No inhibition= 4/7 | Inhibition= 3/7  No inhibition= 4/7 |
| E145K, E495K, I233S | Inhibition | Inhibition | No inhibition | Inhibition | Inhibition | No inhibition | No inhibition | Inhibition | Inhibition | Neutral | Inhibition | Inhibition | Inhibition | No inhibition | Inhibition= 5/7  No inhibition= 2/7 | Inhibition= 4/7  No inhibition= 3/7 |
| **Common BA between SARS-CoV and SARS-CoV-2** | Inhibition: 02  No Inhibition: 11  Total common between SARS-CoV& SARS-CoV-2: 13 | | Inhibition: 10  No Inhibition: 05  Total common between SARS-CoV& SARS-CoV-2: 12 | | Inhibition: 0  No Inhibition: 19  Total common between SARS-CoV& SARS-CoV-2: 17 | | Inhibition: 01  No Inhibition: 04  Total common between SARS-CoV& SARS-CoV-2: 03 | | Inhibition: 0  No Inhibition: 18  Total common between SARS-CoV& SARS-CoV-2: 02 | | Inhibition: 18  No Inhibition: 02  Total common between SARS-CoV& SARS-CoV-2: 18 | | Inhibition: 08  No Inhibition: 06  Total common between SARS-CoV& SARS-CoV-2: 12 | |  | |
| **Inference Score correlated with ClusPro** | Inhibition: 4/25  No Inhibition: 21/25 | Inhibition: 12/25  No Inhibition: 13/25 | Inhibition: 19/25  No Inhibition: 6/25 | Inhibition: 11/25  No Inhibition: 14/25 | Inhibition: 2/25  No Inhibition: 23/25 | Inhibition: 4/25  No Inhibition: 21/25 | Inhibition: 1/25  No Inhibition: 24/25 | Inhibition: 21/25  No Inhibition: 4/25 | Inhibition: 4/25  No Inhibition: 21/25 | Inhibition: 3/25  No Inhibition: 22/25 | Inhibition: 23/25  No Inhibition: 2/25 | Inhibition: 19/25  No Inhibition: 7/25 | Inhibition: 16/25  No Inhibition: 9/25 | Inhibition: 11/25  No Inhibition: 14/25 |  | |
|  | CoV | CoV-2 | CoV | CoV-2 | CoV | CoV-2 | CoV | CoV-2 | CoV | CoV-2 | CoV | CoV-2 | CoV | CoV-2 |  | |
|  | **ZDOCK** | | **ClusPro** | | **HDOCK** | | **PATCHDOCK** | | **InterEvDock2** | | **SOAP PP** | | **FRODOCK 2** | |  | |
